# Stable Luminescent [Cu(NN)(PP)]^+^ Complexes Incorporating a β‐Cyclodextrin‐Based Diphosphane Ligand with Metal‐Confining Properties

**DOI:** 10.1002/anie.202214638

**Published:** 2022-12-16

**Authors:** Tuan‐Anh Phan, Nicola Armaroli, Alejandra Saavedra Moncada, Elisa Bandini, Béatrice Delavaux‐Nicot, Jean‐François Nierengarten, Dominique Armspach

**Affiliations:** ^1^ Équipe Confinement Moléculaire et Catalyse Université de Strasbourg Institut de Chimie de Strasbourg, UMR 7177 CNRS 4 rue Blaise Pascal CS90032, 67081 Strasbourg Cedex France; ^2^ Istituto per la Sintesi Organica e la Fotoreattività, Consiglio Nazionale delle Ricerche Via P. Gobetti 101 40129 Bologna Italy; ^3^ Laboratoire de Chimie de Coordination du CNRS (LCC) UPR 8241 Université de Toulouse (UPS) 205 route de Narbonne 31077 Toulouse Cedex 4 France; ^4^ Laboratoire de Chimie des Matériaux Moléculaires Université de Strasbourg et CNRS (LIMA - UMR 7042), Ecole Européenne de Chimie, Polymères et Matériaux 25 rue Becquerel 67087 Strasbourg Cedex 2 France

**Keywords:** Copper, Cyclodextrins, Diimines, Luminescence, Phosphanes

## Abstract

A β‐cyclodextrin‐based diphosphane with metal‐confining properties was efficiently synthesized thanks to an unprecedented Smiles‐like rearrangement of diphenyl‐(2‐phosphanylphenyl)phosphane in the presence of excess *n*‐BuLi. The *cis*‐chelating bidentate ligand is capable of forming very stable heteroleptic [Cu(NN)(PP)]^+^ complexes in which a metal‐bound diimine ligand (bpy, phen, or mmp) is located within the cyclodextrin cavity. As a result of ligand encapsulation, flattening of the metal tetrahedral geometry in the excited state is disfavored, thereby resulting in enhanced luminescent properties.

## Graphical abstract

An unprecedented Smiles‐like rearrangement of diphenyl‐(2‐phosphanylphenyl)phosphane gives access to a β‐cyclodextrin‐based diphosphane that forms stable heteroleptic [Cu (NN) (PP)]^+^ complexes. Unfavorable flattening of the metal tetrahedral geometry in the excited state as a result of diimine encapsulation (bpy, phen, or mmp) is responsible for the enhanced luminescent properties.

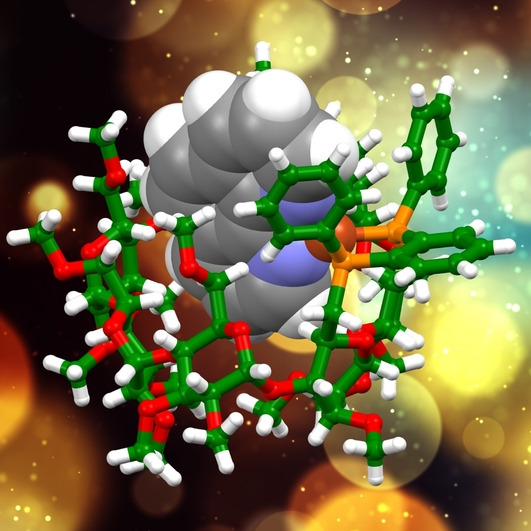

## Introduction

Owing to their outstanding photophysical properties, cationic Cu^I^ complexes with aromatic diimine ligands of the bpy family (bpy: 2,2′‐bipyridine) and diphosphanes have been intensively investigated in the past decades.^[1]^ In particular,^[2]^ their high emission quantum yields, which originate from long‐lived metal‐to‐ligand charge transfer (MLCT) excited states, have made them attractive for applications requiring luminescent materials.^[3]^ They also proved effective in photoredox catalysis, where they can replace more classical and expensive complexes based on platinum group elements.^[4]^ Despite these remarkable achievements, such heteroleptic complexes are often only stable in the solid state and an equilibrium between the homoleptic and the heteroleptic complexes is observed in solution.^[5]^ This kinetic instability represents a major limitation for the preparation of robust [Cu(NN)(PP)]^+^ derivatives. A strategy to overtake this problem entails the use of macrocyclic NN ligands which, because of the maximum site occupancy principle, tend to form pseudo‐rotaxane‐like heteroleptic complexes with improved stability.^[6]^ Upon light excitation, flattening of the metal coordination sphere of tetrahedral Cu^I^ complexes occurs in the excited state. This distortion facilitates non‐emissive deactivation pathways and shortens excited‐state lifetimes. So far, no macrocyclic phosphorus ligands capable of preventing the reorganization of the metal coordination sphere and boost the stability and luminescent properties of hereroleptic Cu^I^ complexes have been devised. Inspired by our previous studies using macrocyclic phenanthroline ligands, we now report the synthesis of a new metal‐confining cavity‐shaped diphosphane system, which is unable to form homoleptic Cu^I^ complexes. In this case, the chelating unit is not incorporated in the macrocyclic structure but located on one rim of a cyclodextrin (CD) scaffold. In this way, its *cis*‐chelating character forces the encapsulation of a tetrahedrally coordinated Cu^I^ diimine fragment, which therefore hinders flattening upon light excitation. The implications of rigid encapsulation of the metal unit on the photophysical and electrochemical properties of the complexes are then thoroughly discussed.

## Results and Discussion

**Scheme 1:**
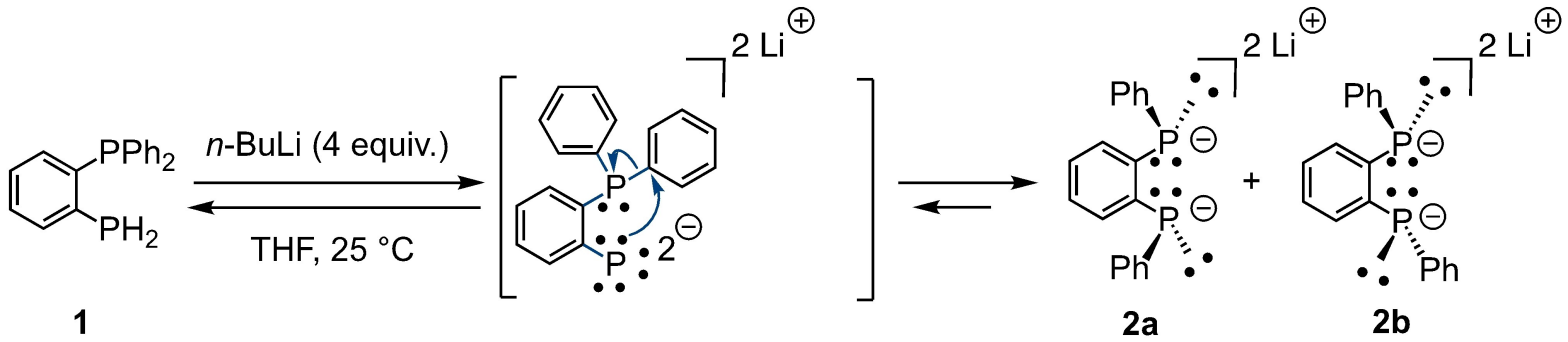
Smiles‐like rearrangement of diphosphane **1** in the presence of excess *n*‐BuLi

**Scheme 2:**
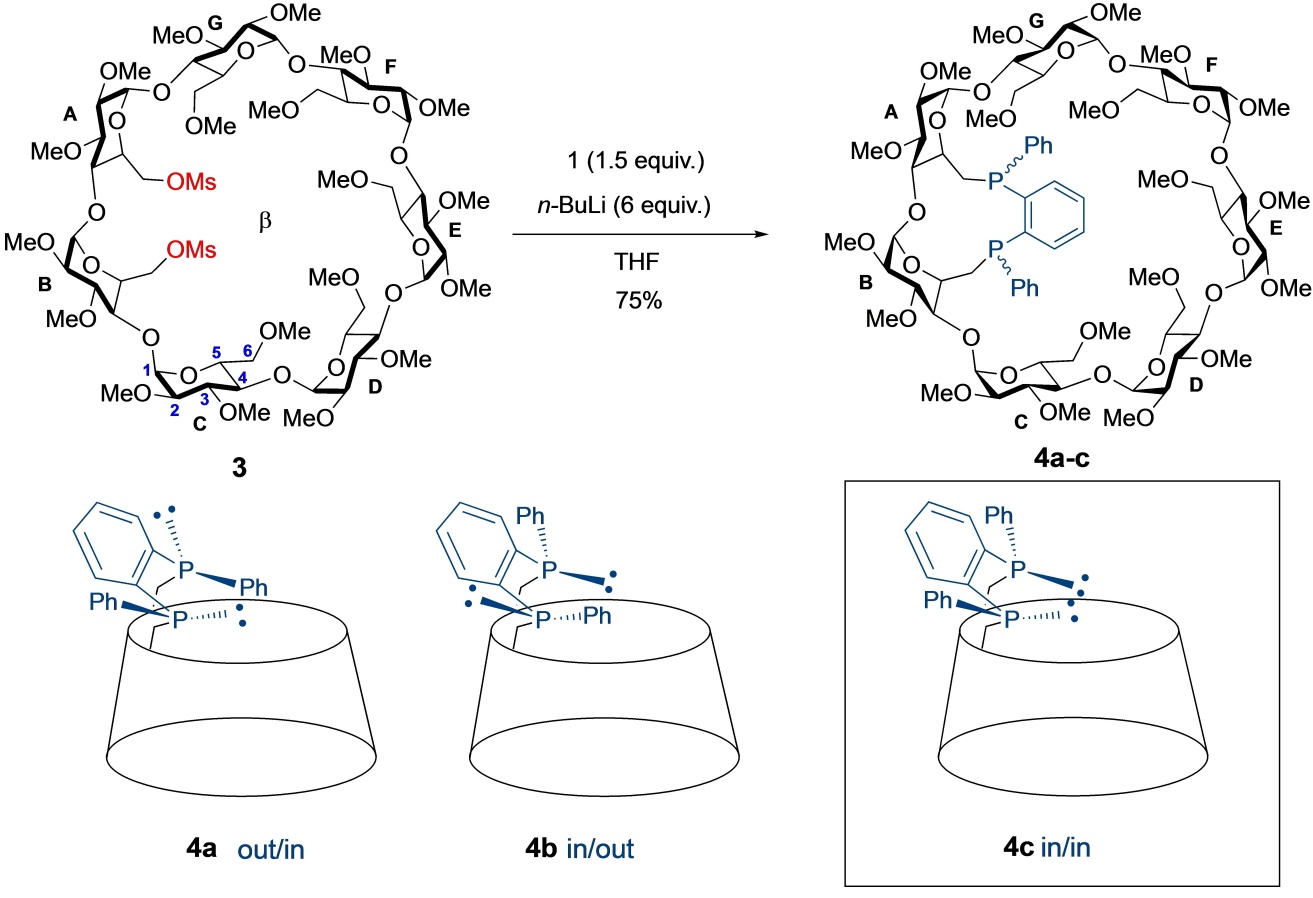
Rigid capping of dimesylate **3** with diphosphanide **2 a,b** resulting in the formation of diastereomers **4 a**–**c**.

**Scheme 3:**
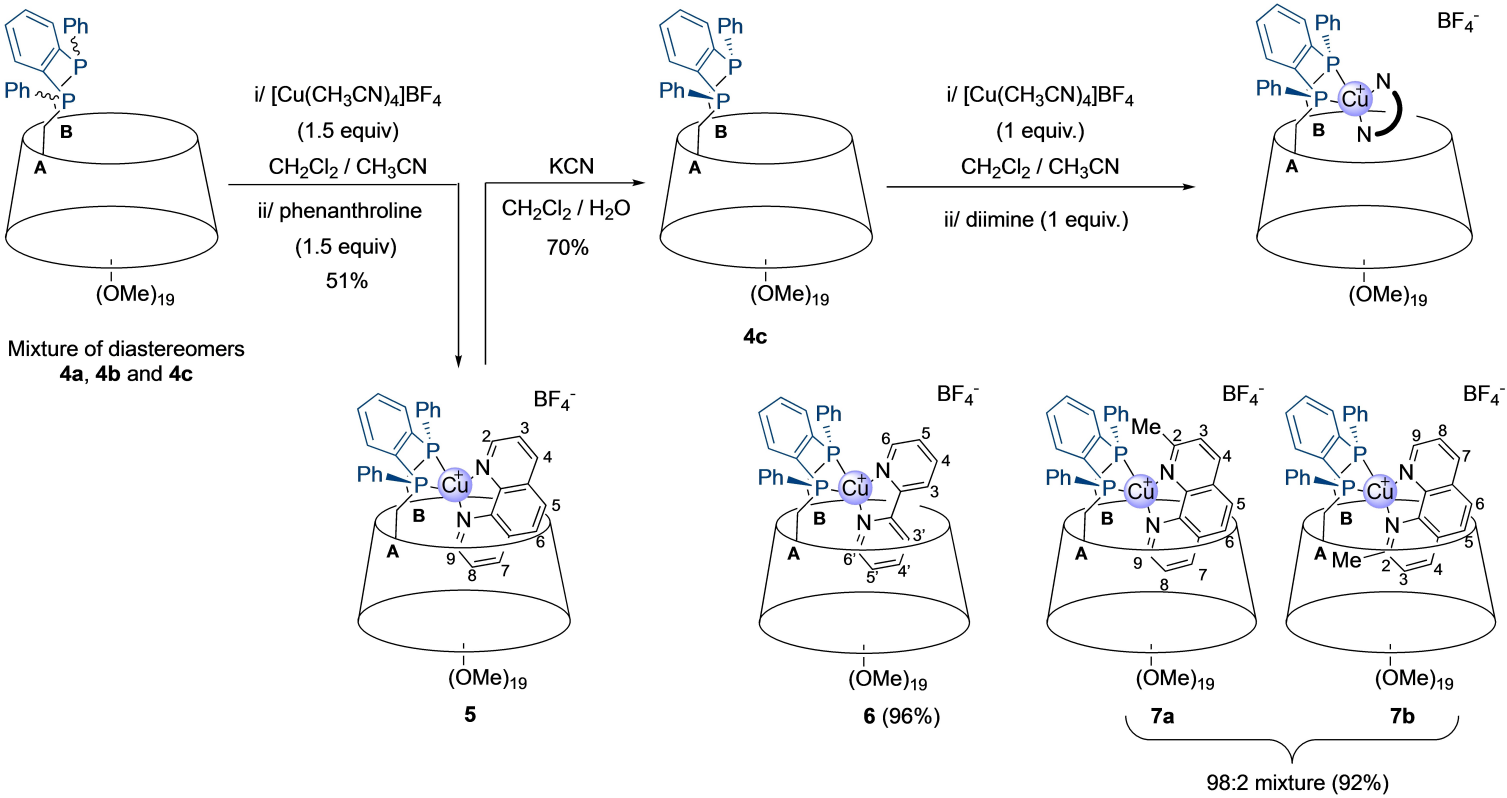
Synthesis of stereopure ligand **4 c** and its heteroleptic [Cu(NN)(PP)]^+^ complexes **5–7**.

While working on the synthesis of new confining CD‐based diphosphanes,^[7]^ we came across an unprecedented Smiles‐like rearrangement of diphenyl‐(2‐phosphanylphenyl)phosphane (**1**) in the presence of an excess of *n*‐butyllithium (Scheme 1). This rearrangement allows the convenient “in situ” quantitative preparation of dilithium 1,2‐bis(phenylphosphanide)benzene as a 1 : 1 mixture of the two diastereomers **2 a,b** from **1** (Figures S1a,b). Previously reported procedures for the synthesis of this bis‐nucleophile are cumbersome and often involve toxic reagents such a phosgene.^[8]^ The mixture **2 a,b** was reacted with β‐CD‐derived dimesylate **3** in THF to afford diphosphane‐capped CDs in 75 % yield as a 6 : 4 mixture consisting of, respectively, the two *trans* isomers **4 a**,**b** and the desired confining *cis* diphosphane **4 c** (Scheme 2). The presence of a second *cis* diastereomer was not detected in the reaction mixture presumably because it would require the inclusion of two phenyl rings in the β‐CD cavity, which is highly unfavorable for steric reasons. Heating the mixture of diphosphanes in boiling mesitylene (160 °C) for 4 h caused a change in the diasteromeric ratio, with diphosphane **4 c** being now the major and thermodynamic compound (6 : 4 molar ratio in favor of **4 c**) (Figures S2a,b) as a result of phosphorus inversion. Remarkably, only the *cis*‐chelating diphosphane **4 c** is capable of forming a stable tetrahedral complex (**5**) with [Cu(CH_3_CN)_4_]BF_4_ and phenanthroline (phen), which can easily be isolated in 51 % yield from the reaction mixture by column chromatography (Scheme 3). The pure ligand **4 c** was then obtained in 70 % yield by demetallation of **5** with potassium cyanide. Ligand **4 c** is remarkably stable in air as no oxidation could be observed during column chromatography.

In stark contrast with their *trans* counterparts **4 a,b**, in which the phosphorus atoms resonate as two singlets at −36.6 and −37.8 ppm (**4 a**) and −38.6 and −3.2 ppm (**4 b**),^[9]^ *cis*‐chelating **4 c** displays an AB system at *δ*=−24.6 and −17.7 ppm with a large ^3^ *J*_P,P_ coupling constant of 168 Hz typical of *syn*‐1,2‐bis(alkylphenylphosphano)benzene derivatives. The free ligand allowed us to synthesize additional heteroleptic [Cu(NN)(PP)]^+^ complexes (**6,7**) involving different NN diimine ligands such as 2,2’‐bipyridine (bpy) and 2‐methyl‐1,10‐phenanthroline (mmp) (Scheme 3). However, the more sterically demanding 2,9‐dimethyl‐1,10‐phenanthroline (dmp) ligand gave a mixture of several complexes including the desired heteroleptic CD containing Cu^I^ complex but also [Cu(dmp)_2_]BF_4_ (Figures S5a–c). All attempts to isolate pure [Cu(**4 c**)(dmp)]BF_4_ failed owing to its instability. Clearly, when the nitrogen coordination sites in the diimine ligand are too sterically hindered, the formation of the targeted heteroleptic [Cu(NN)(PP)]^+^ complex is disfavored thus leading to a dynamic mixture of copper(I) complexes.

**Figure 1:**
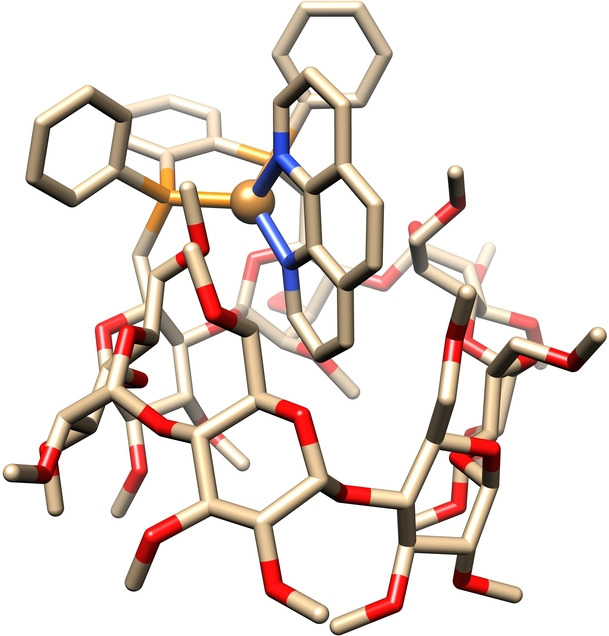
X‐ray crystal structure of complex **8** (PF_6_ counteranion, solvent molecules, and H atoms omitted for clarity; C: pale gray, N: blue, O: red, P: orange, Cu: pale gold).

The heteroleptic nature of complex **5** was confirmed by the exclusive presence of peaks corresponding to the [Cu(NN)(PP)]^+^ cation in the ESI‐TOF spectrum. Typical deshielded signals at *δ*=−4.3 and 2.5 ppm for the P,P′ AB system with a slightly larger *J*_P,P_ coupling constant of 181 Hz are indicative of metal complexation. Proof that the phen ligand is coordinated within the CD cavity came from extensive 2D NMR studies, which allowed us to carry out a full assignment of the ^1^H NMR spectrum of **5**. As expected, signals associated with the phen ligand were doubled upon coordination as a result of the dissymmetry of the CD host. Moreover, the CD H‐6 and MeO‐6 protons of glucose units C and G lie in the phen shielding cone resulting in strongly upfield shifted signals (Figure S4g). However, the most compelling evidence for phen encapsulation came from the detailed analysis of the 2D ROESY spectrum of **5** which shows numerous cross‐peaks corresponding to through‐space correlations between inner cavity CD H‐3 and H‐5 and phen protons (Figure S4g). Correlations between the phen H‐9 and CD H‐5_A,B_ protons on one hand and those involving the phen H‐2 proton and the phenyl ortho protons of the diphosphane unit are indicative of a phen unit sitting perpendicular to the P−Ar‐P plane. Additionally, intense cross‐peaks corresponding to correlations between the phen H‐5, H‐6 and H‐7 protons and intra‐cavity H‐3 and H‐5 protons of CD glucose units D, E and F are indicative of a deep encapsulation of the lower part of the phen ligand. Finally, signals for phen H‐2 and H‐9 protons as well as phosphorus atoms display line broadening compared to other signals, which is indicative of fluxional behavior around the metal center. A VT NMR study performed in CD_2_Cl_2_ (Figures S4j,k) revealed two species in slow exchange at low temperature (−60 °C) in a 2 : 1 ratio with a coalescence temperature of around −20 °C. The most striking feature is the large chemical shift difference (Δ*δ*=1.1 ppm) for the phen H‐9 proton between the two species at −60 °C. A single crystal X‐ray analysis of complex **8** ^[10]^ in which the BF_4_^−^ counterion of **5** has been replaced with PF_6_^−^ was undertaken to have a clearer picture of the fluxional phenomenon at stake. As expected, the coordinated phen is partially encapsulated in the CD host as in solution (Figure 1). However significant departure from an ideal tetrahedral geometry could be revealed. Indeed, the phen ligand is slightly tilted and forms an angle of 80.8° with the P−Ar−P plane. Moreover, the glucosidic oxygen atom linking the A and B units (O‐4_A_) forms a weak hydrogen bond with the phen H‐9 atom (phen H‐9⋅⋅⋅O‐4_A_=2.21 Å). Given the deformation of the metal first sphere of coordination in the solid state, the fluxional phenomenon observed in solution is likely the result of the oscillation of the phen ligand around the axis running through the metal and parallel to both the P−Ar−P and phen planes. This implies a much weaker phen H‐9⋅⋅⋅O‐4_A_ hydrogen bond in one of the conformers, which could explain the very different chemical shifts experienced by the phen H‐9 proton in the two species.

Combined with the rigidity of both diphosphane and phen ligands, the metal chelation induces strong ring strain on CD glucose unit B which adopts the ^O^ *S*_2_ form, an unusual glucose conformer that is only observed in highly strained CD derivatives.^[11]^ The overall CD structure is also strained as it no longer adopts the usual circular shape, but turns out to be near rectangular in order to accommodate the rather bulky phen unit. With a less sterically demanding diimine ligand such as bpy, the protons adjacent to the nitrogen atoms (H‐6 and H‐6′) are again the broadest in complex **6** suggesting the presence of the same fluxional phenomenon as described above. However, a lower coalescence temperature (−40 °C) and a 9 : 1 ratio in favor of the species with the most upfield bpy H‐6′ proton was observed, which points to a faster exchange between the two conformers in equilibrium (Figures S6i,j). As expected, a 98 : 2 mixture of two complexes (**7 a** and **7 b**) is formed in the case of the nonsymmetrical mmp ligand as evidenced by a ^1^H/^13^C{^1^H} HSQC NMR experiment (Figure S7j).

The major one (**7 a**) exhibits a strongly upfield shifted 2‐methyl group (*δ*=2.27 ppm for **7 a** vs. 3.41 ppm for **7 b**, Figure S7j) located outside the cavity and sandwiched between the two phenyl rings of the CD ligand in keeping with ROESY correlations between aromatic diphosphane and the mmp 2‐methyl protons at *δ*=2.27 ppm **(**Figure S7g). The formation of these two species occurs likely under kinetic control since they are not in equilibrium as revealed by a high temperature VT NMR study (Figures S7m,n). The absence of dynamic exchange further highlights the remarkable stability of this new family of Cu^I^ complexes. Again, the oscillation of the mmp ligand in **7 a** produces two conformers in slow exchange at the NMR timescale however in a 9 : 1 ratio (Figure S7k). The conformer with the most upfield shifted H‐9 proton is the major one.

The electrochemical properties of **5** and **6** as well of the inseparable mixture **7 a**,**b** were determined by cyclic voltammetry (CV) and Osteryoung square wave voltammetry (OSWV). Potential data for all of the compounds are collected in Table 1, and the voltammograms are shown in figures S9–S13. All the complexes revealed the typical electrochemical response of [Cu(NN)(PP)]^+^ derivatives and the observed one‐electron reduction process is centered on the phenanthroline or bipyridine moiety (−1.5≤*E*_Red_≤−1.48 V). In the anodic region, the one‐electron oxidation process attributed to the Cu^II^/Cu^I^ redox couple was observed at +1.13 and +1.15 V vs. SCE for respectively **5** and **6**. Oxidation of the Cu^I^ center is more difficult in the case of **7 a**,**b** than for **5** and **6** (Δ*E*_ox_=60 and 40 mV respectively). Unsurprisingly, the 2‐methyl substituent in **7 a**,**b** prevents the formation of a more flattened structure that suits the Cu^II^ oxidation state. The potential shift results therefore from the destabilization of the Cu^II^ complexes as typically observed in the case of homoleptic [Cu(NN)_2_]^+^ and other [Cu(NN)(PP)]^+^ complexes. Moreover, compared to analogous [Cu(phen)(dppe)]^+^ (dppe=1,2‐bis‐(diphenylphosphano)ethane) and [Cu(phen)(dppb)]^+^ (dppb=1,2‐bis‐(diphenylphosphano)benzene) complexes,^[12]^ significant anodic shifts of Δ*E*_ox_=90 and 80 mV, respectively, were observed for **5**, suggesting an unfavorable flattening of the metal coordination sphere upon Cu^I^ oxidation as a result of the encapsulation of the phen moiety in the cavity of the CD macrocycle. Additionally, when compared to some previous heteroleptic Cu^I^ pseudorotaxanes prepared from macrocyclic phenanthroline ligands and bis[2‐(diphenylphosphano)phenyl]ether) (POP),^[6]^ a similar anodic potential shift was evidenced for the metal‐centered oxidation. In this particular case, this shift is more likely due to the increased size of the 2,9‐substituents of the phenanthroline ligands rather than being related to their macrocyclic nature. Furthermore, in the case of the Cu^I^ pseudorotaxanes, the electrochemical studies revealed the presence of small amounts of [Cu(phen)_2_]^+^ resulting from the decoordination of the POP ligand in solution. Such a behavior was not observed for the copper complexes prepared from the CD‐based PP ligand **4 c** thus showing clearly a gain in stability.

**Table 1:** Selected electrochemical data of complexes **5**, **6**, and **7 a,b** in CH_2_Cl_2_ together with the data for appropriate model compounds recorded under the same experimental conditions.

		Electrochemical data^[a]^		
Complex	NN ligand	*E*_ox_	*E*_red_	Δ*E*_redox_
[Cu(phen)(dppe)]^+[b]^	phen	+1.04	−1.53	2.57
[Cu(phen)(dppb)]^+[b]^	phen	+1.05	−1.50	2.55
**5**	phen	+1.13	−1.50	2.63
**6**	bpy	+1.15	−1.48	2.63
**7 a,b**	mmp	+1.19	−1.49	2.68

[a] Selected data refer to OSWV experiments; ferrocene is used as an internal reference (F_c_^+^/F_c_ is observed at 0.55±0.01 V vs. SCE). *E*_ox_=first oxidation potential, *E*_red_=first reduction potential in V vs. SCE; Δ*E*_redox_=*E*_ox_–*E*_red_. [b] From reference [12].

**Figure 2:**
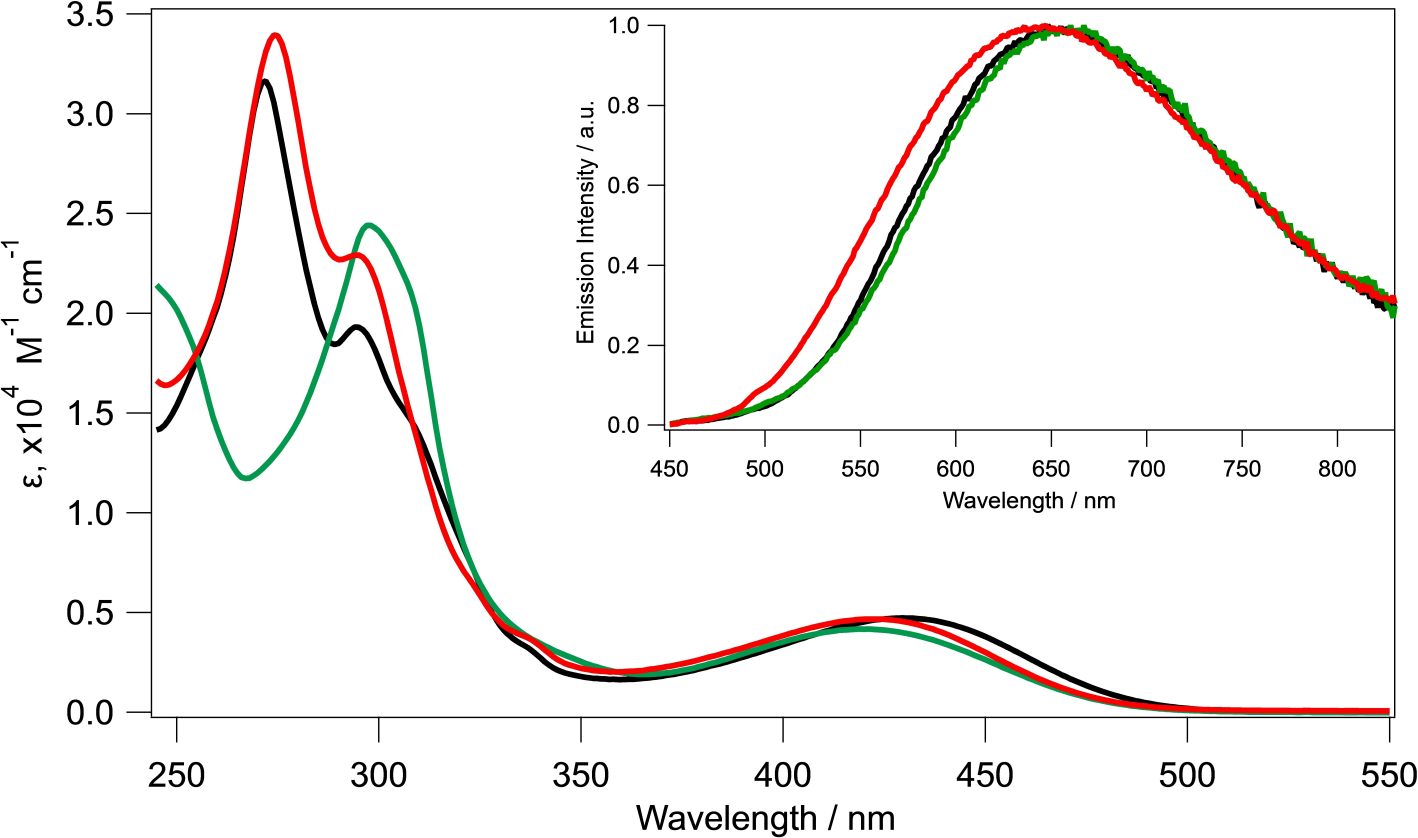
Absorption spectra of **5** (black), **6** (green), and **7 a,b** (red) in CH_2_Cl_2_. Inset: Emission spectra of **5**, **6**, and **7 a,b** in CH_2_Cl_2_ solution at 298 K, λ_exc_=430 nm.

The absorption spectra of complexes **5**–**7 a,b** in CH_2_Cl_2_ are displayed in Figure 2 and related key data are reported in Table S1. The three complexes exhibit intense absorption bands (ϵ >2×10^4^ M^−1^ cm^−1^) in the UV region up to about 310 nm, which are mainly attributable to ligand‐centered (LC) transitions (π→π*) of both the NN and PP ligands.[^6d^, ^12^] The two phenanthroline‐based systems (**5** and **7 a,b**) have very similar spectral shapes in this window, whereas the spectrum of **6** reflects the presence of the different bpy diimine ligand. Additionally, the complexes exhibit a broader and weaker band (ϵ <5×10^3^ M^−1^ cm^−1^) between 360 and 500 nm, assigned to MLCT transitions that involve the Cu^I^ ions and the NN ligands. The absorption spectral shapes and intensities do not vary significantly up to six days in the dark at 298 K. Therefore, these complexes are extremely stable in solution (Figure S14).

The photoluminescence properties of the β‐CD‐based Cu^I^ complexes have been investigated in CH_2_Cl_2_ solution (298 K), rigid CH_2_Cl_2_ matrix (77 K) and in PMMA films (298 K, 1 wt%). The emission spectra are depicted in Figure 2 (inset) and 3, and the related photophysical data are collected in Table 2. The experiments were performed on fresh solutions bubbled with argon for at least 20 min to remove oxygen. At room temperature, the three complexes show the same broad and unstructured emission bands centered around 650 nm, upon excitation at 430 nm (Figure 2, inset). These are clearly attributable to the deactivation from MLCT excited states.[^3a^, ^3d^, ^3f^, ^5a^, ^12^, ^13^] Complexes equipped with phen (**5**) and bpy (**6**) exhibit lower PLQYs compared with the complex containing mmp (**7 a,b**). This behavior is in line with previous studies showing that the lack of sterically active substituents on the diimine ligands enables the structural distortion of the four‐coordinated complex in the excited state, which in turn favors non‐radiative decay of the excited states back to the ground state.[^3a^, ^3d^, ^3f^, ^5a^, ^12^, ^13c^, ^14a^, ^14b^, ^14c^, ^14d^, ^14e^, ^14f^, ^14g^] It is noteworthy that, albeit weak, **5** and **6** exhibit luminescence in solution also in the absence of substituents on the NN ligands, which is typically not the case for [Cu(NN)(PP)]^+^ complexes. The effect is remarkable also in PMMA where the luminescence quantum yield of **5** is 60 % higher than that of the related [Cu(phen)(dppb)]^+^ parent compound. Therefore this trend can be attributed to steric protection of the metal center by the CD cavity.[^3f^, ^12^]

**Table 2:** Photophysical data of complexes **5**–**7 a,b** in CH_2_Cl_2_ at 298 K, in frozen solution at 77 K, and in 1 wt % PMMA at 298 K.

	CH_2_Cl_2_, 298 K			CH_2_Cl_2_, 77 K		PMMA (1 wt %), 298 K		
Complex	λ_em_ [nm]	PLQY [%]^[a]^	τ [ns]^[b]^	λ_em_ [nm]	τ [μs]^[c]^	λ_em_ [nm]	PLQY [%]^[d]^	τ [μs]^[e]^
[Cu(phen)(dppe)]^+[f]^	n.d.^[g]^			≈652	0.16^[h]^	≈612	0.2	2.9^[h]^
[Cu(phen)(dppb)]^+[f]^	n.d.^[g]^			≈615	0.19^[h]^	≈588	2.4	6.8^[h]^
**5**	∼650	0.3	90	≈620	60 330^[i]^	≈580	5.6	13 232^[j]^
**6**	∼650	0.1	119 6.5^[j]^	≈640	48 309^[i]^	≈605	0.8	251
**7 a,b**	∼650	0.5	128	≈580	55 320^[i]^	≈555	7.5	18 258^[k]^

[a] λ_exc_=380 nm. [b] λ_exc_=373 nm, nanoLED. [c] λ_exc_=370 nm, SpectraLED. [d] λ_exc_=380 nm, determined using an integrating sphere. [e] λ_exc_=370 nm, SpectraLED. [f] From reference [12]. [g] n.d.=not detected. [h] Average of biexponential decay. [i] Biexponential decay, with a relative amplitude 10 : 90 %. [j] Biexponential decay, with a relative amplitude 40 : 60 %. [k] Biexponential decay, with a relative amplitude of 45 : 55 %.

Excited‐state lifetimes were best fitted with a monoexponential function for the two complexes containing phen (**5**) and mmp (**7 a,b**) ligands, yielding lifetimes of 90 and 128 ns, respectively. A bi‐exponential decay was instead observed for the bpy containing complex **6** (6.5 and 119 ns). Observed lifetimes tend to increase with the size of the NN ligand and the addition of a methyl group to the phen ligand (**7 a,b**).

**Figure 3:**
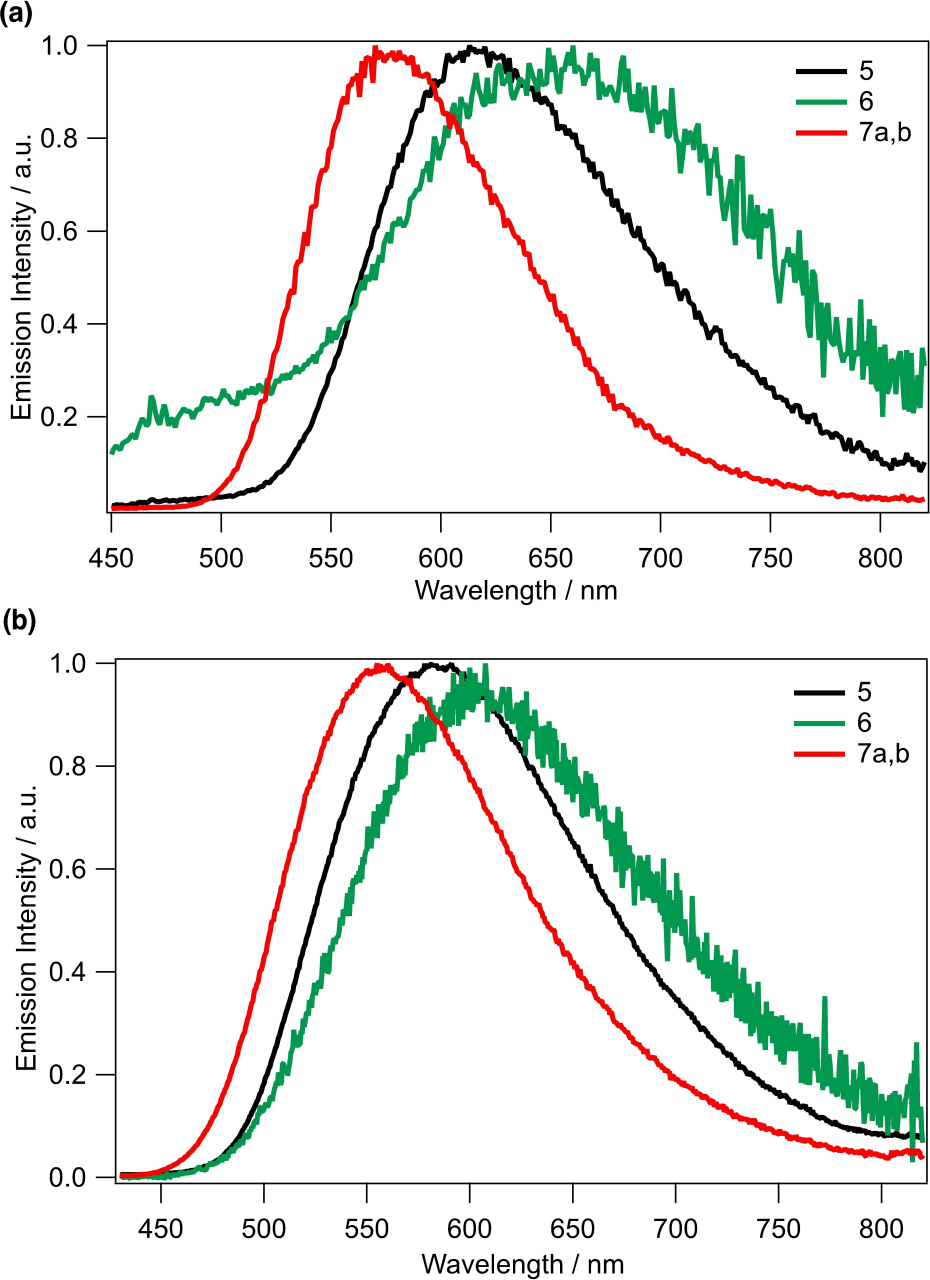
Emission spectra of **5**, **6**, and **7 a,b** (a) in CH_2_Cl_2_ at 77 K, λ_exc_=300 nm; (b) in PMMA 1 wt% at 298 K, λ_exc_=380 nm.

In CH_2_Cl_2_ rigid matrix at 77 K, emission bands are blue‐shifted when compared to those at 298 K (Figure 3a). The shift is more remarkable for the complex with the bulkiest ligand mmp (**7 a,b**, about 70 nm), while that for the phen‐based system is less pronounced (**5**, about 30 nm). The complex equipped with bpy (**6**) has a weaker and much less blue‐shifted band. The emission spectral shifting to higher energy is induced by rigidochromic effects in the glass matrix due to limited geometric relaxation of the excited state.[^6d^, ^12^] The emission decays were best fitted with bi‐exponential functions, yielding very similar lifetimes in the range 48–60 μs for τ_1_, and 309–330 μs for τ_2_ (Table 2).

Luminescence spectra were also recorded in PMMA matrix (Figure 3b). When compared to those observed in CH_2_Cl_2_ solutions at 298 K, the emission bands are blue‐shifted and PLQYs are remarkably higher, again due rigidochromic effects. Such emission blue‐shift is more pronounced in **7 a,b** (nearly 100 nm), which is the system with the bulkiest diimine ligand (mmp) and the highest PLQY (7.5 %). The complex with the smallest diimine ligand (bpy, **6**) shows lower PLQY= 0.8 % and a shift of less than 50 nm. Since Cu^I^ complexes undergo a flattening distortion in the MLCT excited‐state, the steric effects of the ligand have a marked impact on such geometrical relaxations occurring after light excitation.[^3f^, ^13c^, ^15^] As stated above, the lower the excited‐state flattening, the higher the PLQY. Furthermore, it was shown that PLQYs are linked (i) to the bulkiness and/or rigidity of the ligand itself and (ii) the “locking” effect on the complex geometry, provided by inter‐ and intramolecular π‐stacking interactions. Accordingly, a correlation was proposed, i.e., the higher the number of intermolecular π‐interactions, the larger the PLQY.^[12]^ Given the lack of either intra‐ or intermolecular π‐stacking interactions in these rather large complexes, as revealed by the molecular structure of **8**, the PLQY must be solely influenced by ligand sterics. Emission decays of the phenanthroline‐based systems in PMMA are best fitted by bi‐exponential functions (Table 2), which may reflect the presence of closely spaced excited states. The longer lifetime of **7 a,b** is the longest of the series, underpinning peculiar steric effects appearing also in PMMA rigid matrix. Notably, the lifetimes in PMMA of the present CD‐based series is at least one order of magnitude longer than those typically observed in other [Cu(NN)(PP)]^+^ systems, suggesting again a remarkable role played by CD steric effects^.[3f, 9]^ Clearly, encapsulation of the photoactive metal center has a marked influence on the emission and excited state properties of the Cu^I^ complexes, particularly at room temperature. In CH_2_Cl_2_ solution, some luminescence is observed also with complexes bearing pristine phenanthroline or even bpy, an unprecedented finding, while these compounds have a remarkable luminescence efficiency in PMMA, with record long excited state lifetimes.

## Conclusion

In summary, a previously unknown Smiles‐type rearrangement of diphosphane **1** allowed us to access the first metal‐confining and *cis*‐chelating diphosphane (**4 c**). Such a ligand was shown to promote the formation of stable heteroleptic [Cu(NN)(PP)]^+^ complexes displaying enhanced light‐induced properties compared to analogues lacking the cavity‐shaped ligand. These findings augur well for the use of tailored CD‐based Cu^I^ systems as noble‐metal free photocatalysts, including in aqueous media.

## Conflict of interest

The authors declare no conflict of interest.

1

## Supporting information

As a service to our authors and readers, this journal provides supporting information supplied by the authors. Such materials are peer reviewed and may be re‐organized for online delivery, but are not copy‐edited or typeset. Technical support issues arising from supporting information (other than missing files) should be addressed to the authors.

Supporting Information

Supporting Information

## Data Availability

The data that support the findings of this study are available in the supplementary material of this article.
