# Supplementary material for: Stable Luminescent [Cu(NN)(PP)]+ Complexes Incorporating a β‐Cyclodextrin‐Based Diphosphane Ligand with Metal‐Confining Properties
Source: Angew Chem Int Ed Engl. 2022 Dec 16;62(6):e202214638. doi: 10.1002/anie.202214638 (PMC10107221; doi:10.1002/anie.202214638)
Supplement: Supplementary file 1 — Supporting Information [file ANIE-62-0-s001.pdf]

Angewandte Chemie
Eine Zeitschrift der Gesellschaft Deutscher Chemiker

Supporting Information

Stable Luminescent $[Cu(NN)(PP)]^+$ Complexes Incorporating a β-Cyclodextrin-Based Diphosphane Ligand with Metal-Confining Properties

T.-A. Phan, N. Armaroli, A. Saavedra Moncada, E. Bandini, B. Delavaux-Nicot*, J.-F. Nierengarten*, D. Armspach**

Supporting Information

Stable Luminescent $[Cu(NN)(PP)]^+$ Complexes Incorporating a β-Cyclodextrin-Based Diphosphane Ligand with Metal-Confining Properties

Tuan-Anh Phan, Nicola Armaroli, Alejandra Saavedra Moncada, Elisa Bandini, Béatrice Delavaux-Nicot*, Jean-François Nierengarten* and Dominique Armspach**

Table of Contents

Synthesis	S3
NMR and mass spectra	S14
Electrochemical measurements	S75
Photophysical measurements	S88
X-ray crystal structure	S90
References	S91

Synthesis

General methods. All manipulations were performed in Schlenk-type flasks under dry nitrogen. Solvents were dried by conventional methods and distilled immediately prior to use. Deuterated solvents were passed down a 5 cm-thick alumina column and stored under nitrogen over molecular sieves (4 Å). Routine ^{1}H and $^{13}C\{^{1}H\}$ spectra were recorded on FT Bruker AVANCE 300, AVANCE 400 and AVANCE 500 at room temperature unless otherwise stated. ^{1}H NMR spectral data were referenced to residual protiated solvents (δ = 5.32 ppm for CD_2Cl_2), $^{13}C\{^{1}H\}$ chemical shifts are reported relative to deuterated solvents (δ = 53.84 ppm for CD_2Cl_2) and the $^{31}P\{^{1}H\}$ NMR data are given relative to external H_3PO_4. Mass spectra were recorded either on a Bruker MicroTOF spectrometer (ESI-TOF) using CH_2Cl_2, CH_3CN or CH_3OH as the solvent. Elemental analyses were performed by the Service de Microanalyse, Institut de Chimie, Strasbourg. All commercial reagents were used as supplied. Dimesylate **3**,[1] 2-methyl-1,10-phenanthroline[2], diphenyl-(2-phosphanylphenyl)phosphane[3] and $[Cu(CH_3CN)_4]BF_4$[4] were prepared according to literature procedures. In this publication, the cyclodextrins are depicted as seen from the secondary face, the glucose units being ranged counterclockwise in the following order: A, B, C, D, E, F, G. The numbering of the atoms within a glucose unit is as follows:

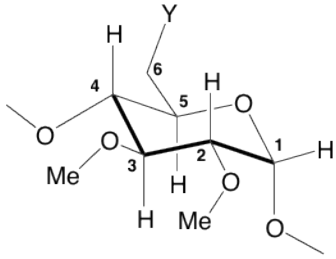

$6^A,6^B$-Dideoxy-$6^A,6^B$-[(1*R*,2*S*)-1,2-phenylene-bis(phenylphosphanyl)]-$2^A,2^B,2^C,2^D,2^E,2^F,2^G,3^A,3^B,3^C,3^D,3^E,3^F,3^G,6^C,6^D,6^E,6^F,6^G$-nonadeca-*O*-methyl-β-cyclodextrin (4c)

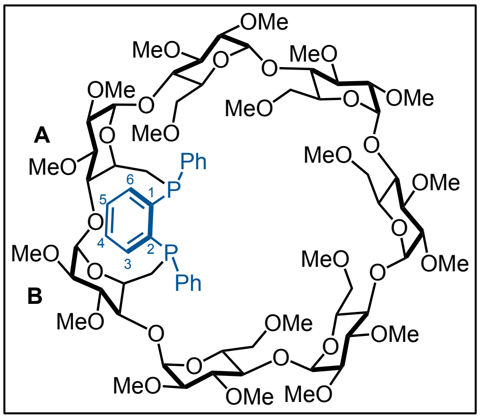

Step 1: A solution of *n*-BuLi in hexane (1.6 M, 0.73 mL, 1.16 mmol) was added dropwise to a stirred solution of diphenyl-(2-phosphanylphenyl)phosphane (0.085 g, 0.29 mmol) in thf (6 mL) at –78°C. After 10 min, the reaction mixture was allowed to reach room temperature and then kept at this temperature for 8 h under stirring. The resulting red suspension was cannulated within 15 min into a stirred solution of dimesylate **3** (0.30 g, 0.19 mmol) in thf (12 mL). The reaction mixture was stirred for 12 h at room temperature. The solvent was then removed *in vacuo* and excess Li_2PAr was protonated with MeOH (5 mL). After removal of the solvent *in vacuo*, mesitylene (20 mL) was added and the resulting solution was heated at reflux for 4 h. Removal of the solvent *in vacuo* afforded a colourless solid, which was filtered over a short plug of silica using CH_2Cl_2/MeOH (500 mL, 90:10, *v*/*v*) as solvent. The resulting colourless residue was then subjected to column chromatography (SiO_2, CH_2Cl_2/MeOH, 97:3, *v*/*v*) to afford **4c** and two of its diastereomers **4a**, **4b** as a 60/40 mixture (yield: 0.239 g, 75%).

R_f (SiO_2, CH_2Cl_2/MeOH, 95:5, *v*/*v*) = 0.40.

Step 2: $[Cu(CH_3CN)_4]BF_4$ (31 mg, 0.098 mmol) was added to a solution of **4a**, **4b** and **4c** (120 mg, 0.072 mmol) in CH_2Cl_2/CH_3CN (10 mL, 7:3, *v*/*v*) at room temperature. The orange solution was stirred for 0.5 h before adding 1,10-phenanthroline (18 mg, 0.098 mmol). After removal of the solvent *in vacuo*, the residue was subjected to column chromatography (SiO_2, CH_2Cl_2/MeOH, 95:5, *v*/*v*) to afford pure **5** (yield: 74 mg, 51%) as a yellow solid.

R_f (SiO_2, CH_2Cl_2/MeOH, 95:5, *v*/*v*) = 0.28.

Step 3: KCN (0.245 g, 3.77 mmol) in water (5 mL) was added to **5** (0.15 g, 0.075 mmol) in CH_2Cl_2 (5 mL). After 2 h stirring at room temperature, the yellow organic phase turned colourless. The mixture was extracted with CH_2Cl_2 (3 x 10 mL) and the combined organic layers were dried ($MgSO_4$), filtered and evaporated. The residue was then subjected to column chromatography (SiO_2, CH_2Cl_2/MeOH, 95:5, *v*/*v*) to afford ligand **4c** (yield: 0.088 g, 70%) as a colourless solid.

R_f (SiO_2, CH_2Cl_2/MeOH, 95:5, *v*/*v*) = 0.34.

^1H NMR (500 MHz, CD_2Cl_2, 25°C): δ (assignment by combined COSY, ROESY, TOCSY and HSQC) = 2.33 (m, 1H, H-6a^A), 2.53 (m, 1H, H-6a^B), 2.77 (s, 3H, CH_3O-6), 3.03 (m, 1H, H-6b^B), 3.07 (s, 3H, CH_3O-6), 3.28 (s, 3H, CH_3O-6), 3.32 (s, 3H, CH_3O-6), 3.35 (s, 3H, CH_3O-6), 3.41 (s, 3H, CH_3O-2), 3.43 (s, 3H, CH_3O-2), 3.47 (s, 3H, CH_3O-2), 3.49 (s, 3H, CH_3O-2), 3.49 (s, 3H, CH_3O-2), 3.50 (s, 3H, CH_3O-2), 3.52 (s, 3H, CH_3O-2), 3.54 (s, 3H, CH_3O-3), 3.59 (s, 3H, CH_3O-3), 3.61 (s, 6H, CH_3O-3), 3.62 (s, 3H, CH_3O-3), 3.63 (s, 3H, CH_3O-3), 3.66 (s, 3H, CH_3O-3), 3.04-3.96 (37H, H-2, H-3, H-4, H-5^{C,D,E,F,G}, H-6^{C,D,E,F,G}, H-6b^A), 4.40 (m, 1H, H-5^A), 4.41 (m, 1H, H-5^B), 4.85 (d, $^3J_{H\text{-}2,H\text{-}1}$ = 3.6 Hz, 1H, H-1^B), 4.97 (d, $^3J_{H\text{-}2,H\text{-}1}$ = 3.3 Hz, 1H, H-1), 5.00 (d, $^3J_{H\text{-}2,H\text{-}1}$ = 3.4 Hz, 1H, H-1^A), 5.06 (d, $^3J_{H\text{-}2,H\text{-}1}$ = 3.5 Hz, 1H, H-1), 5.19 (d, $^3J_{H\text{-}2,H\text{-}1}$ = 3.7 Hz, 1H, H-1), 5.21 (d, $^3J_{H\text{-}2,H\text{-}1}$ = 3.8 Hz, 1H, H-1), 5.28 (d, $^3J_{H\text{-}2,H\text{-}1}$ = 4.0 Hz, 1H, H-1), 6.62 (m, 1H, H-3$_{arom}$), 7.05 (m, 1H, H-4$_{arom}$), 7.17 (m, 1H, H-5$_{arom}$), 7.21-7.41 (6H, $H_{orthoPh}$, H_{metaPh}, H_{paraPh}), 7.43 (m, 2H, H_{metaPh}), 7.49 (m, 1H, H-6$_{arom}$), 7.62 (m, 2H, $H_{orthoPh}$) ppm; $^{13}C\{^1H\}$ NMR (126 MHz, CD_2Cl_2, 25°C): δ (assignment by HSQC) = 31.19 (dd, $^1J_{P,C}$ = 16.8 Hz, $^4J_{P,C}$ = 4.9 Hz, C-6^B), 32.81 (d, $^1J_{P,C}$ = 13.8 Hz, C-6^A), 57.76, 57.90, 58.22, 58.53, 58.75, 59.09, 59.11, 59.13, 59.14, 59.20, 59.53 (d) and 59.76 (CH_3O-2 and CH_3O-6), 60.89, 61.41, 61.57, 61.68, 61.76, 61.87 and 62.10 (CH_3O-3), 70.69, 71.07, 71.37, 71.64 and 71.73 (C-5^{C,D,E,F,G}), 71.16, 71.59, 71.62, 71.75 and 71.80 (C-6^{C,D,E,F,G}), 73.00 (d, $^2J_{P,C}$ = 19.7 Hz, C-5^B), 73.18 (d, $^2J_{P,C}$ = 14.2 Hz, C-5^A), 82.46 (d, $^4J_{P,C}$ = 1.5 Hz, C-3^A), 82.93 (d, $^4J_{P,C}$ = 3.5 Hz, C-3^B), 76.53, 79.08, 80.03, 80.47, 80.98, 81.47, 81.49, 81.97, 81.99, 82.02, 82.04, 82.17, 82.18, 82.45, 82.46, 82.48, 82.60, 83.00 and 83.13 (C-2, C-3^{C,D,E,F,G}, C-4^{C,D,E,F,G}), 86.30 (d, $^3J_{P,C}$ = 11.2 Hz, C-4^B), 89.89 (d, $^3J_{P,C}$ = 9.8 Hz, C-4^A), 98.44, 98.46, 98.65, 98.81, 98.90, 99.55 and 100.07 (C-1), 127.75 (s, C_{paraPh}), 128.36 (s, C-5$_{arom}$), 128.57 (d, $^3J_{P,C}$ = 5.4 Hz, C_{metaPh}), 128.81 (d,

$^{3}J_{P,C}$ = 6.1 Hz, C_{metaPh}), 128.96 (s, C_{paraPh}), 129.09 (s, C-4$_{arom}$), 130.62 (d, $^{3}J_{P,C}$ = 8.8 Hz, C-3$_{arom}$), 131.47 (d, $^{2}J_{P,C}$ = 16.7 Hz, $C_{orthoPh}$), 134.00 (d, $^{2}J_{P,C}$ = 20.2 Hz, $C_{orthoPh}$), 134.77 (dd, $^{3}J_{P,C}$ = 3.5 Hz, $^{4}J_{P,C}$ = 3.3 Hz, C-6$_{arom}$), 137.58 (dd, $^{1}J_{P,C}$ = 18.6 Hz, $^{4}J_{P,C}$ = 15.7 Hz, C_{ipsoPh}), 142.26 (dd, $^{1}J_{P,C}$ = 13.4 Hz, $^{4}J_{P,C}$ = 4.3 Hz, C_{ipsoPh}), 143.68 (dd, $^{1}J_{P,C}$ = 27.7 Hz, $^{2}J_{P,C}$ = 13.2 Hz, C-1$_{arom}$), 151.24 (dd, $^{1}J_{P,C}$ = 34.1 Hz, $^{2}J_{P,C}$ = 13.7 Hz, C-2$_{arom}$) ppm;

$^{31}P\{^{1}H\}$ NMR (121.5 MHz, CD_2Cl_2, 25°C): δ = –24.6 (d, $^{3}J_{P,P}$ = 168 Hz, P_2), –17.7 (d, $^{3}J_{P,P}$ = 168 Hz, P_1) ppm;

elemental analysis (%) calcd for $C_{79}H_{120}O_{33}P_2$.1/6CH_2Cl_2: C 56.81, H 7.25, found: C 56.84, H 7.27;

MS (ESI-TOF) for $C_{79}H_{120}O_{33}P_2$: *m/z* (%): 1697.73 (100) [*M* + O +Na]$^+$, 1713.70 (54) [*M* + 2O +Na]$^+$.

Full ^{1}H NMR assignement of all glucose units of compound **4c** was achieved by combined COSY, ROESY, TOCSY and HSQC (δ values in ppm).

	H-1	H-2	H-3	H-4	H-5	H-6a	H-6b
**Unit A**	5.00	3.10	3.54	3.20	4.40	2.33	3.23
**Unit B**	4.85	3.15	3.49	3.31	4.41	2.53	3.03
**Unit C**	5.21	3.20	3.50	3.62	3.83	3.20	3.58
**Unit D**	5.19	3.10	3.43	3.62	3.63	3.35	3.84
**Unit E**	5.06	3.09	3.44	3.65	3.60	3.41	3.84
**Unit F**	4.97	3.10	3.51	3.57	3.76	3.09	3.72
**Unit G**	5.28	3.06	3.43	3.48	3.67	3.40	3.92

Complex 5 ([Cu(phen)(**4c**)]BF_4)

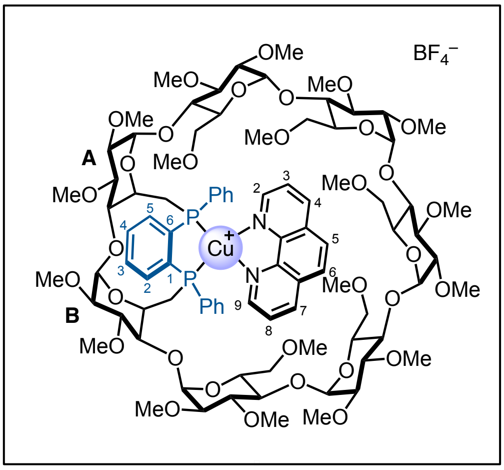

Complex **5** was synthesized according to the procedure outlined above.

^{1}H NMR (500 MHz, CD_2Cl_2, 25°C): δ (assignment by combined COSY, ROESY, TOCSY and HSQC) = 1.10 (s, 3H, CH_3O), 1.54 (s, 3H, CH_3O), 1.72-2.25 (4H, H-6^{C,G}), 2.36 (m, 1H, H-5^{G}), 2.63 (m, 1H, H-6a^{A}), 2.64 (m, 1H, H-6a^{B}), 2.66 (m, 1H, H-6a^{A}), 2.91 (s, 3H, CH_3O), 3.22 (s, 3H, CH_3O), 3.32 (s, 3H, CH_3O), 3.43 (s, 3H, CH_3O), 3.49 (s, 6H, CH_3O), 3.50 (s, 3H, CH_3O), 3.52 (s, 3H, CH_3O), 3.55 (s, 3H, CH_3O), 3.56 (s, 3H, CH_3O), 3.58 (s, 3H, CH_3O), 3.59 (s, 3H, CH_3O), 3.60 (s, 6H, CH_3O), 3.68 (s, 6H, CH_3O), 3.81 (s, 3H, CH_3O), 2.87-3.86 (30H, H-2, H-3, H-4, H-5^{C,D,F}, H-6^{D,F}, H-6a^{E}, H-6b^{B}), 4.03 (m, 1H, H-6b^{E}), 4.22 (m, 1H, H-5^{E}), 4.26 (m, 1H, H-5^{A}), 4.51 (m, 1H, H-5^{B}), 4.62 (d, $^3J_{H-2,H-1}$ = 3.2 Hz, 1H, H-1), 4.91 (d, $^3J_{H-2,H-1}$ = 3.8 Hz, 1H, H-1^{B}), 5.03 (d, $^3J_{H-2,H-1}$ = 3.1 Hz, 2H, H-1), 5.06 (br d, 1H, H-1), 5.22 (d, $^3J_{H-2,H-1}$ = 3.7 Hz, 1H, H-1), 5.32 (br d, 1H, H-1^{A}), 7.17-7.44 (9H, $H_{orthoPh}$, H_{metaPh}, H_{paraPh}, H-3$_{arom}$), 7.57 (m, 1H, H-4$_{arom}$), 7.58 (m, 1H, H-3$_{Phen}$), 7.65 (m, 1H, H-5$_{arom}$), 7.67 (m, 2H, $H_{orthoPh}$), 7.74 (m, 1H, H-6$_{arom}$), 7.97 (d, $^3J_{H-6,H-5}$ = 8.9 Hz, 1H, H-5$_{Phen}$), 8.03 (dd, $^3J_{H-7,H-8}$ = 7.9 Hz, $^3J_{H-9,H-8}$ = 4.9 Hz, 1H, H-8$_{Phen}$), 8.10 (d, $^3J_{H-5,H-6}$ = 8.9 Hz, 1H, H-6$_{Phen}$), 8.38 (br d, 1H, H-2$_{Phen}$), 8.40 (d, $^3J_{H-3,H-4}$ = 8.1 Hz, 1H, H-4$_{Phen}$), 8.72 (d, $^3J_{H-8,H-7}$ = 8.0 Hz, 1H, H-7$_{Phen}$), 9.97 (br d, 1H, H-9$_{Phen}$) ppm;

^{13}C{^{1}H} NMR (126 MHz, CD_2Cl_2, 25°C): δ (assignment by HSQC) = 33.58 and 33.69 (br d, C-6^{A,B}), 57.34, 57.41, 58.06, 58.24, 58.59, 58.63 [x2], 58.76, 59.09, 59.12, 59.47 and 59.54 (CH_3O-2 and CH_3O-6), 60.54, 60.73 [x2], 60.77, 61.25, 61.61 and 61.86 (CH_3O-3), 69.96, 71.25, 71.51, 72.30 and 73.90 (C-5^{C,D,E,F,G}), 70.39, 70.64, 71.65, 72.79 and 73.01 (C-6^{C,D,E,F,G}), 72.08 (br d, C-5^{A}), 75.16 (br d, $^2J_{P,C}$ = 7.4 Hz, C-5^{B}), 78.50, 80.04, 80.07, 80.26, 80.82, 81.10, 81.17, 81.79 [x2], 82.24, 82.43, 82.45 [x2], 82.55, 82.56, 82.58 [x2], 82.92 and 83.05 [x2] (C-2, C-3, C-4^{B,C,D,E,F,G}), 88.62 (d, $^3J_{P,C}$ = 11.6 Hz, C-4^{A}), 98.38 [x2], 98.83, 99.11, 99.49, 100.12 and 101.81 (C-1), 125.02 (s, C-8$_{Phen}$), 125.12 (s, C-3$_{Phen}$), 127.12 (s, C-5$_{Phen}$), 127.91 (s, C-6$_{Phen}$), 129.14 (d, $^3J_{P,C}$ = 8.2 Hz, C_{metaPh}), 129.19 (s, C_{paraPh}), 129.35 (d, $^3J_{P,C}$ = 8.5 Hz, C_{metaPh}), 129.70 (s, $C_{quatPhen}$), 130.00 (s, $C_{quatPhen}$), 130.07 (s, C_{paraPh}), 130.86 (d, $^2J_{P,C}$ = 12.3 Hz, $C_{orthoPh}$), 131.83 (m, C-5$_{arom}$), 132.09 (m, C-4$_{arom}$), 132.12 (d, $^2J_{P,C}$ = 13.4 Hz, $C_{orthoPh}$), 132.66 (d, $^2J_{P,C}$ = 8.5 Hz, C-3$_{arom}$), 133.85 (dd, $^1J_{P,C}$ = 21.0 Hz, $^4J_{P,C}$ = 15.7 Hz, C_{ipsoPh}), 134.25 (d, $^2J_{P,C}$ = 7.7 Hz, C-6$_{arom}$), 134.95 (br dd, C_{ipsoPh}), 137.48 (s, C-4$_{Phen}$), 137.63 (s,

C-7$_{Phen}$), 141.39 (br dd, C-1$_{arom}$), 144.28 (s, C$_{quatPhen}$), 145.37 (s, C$_{quatPhen}$), 147.24 (dd, $^{1}J_{P,C}$ = 41.0 Hz, $^{2}J_{P,C}$ = 33.6 Hz, C-2$_{arom}$), 149.54 (s, C-2$_{Phen}$), 151.46 (s, C-9$_{Phen}$) ppm; $^{31}P\{^{1}H\}$ NMR (202.5 MHz, CD_2Cl_2, 25°C): δ = –4.3 (d, $^{2}J_{P,P}$ = 181 Hz, P$_2$), 2.5 (d, $^{2}J_{P,P}$ = 181 Hz, P$_1$) ppm;

elemental analysis (%) calcd for $C_{91}H_{128}O_{33}P_2N_2CuBF_4.1/3CH_2Cl_2$: C 54.34, H 6.43, N 1.39, found: C 54.36, H 6.57, N 1.38;

MS (ESI-TOF) for $C_{91}H_{128}O_{33}P_2N_2CuBF_4$: *m/z* (%): 1901.72 (100) [*M* – BF_4]$^+$.

Full ^{1}H NMR assignement of all glucose units of compound **5** was achieved by combined COSY, ROESY, TOCSY and HSQC (δ values in ppm).

	H-1	H-2	H-3	H-4	H-5	H-6a	H-6b
**Unit A**	5.32	3.22	3.52	3.29	4.26	2.63	2.66
**Unit B**	4.91	3.50	3.78	3.49	4.51	2.64	3.63
**Unit C**	5.03	3.13	3.45	3.26	3.15	1.95	2.15
**Unit D**	5.06	3.08	3.42	3.52	3.16	3.27	3.61
**Unit E**	5.22	3.45	3.78	3.68	4.22	3.82	4.03
**Unit F**	5.03	3.11	3.44	3.44	3.42	2.96	3.13
**Unit G**	4.62	2.90	3.31	2.93	2.36	1.77	2.22

Complex 6 ([Cu(bpy)(**4c**)]BF_4)

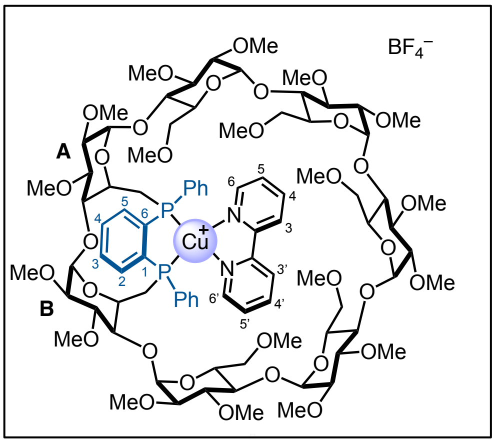

[Cu(CH_3CN)$_4$]BF_4 (8.5 mg, 0.027 mmol) was added to a solution of **4c** (45 mg, 0.027 mmol) in a mixture of CH_2Cl_2/CH_3CN (5 mL, 7:3, *v*/*v*) at room temperature. The resulting solution was stirred for 0.5 h whereupon 2,2'-bipyridine (4.2 mg, 0.027 mmol)

was added. After 2 h, the solvents were removed *in vacuo* and recrystallization of the residue by slow diffusion of Et_2O into a CH_2Cl_2 solution of the crude product gave **6** (yield: 51 mg, 96%) as a yellow crystalline solid.

^{1}H NMR (500 MHz, CD_2Cl_2, 25°C): δ (assignment by combined COSY, ROESY, TOCSY and HSQC) = 1.68 (s, 3H, CH_3O), 2.12 (s, 3H, CH_3O), 2.05 (m, 1H, H-6a^G), 2.39-2.65 (6H, H-5^G, H-6^{A,C}, H-6a^B), 2.78 (m, 1H, H-6b^G), 3.00 (s, 3H, CH_3O), 3.30 (s, 3H, CH_3O), 3.37 (s, 3H, CH_3O), 3.45 (s, 3H, CH_3O), 3.48 (s, 3H, CH_3O), 3.50 (s, 6H, CH_3O), 3.51 (s, 3H, CH_3O), 3.51 (s, 3H, CH_3O), 3.53 (s, 3H, CH_3O), 3.55 (s, 3H, CH_3O), 3.55 (s, 3H, CH_3O), 3.59 (s, 3H, CH_3O), 3.65 (s, 6H, CH_3O), 3.71 (s, 3H, CH_3O), 3.77 (s, 3H, CH_3O), 2.91-3.87 (30H, H-2, H-3, H-4, H-5^{C,D,E}, H-6^{D,E}, H-6a^F, H-6b^B), 4.03 (m, 1H, H-5^F), 4.21 (m, 1H, H-5^A), 4.26 (m, 1H, H-6b^F), 4.36 (m, 1H, H-5^B), 4.75 (d, $^3J_{H\text{-}2,H\text{-}1}$ = 3.4 Hz, 1H, H-1), 4.85 (d, $^3J_{H\text{-}2,H\text{-}1}$ = 3.5 Hz, 1H, H-1^B), 5.00 (d, $^3J_{H\text{-}2,H\text{-}1}$ = 3.4 Hz, 1H, H-1), 5.07 (d, $^3J_{H\text{-}2,H\text{-}1}$ = 3.5 Hz, 1H, H-1), 5.14 (d, $^3J_{H\text{-}2,H\text{-}1}$ = 3.4 Hz, 1H, H-1), 5.16 (d, $^3J_{H\text{-}2,H\text{-}1}$ = 3.7 Hz, 1H, H-1), 5.18 (d, $^3J_{H\text{-}2,H\text{-}1}$ = 4.1 Hz, 1H, H-1^A), 7.18-7.47 (10H, $H_{orthoPh}$, H_{metaPh}, H_{paraPh}, H-3$_{arom}$, H-5$_{Bpy}$), 7.52 (m, 1H, H-4$_{arom}$), 7.59 (m, 1H, H-5$_{arom}$), 7.65 (m, 1H, H-6$_{arom}$), 7.68 (m, 2H, $H_{orthoPh}$), 7.80 (m, 1H, H-5'$_{Bpy}$), 7.88 (m, 1H, H-4$_{Bpy}$), 8.15 (m, 1H, H-4'$_{Bpy}$), 8.19 (d, $^3J_{H\text{-}5,H\text{-}6}$ = 3.6 Hz, 1H, H-6$_{Bpy}$), 8.31 (d, $^3J_{H\text{-}4,H\text{-}3}$ = 8.2 Hz, 1H, H-3$_{Bpy}$), 8.41 (d, $^3J_{H\text{-}4',H\text{-}3'}$ = 8.1 Hz, 1H, H-3'$_{Bpy}$), 9.47 (br d, 1H, H-6'$_{Bpy}$) ppm;

^{13}C{^{1}H} NMR (126 MHz, CD_2Cl_2, 25°C): δ (assignment by HSQC) = 32.31 (br d, C-6^B), 33.03 (d, $^1J_{P,C}$ = 16.6 Hz, C-6^A), 57.73, 58.00, 58.23, 58.40, 58.74, 58.83, 58.93, 59.03 [x2], 59.14, 59.80, 60.00 (CH_3O-2 and CH_3O-6), 60.57, 60.69, 60.86, 61.34, 61.42, 61.52 and 62.17 (CH_3O-3), 70.17, 71.02, 71.56, 71.74 and 73.67 (C-5^{C,D,E,F,G}), 70.75, 71.15, 71.21, 73.19 and 73.42 (C-6^{C,D,E,F,G}), 72.35 (d, $^2J_{P,C}$ = 15.8 Hz, C-5^A), 74.88 (d, $^2J_{P,C}$ = 10.5 Hz, C-5^B), 77.12, 80.07, 80.26, 80.58, 81.04, 81.50 [x2], 81.64, 81.77 [x2], 82.03, 82.12, 82.28, 82.35, 82.51, 82.56, 82.64, 82.82 and 82.84 (C-2, C-3, C-4^{C,D,E,F,G}), 87.06 (br d, C-4^B), 88.48 (d, $^3J_{P,C}$ = 11.8 Hz, C-4^A), 98.44, 98.77, 98.95, 99.18, 99.91, 100.19 and 101.57 (C-1), 122.14 (s, C-3$_{Bpy}$), 122.74 (s, C-3'$_{Bpy}$), 126.01 (s, C-5$_{Bpy}$), 126.17 (s, C-5'$_{Bpy}$), 129.14 (d, $^3J_{P,C}$ = 7.9 Hz, C_{metaPh}), 129.28 (s, C_{paraPh}), 129.37 (d, $^3J_{P,C}$ = 8.4 Hz, C_{metaPh}), 130.23 (s, C_{paraPh}), 130.92 (d, $^2J_{P,C}$ = 11.9 Hz, $C_{orthoPh}$), 131.70 (d, $^3J_{P,C}$ = 2.4 Hz, C-5$_{arom}$), 131.99 (br d, C-4$_{arom}$), 132.31 (d, $^2J_{P,C}$ = 13.9 Hz, $C_{orthoPh}$), 132.41 (d, $^2J_{P,C}$ = 9.7 Hz, C-3$_{arom}$), 133.67 (dd, $^1J_{P,C}$ = 20.7 Hz, $^3J_{P,C}$

= 16.0 Hz, C_{ipsoPh}), 134.09 (d, $^2J_{P,C}$ = 7.5 Hz, C-6$_{arom}$), 134.62 (br dd, C_{ipsoPh}), 138.52 (s, C-4'$_{Bpy}$), 138.61 (s, C-4$_{Bpy}$), 141.30 (dd, $^1J_{P,C}$ = 37.2 Hz, $^2J_{P,C}$ = 28.3 Hz, C-1$_{arom}$), 147.26 (dd, $^1J_{P,C}$ = 40.8 Hz, $^2J_{P,C}$ = 33.1 Hz, C-2$_{arom}$), 149.53 (s, C-6$_{Bpy}$), 150.78 (s, C-6'$_{Bpy}$), 152.64 (s, $C_{quatBpy}$), 154.00 (s, $C_{quatBpy}$) ppm;

$^{31}P\{^1H\}$ NMR (121.5 MHz, CD_2Cl_2, 25°C): δ = –5.2 (d, $^2J_{P,P}$ = 194 Hz, P_2), 2.1 (d, $^2J_{P,P}$ = 194 Hz, P_1) ppm;

elemental analysis (%) calcd for $C_{89}H_{128}O_{33}P_2N_2CuBF_4$.1/2$CH_2Cl_2$: C 53.52, H 6.47, N 1.39, found: C 53.18, H 6.60, N 1.21;

MS (ESI-TOF) for $C_{89}H_{128}O_{33}P_2N_2CuBF_4$: *m/z* (%): 1877.72 (100) $[M - BF_4]^+$.

Full ^{1}H NMR assignement of all glucose units of compound **6** was achieved by combined COSY, ROESY, TOCSY and HSQC (δ values in ppm).

	H-1	H-2	H-3	H-4	H-5	H-6a	H-6b
**Unit A**	5.18	3.22	3.49	3.28	4.21	2.57	2.60
**Unit B**	4.85	3.25	3.64	3.44	4.36	2.56	3.77
**Unit C**	5.07	3.20	3.41	3.38	3.19	2.43	2.49
**Unit D**	5.00	3.10	3.41	3.38	3.54	3.28	3.39
**Unit E**	5.16	3.11	3.32	3.59	3.16	3.36	3.84
**Unit F**	5.14	3.30	3.64	3.38	4.03	3.60	4.26
**Unit G**	4.75	2.98	3.25	2.96	2.46	2.05	2.78

Complexes 7a,b ([Cu(mmp)(**4c**)]BF_4)

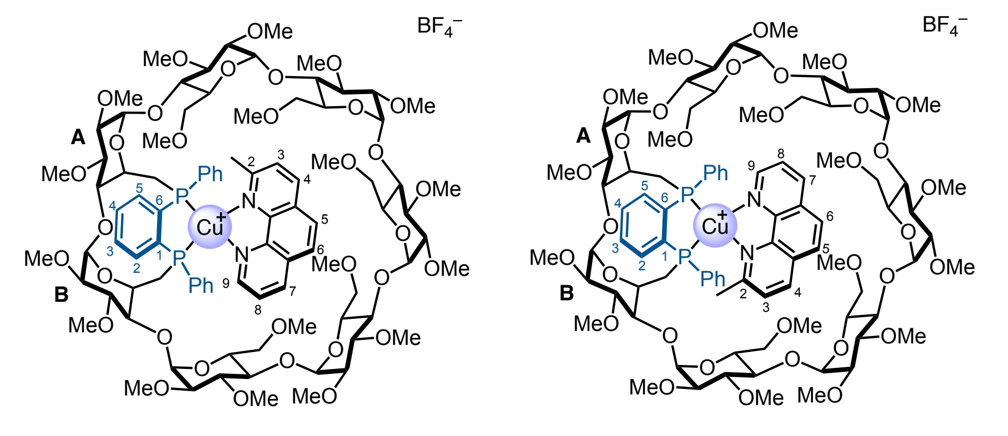

7a **7b**

$[Cu(CH_3CN)_4]BF_4$ (13.3 mg, 0.042 mmol) was added to a solution of **4c** (70 mg, 0.042 mmol) in a mixture of CH_2Cl_2/CH_3CN (5 mL, 7:3, *v*/*v*) at room temperature. The resulting solution was stirred for 0.5 h whereupon 2-methyl-1,10-phenanthroline (mmp) (13.3 mg, 0.042 mmol) was added. After 2 h, the solvents were removed *in vacuo* and recrystallization of the residue by slow diffusion of Et_2O into a CH_2Cl_2 solution of the crude product gave the 98:2 mixture of complexes **7a** and **7b** respectively (yield: 78 mg, 92%) as a yellow crystalline solid.

^{1}H NMR (500 MHz, CD_2Cl_2, 25°C) for **7a**: δ (assignment by combined COSY, ROESY, TOCSY and HSQC) = 1.31 (s, 3H, CH_3O), 1.84 (s, 3H, CH_3O), 1.89-2.32 (5H, H-6^{C,G}, H-5^{G}), 2.28 (s, Me_{Phen}), 2.51 (m, 1H, H-6a^{A}), 2.67 (m, 1H, H-6a^{B}), 2.73 (m, 1H, H-6b^{A}), 2.95 (s, 3H, CH_3O), 3.27 (s, 3H, CH_3O), 3.33 (s, 3H, CH_3O), 3.40 (s, 3H, CH_3O), 3.48 (s, 3H, CH_3O), 3.49 (s, 3H, CH_3O), 3.50 (s, 3H, CH_3O), 3.52 (s, 3H, CH_3O), 3.54 (s, 3H, CH_3O), 3.56 (s, 3H, CH_3O), 3.56 (s, 3H, CH_3O), 3.60 (s, 3H, CH_3O), 3.61 (s, 3H, CH_3O), 3.64 (s, 3H, CH_3O), 3.69 (s, 6H, CH_3O), 3.81 (s, 3H, CH_3O), 2.89-3.88 (30H, H-2, H-3, H-4, H-5^{C,D,F}, H-6^{D,F}, H-6a^{E}, H-6b^{B}), 4.05 (m, 1H, H-6b^{E}), 4.20 (m, 1H, H-5^{E}), 4.35 (m, 1H, H-5^{A}), 4.50 (m, 1H, H-5^{B}), 4.63 (d, $^3J_{H-2,H-1}$ = 3.2 Hz, 1H, H-1), 4.84 (d, $^3J_{H-2,H-1}$ = 3.5 Hz, 1H, H-1^{B}), 4.92 (d, $^3J_{H-2,H-1}$ = 3.2 Hz, 1H, H-1), 5.02 (d, $^3J_{H-2,H-1}$ = 3.3 Hz, 1H, H-1), 5.04 (d, $^3J_{H-2,H-1}$ = 3.4 Hz, 1H, H-1), 5.23 (d, $^3J_{H-2,H-1}$ = 3.7 Hz, 1H, H-1), 5.35 (d, $^3J_{H-2,H-1}$ = 4.0 Hz, 1H, H-1^{A}), 7.16-7.50 (10H, $H_{orthoPh}$, H_{metaPh}, H_{paraPh}, H-3$_{arom}$, H-3$_{Phen}$), 7.64 (m, 1H, H-4$_{arom}$), 7.75 (m, 1H, H-5$_{arom}$), 7.77 (m, 2H, $H_{orthoPh}$), 7.91 (m, 1H, H-6$_{arom}$), 8.00 (d, $^3J_{H-6,H-5}$ = 8.7 Hz, 1H, H-5$_{Phen}$), 8.06 (d, $^3J_{H-5,H-6}$ = 8.7 Hz, 1H, H-6$_{Phen}$), 8.07 (m, 1H, H-8$_{Phen}$), 8.34 (d, $^3J_{H-3,H-4}$ = 8.2 Hz, 1H, H-4$_{Phen}$), 8.70 (d, $^3J_{H-8,H-7}$ = 7.9 Hz, 1H, H-7$_{Phen}$), 10.01 (br d, 1H, H-9$_{Phen}$) ppm;

^{13}C{^{1}H} NMR (126 MHz, CD_2Cl_2, 25°C) for **7a**: δ (assignment by HSQC) = 28.88 (s, Me_{Phen}), 35.23 and 35.34 (br d, C-6^{A,B}), 57.39, 57.72, 58.12, 58.26, 58.60, 58.67, 58.89, 59.10 [x2], 59.43, 59.50 and 61.32 (CH_3O-2 and CH_3O-6), 60.50, 60.86, 60.92, 60.95, 61.27, 61.59 and 62.17 (CH_3O-3), 69.88, 71.35, 71.56, 72.16 and 73.65 (C-5^{C,D,E,F,G}), 70.82, 70.86, 71.65, 72.10 and 72.96 (C-6^{C,D,E,F,G}), 71.67 (d, $^2J_{P,C}$ = 15.5 Hz, C-5^{A}), 74.60 (d, $^2J_{P,C}$ = 8.6 Hz, C-5^{B}), 78.28, 80.50 [x2], 80.58, 80.72, 81.16, 81.27, 81.51, 81.73, 82.16, 82.22, 82.30, 82.51 [x2], 82.61 [x2], 82.79, 82.80 and 83.05 (C-2, C-3, C-4^{C,D,E,F,G}), 86.45 (br d, C-4^{B}), 88.54 (d, $^3J_{P,C}$ = 11.6 Hz, C-4^{A}), 98.16, 98.47, 99.12, 99.35, 99.67, 100.19 and 101.66 (C-1), 124.73 (s, C-8$_{Phen}$), 125.71 (s, C-3$_{Phen}$), 126.77

(s, C-6$_{Phen}$), 127.40 (s, C-5$_{Phen}$), 127.88 (s, C$_{quatPhen}$), 128.95 (d, $^3J_{P,C}$ = 8.3 Hz, C$_{metaPh}$), 129.13 (d, $^3J_{P,C}$ = 8.2 Hz, C$_{metaPh}$), 129.51 (s, C$_{paraPh}$), 130.02 (s, C$_{paraPh}$), 130.12 (s, C$_{quatPhen}$), 131.38 (d, $^2J_{P,C}$ = 12.4 Hz, C$_{orthoPh}$), 131.63 (m, C-5$_{arom}$), 132.27 (m, C-4$_{arom}$), 132.66 (d, $^2J_{P,C}$ = 13.1 Hz, C$_{orthoPh}$), 133.22 (d, $^2J_{P,C}$ = 9.2 Hz, C-3$_{arom}$), 134.22 (dd, $^1J_{P,C}$ = 19.6 Hz, $^3J_{P,C}$ = 15.6 Hz, C$_{ipsoPh}$), 134.82 (d, $^2J_{P,C}$ = 7.6 Hz, C-6$_{arom}$), 135.49 (br dd, C$_{ipsoPh}$), 137.66 (s, C-7$_{Phen}$), 137.90 (s, C-4$_{Phen}$), 138.95 (dd, $^1J_{P,C}$ = 36.3 Hz, $^2J_{P,C}$ = 27.0 Hz, C-1$_{arom}$), 143.89 (s, C$_{quatPhen}$), 145.48 (s, C$_{quatPhen}$), 146.99 (dd, $^1J_{P,C}$ = 39.3 Hz, $^2J_{P,C}$ = 32.8 Hz, C-2$_{arom}$), 151.17 (s, C-9$_{Phen}$), 159.62 (s, C-2$_{Phen}$) ppm;

$^{31}P\{^1H\}$ NMR (202.5 MHz, CD_2Cl_2, 25°C) for **7a,b**: δ = –8.2 (d, $^2J_{P,P}$ = 196 Hz, P_2), 1.6 (d, $^2J_{P,P}$ = 196 Hz, P_1) ppm (**7a**);

elemental analysis (%) calcd for $C_{92}H_{130}O_{33}P_2N_2CuBF_4$.1/3$CH_2Cl_2$: C 54.56, H 6.48, N 1.38, found: C 54.50, H 6.61, N 1.35;

MS (ESI-TOF) for $C_{91}H_{128}O_{33}P_2N_2CuBF_4$: *m/z* (%): 1915.73 (100) [*M* – BF_4]$^+$.

Full ^{1}H NMR assignement of all glucose units of compound **7a** was achieved by combined COSY, ROESY, TOCSY and HSQC (δ values in ppm).

	H-1	H-2	H-3	H-4	H-5	H-6a	H-6b
**Unit A**	5.35	3.21	3.51	3.27	4.35	2.51	2.73
**Unit B**	4.84	3.31	3.70	3.38	4.50	2.67	3.50
**Unit C**	4.92	3.08	3.41	3.21	3.02	2.21	2.27
**Unit D**	5.02	3.10	3.47	3.56	3.27	3.33	3.69
**Unit E**	5.23	3.42	3.76	3.66	4.20	3.85	4.05
**Unit F**	5.04	3.13	3.44	3.46	3.44	3.03	3.09
**Unit G**	4.63	2.91	3.34	3.06	2.27	1.90	2.03

Complex 8 ([Cu(phen)(**4c**)]PF_6)

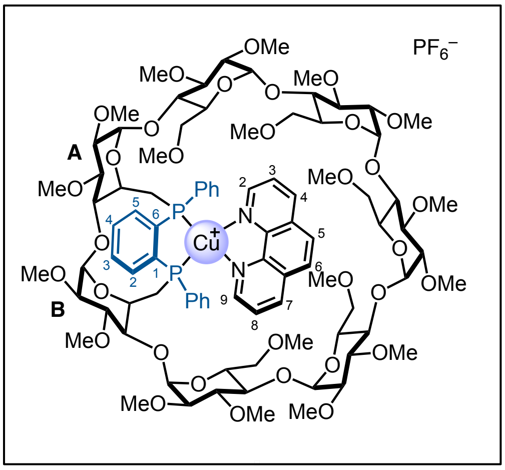

KPF_6 (46 mg, 0.25 mmol) was added to a solution of **5** (10 mg, 0.005 mmol) in a mixture of dichloromethane and water (10 mL, CH_2Cl_2/H_2O, 5:5, *v*/*v*). The reaction mixture was stirred at 25°C for 0.5 h. The organic solution was washed with water (2 x 5 mL) and subsequently dried ($MgSO_4$) before being evaporated in vacuo. Recrystallization of the residue by slow diffusion of Et_2O into a CH_2Cl_2 solution of the crude product gave **8** (yield: 9.4 mg, 91%) as a yellow crystalline solid.

^{1}H NMR and ^{13}C NMR spectra of compound **8** were identical to those of compound **5** outlined above.

$^{31}P\{^1H\}$ NMR (202.5 MHz, CD_2Cl_2, 25°C): δ = –144.5 (sep, $^1J_{P,F}$ = 710 Hz, PF_6), – 4.6 (d, $^2J_{P,P}$ = 186 Hz, P_2), 2.4 (d, $^2J_{P,P}$ = 186 Hz, P_1) ppm;

elemental analysis (%) calcd for $C_{91}H_{128}O_{33}P_2N_2CuPF_6.4CH_2Cl_2$: C 47.78, H 5.74, N 1.17, found: C 47.44, H 6.07, N 1.20;

MS (ESI-TOF) for $C_{91}H_{128}O_{33}P_2N_2CuPF_6$: *m/z* (%): 1901.72 (100) $[M - PF_6]^+$.

NMR and mass spectra

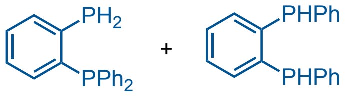

1

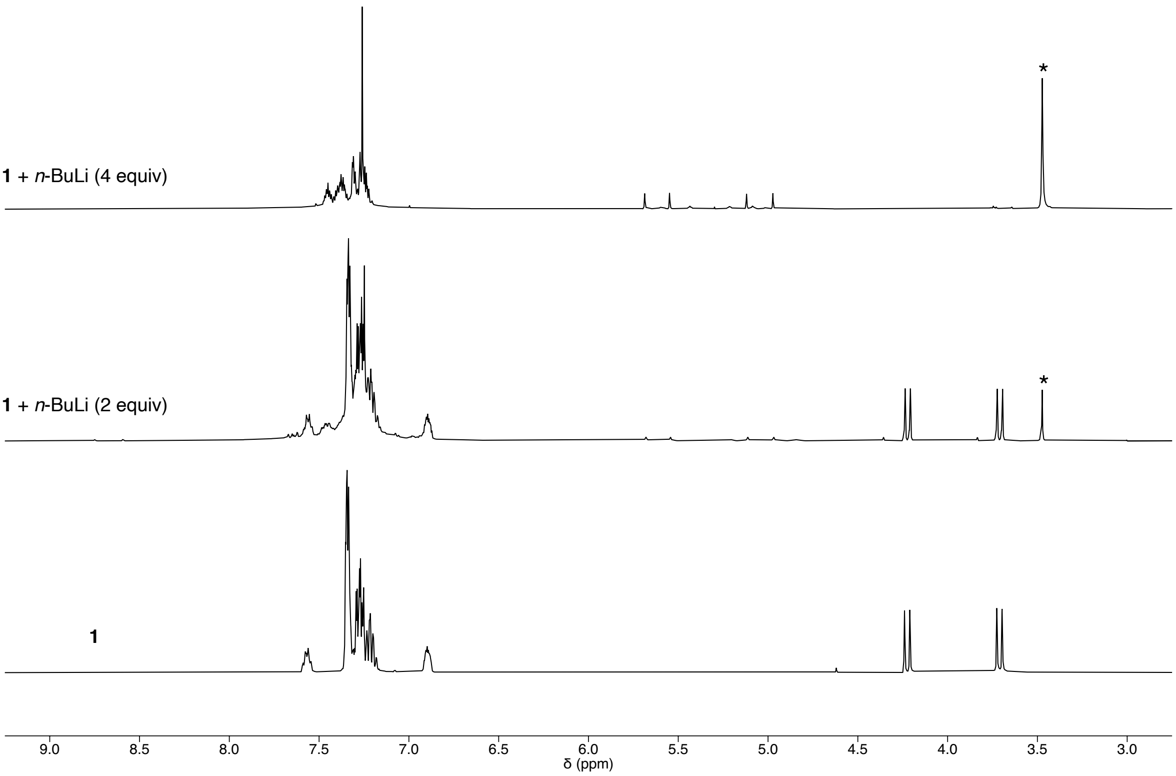

Figure S1a. ^{1}H NMR (400 MHz, $CDCl_3$) spectrum of compound **1** in presence of *n*-BuLi for 1 h at room temperature after protonation with an excess of methanol (* = traces of methanol).

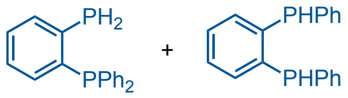

1

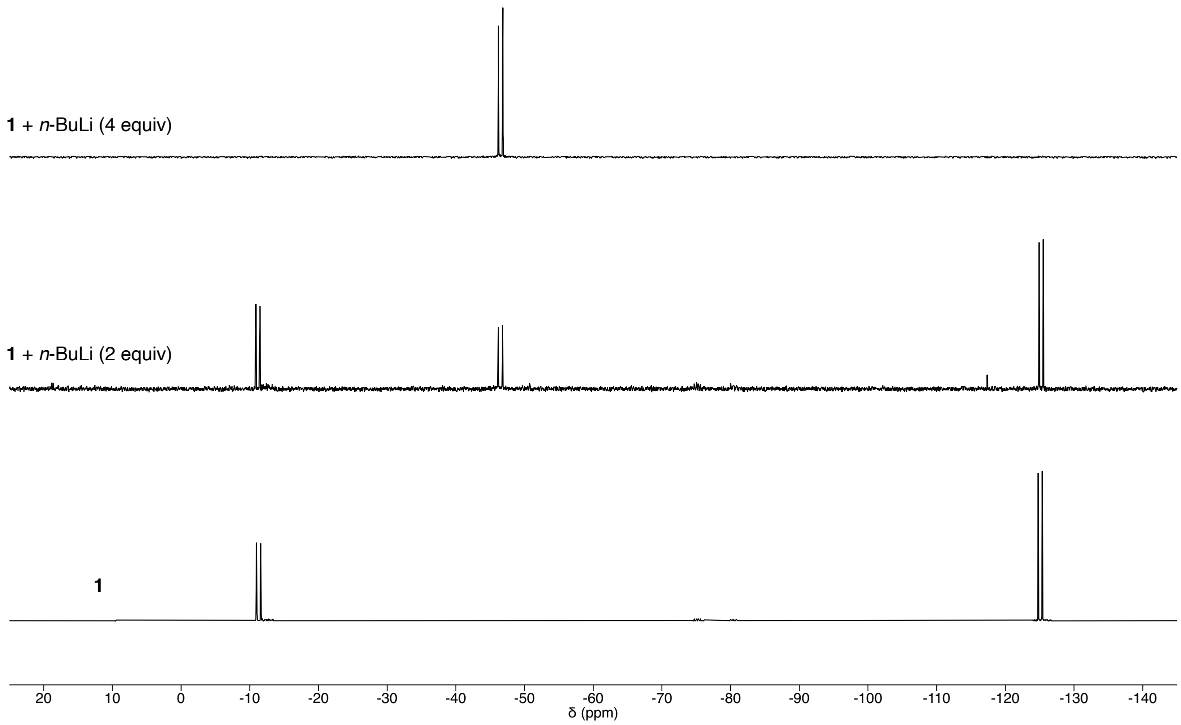

Figure S1b. $^{31}P\{^{1}H\}$ NMR (162 MHz, $CDCl_3$) spectrum of compound **1** in presence of *n*-BuLi for 1 h at room temperature after protonation with an excess of methanol.

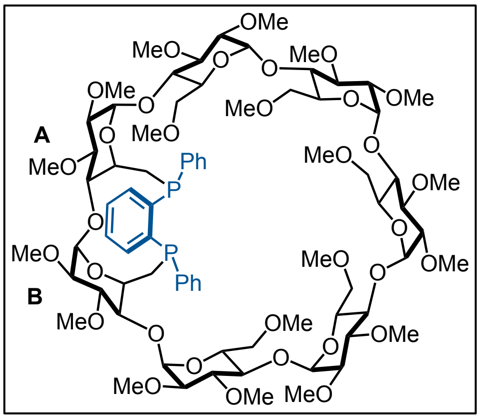

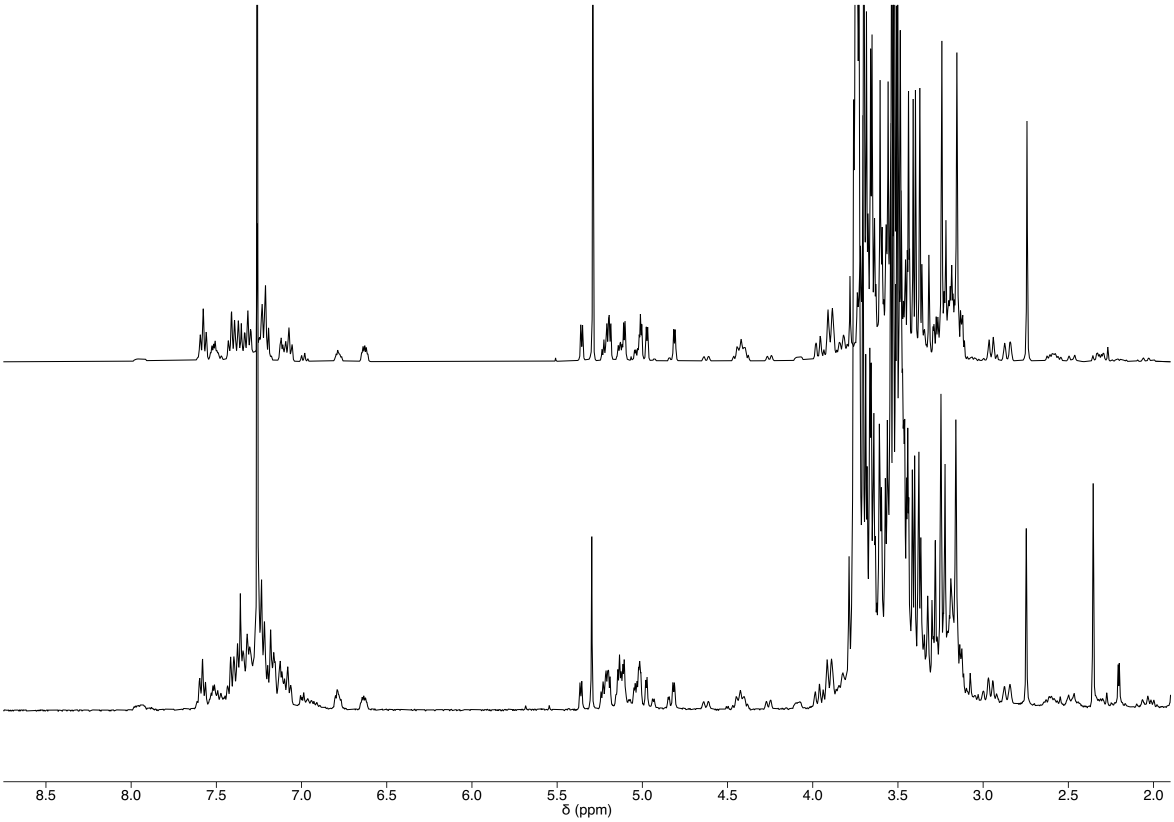

Figure S2a. ^{1}H NMR (400 MHz, $CDCl_3$) spectrum of a mixture of **4a**, **4b** and **4c** before (bottom) and after (top) 4 h reflux in mesitylene.

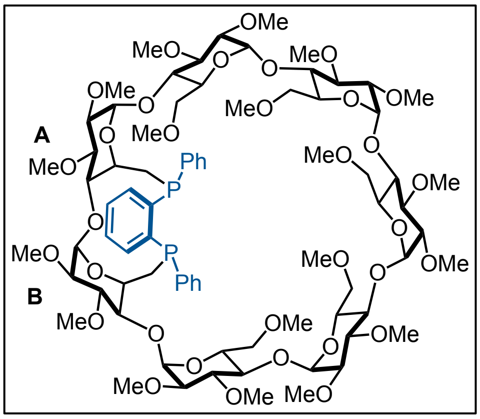

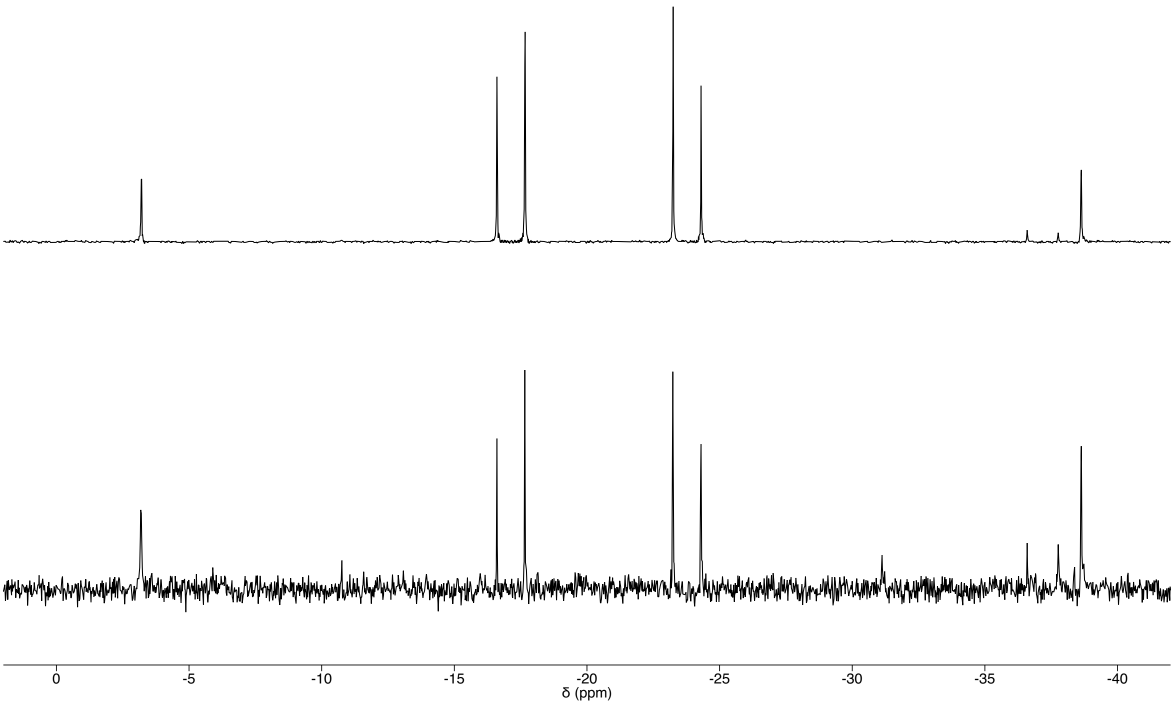

Figure S2b. $^{31}P\{^{1}H\}$ NMR (162 MHz, $CDCl_3$) spectrum of a mixture of **4a**, **4b** and **4c** before (bottom) and after (top) 4 h reflux in mesitylene.

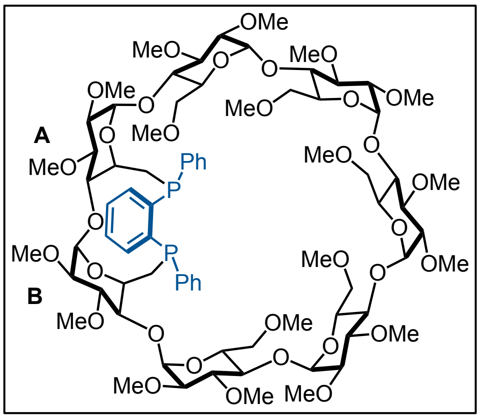

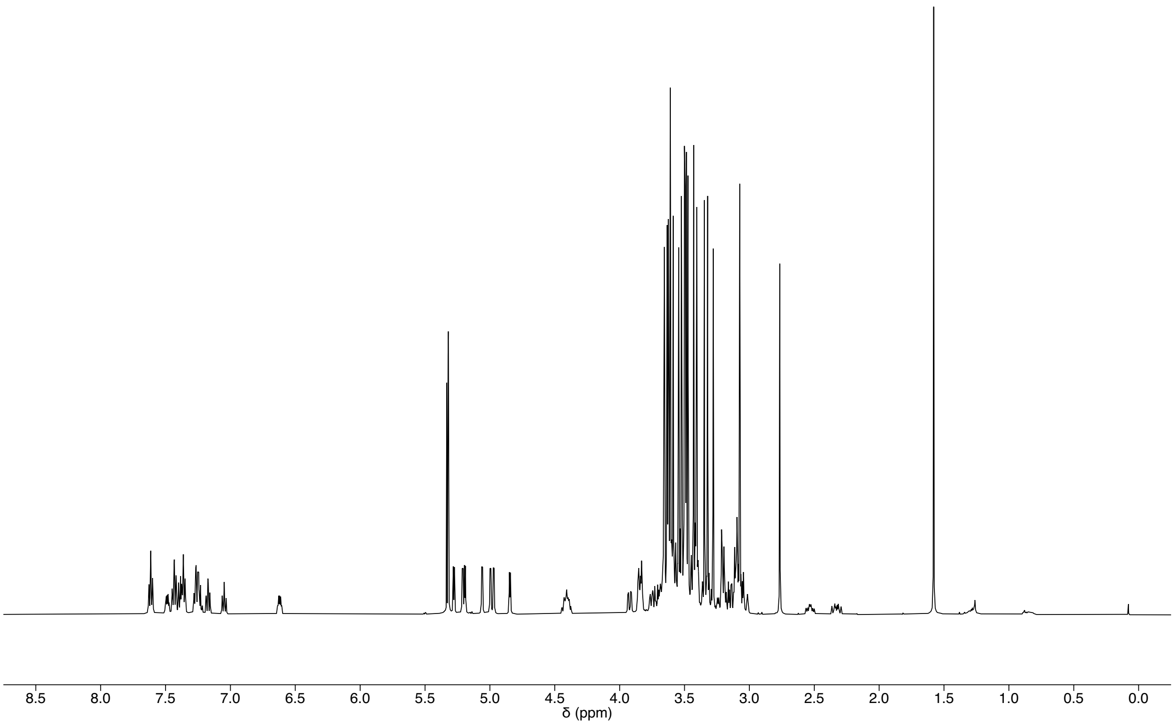

Figure S3a. ^{1}H NMR (500 MHz, $CDCl_3$) spectrum of compound **4c**.

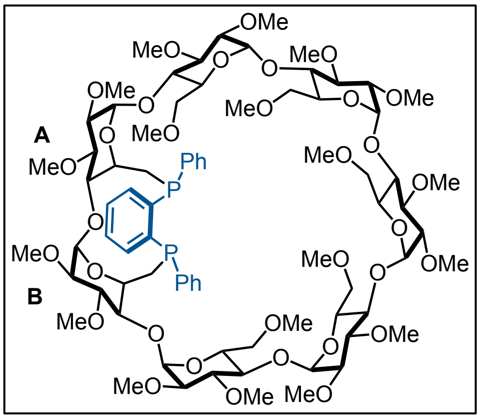

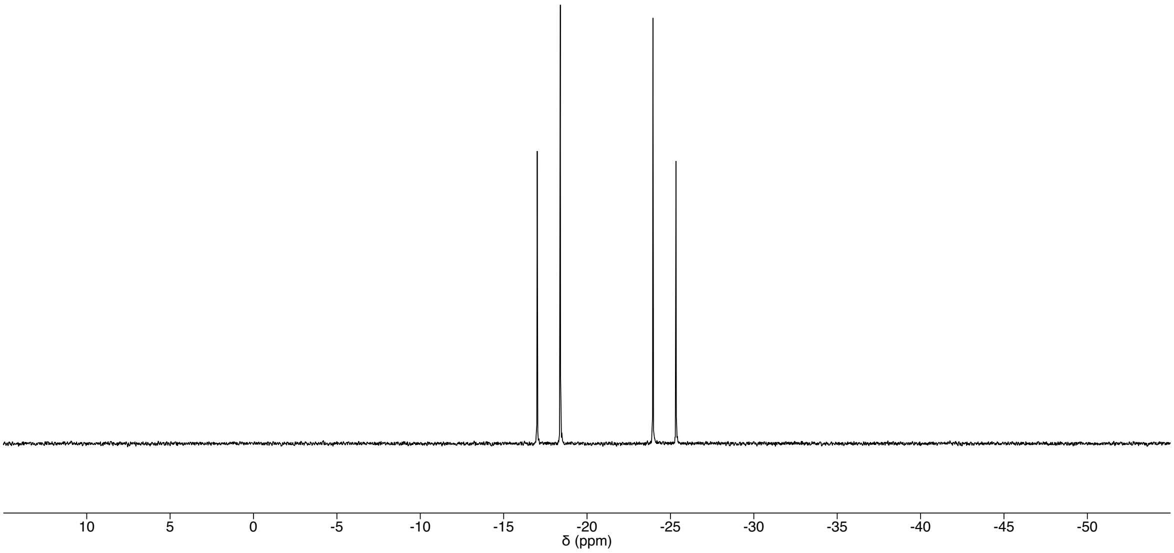

Figure S3b. $^{31}P\{^{1}H\}$ NMR (121.5 MHz, $CDCl_3$) spectrum of compound **4c**.

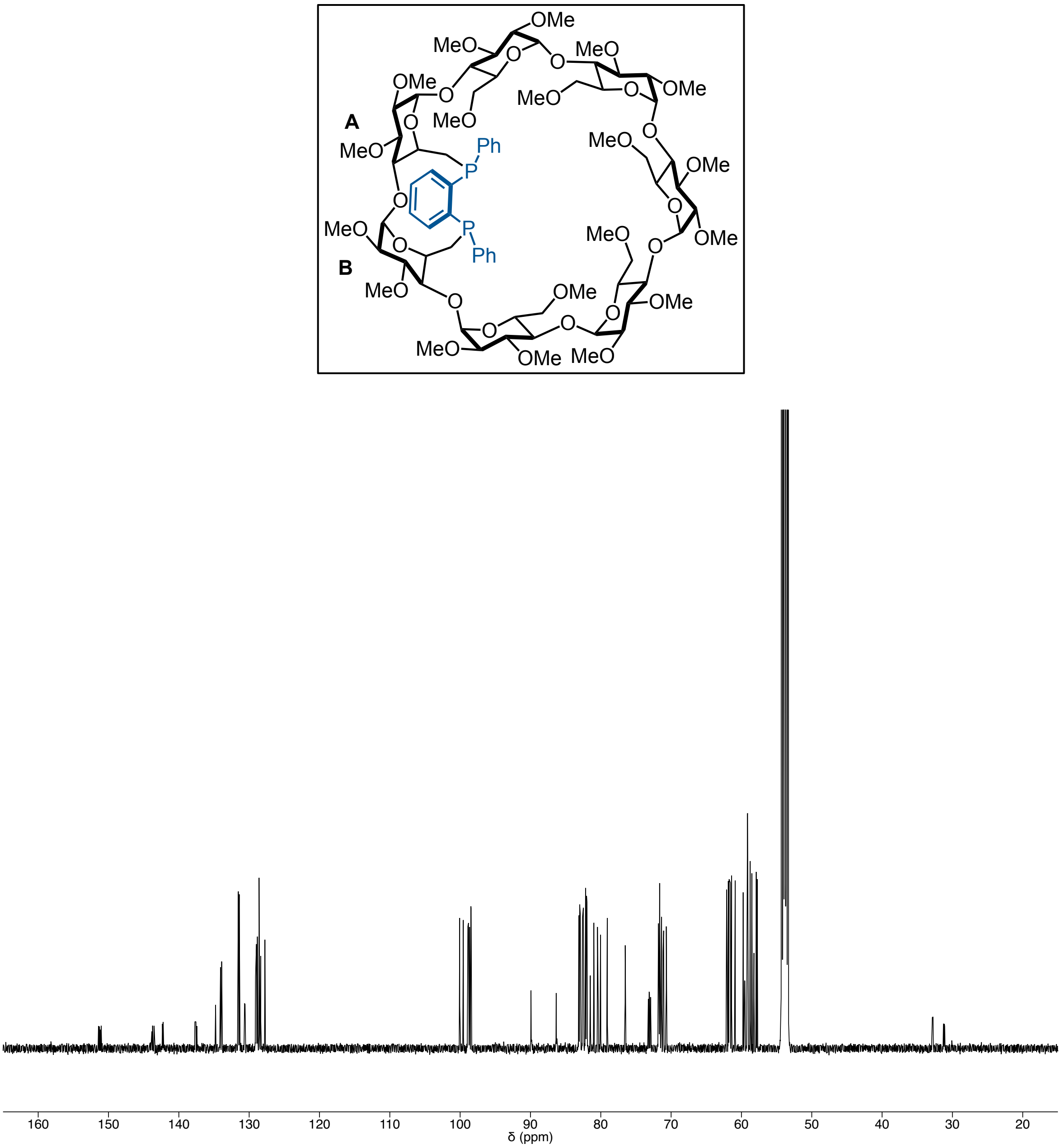

Figure S3c. $^{13}C\{^{1}H\}$ NMR (126 MHz, $CDCl_3$) spectrum of compound **4c**.

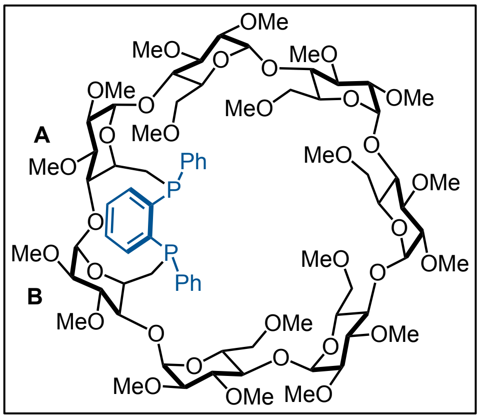

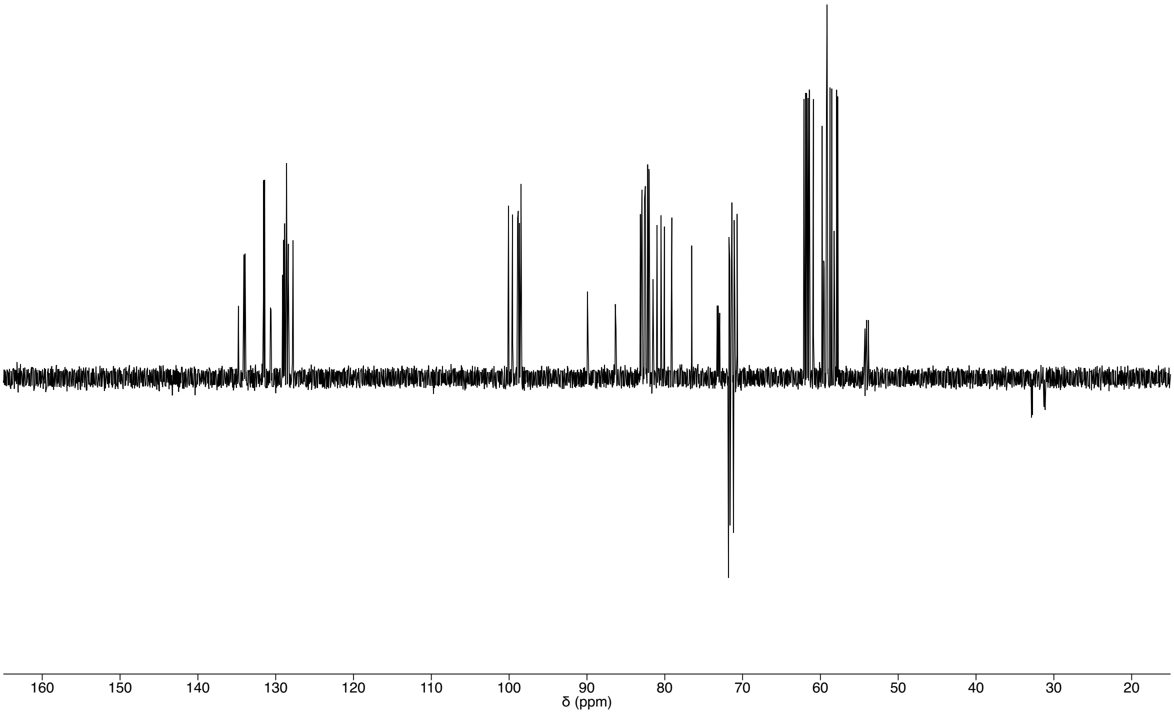

Figure S3d. DEPT 135 NMR (126 MHz, $CDCl_3$) spectrum of compound **4c**.

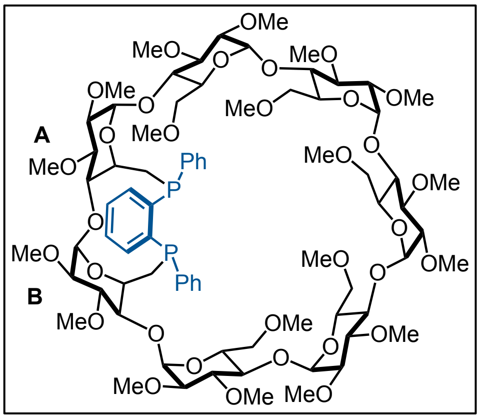

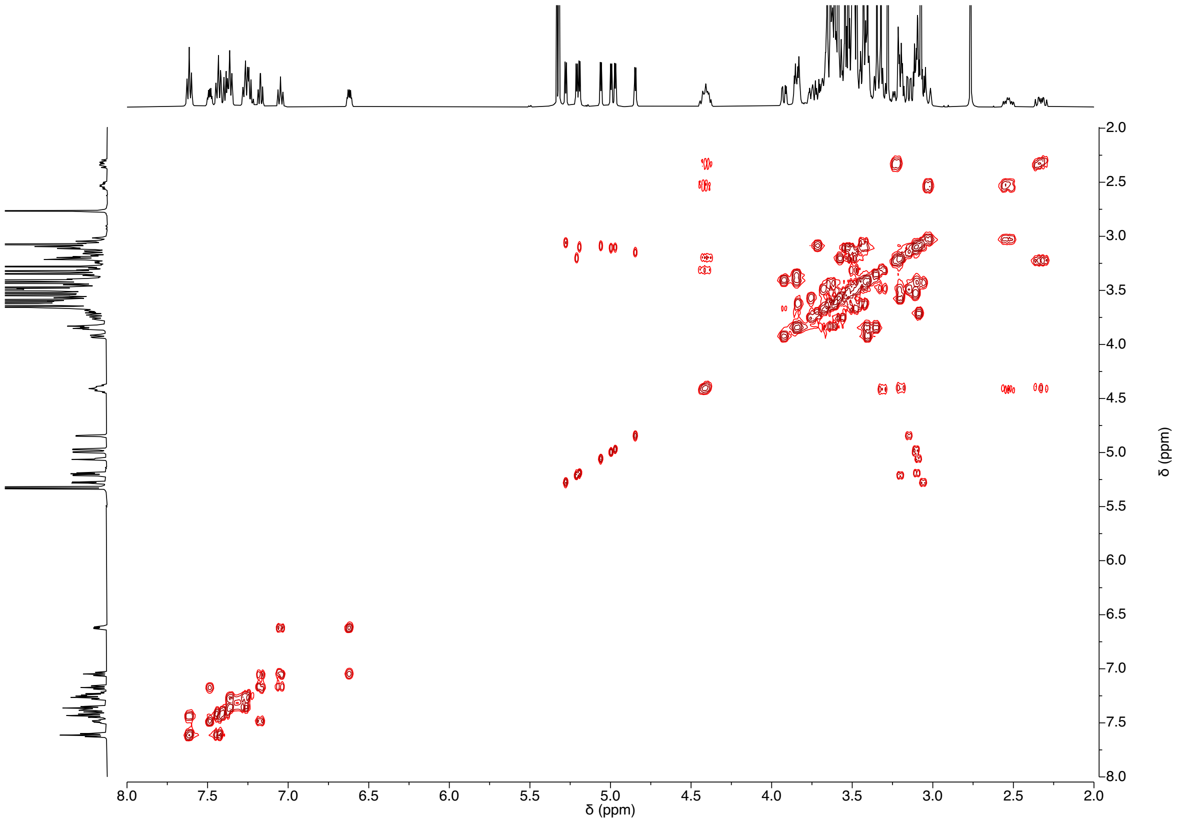

Figure S3e. ^{1}H/^{1}H COSY NMR (500 MHz, $CDCl_3$) spectrum of compound **4c**.

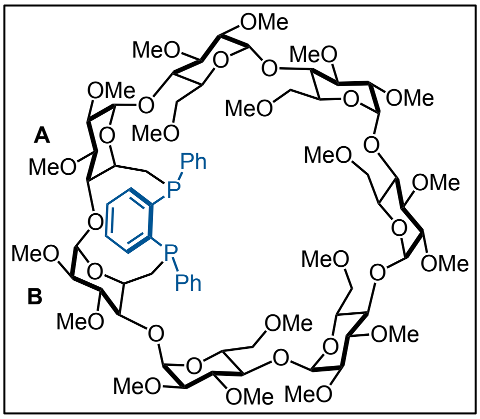

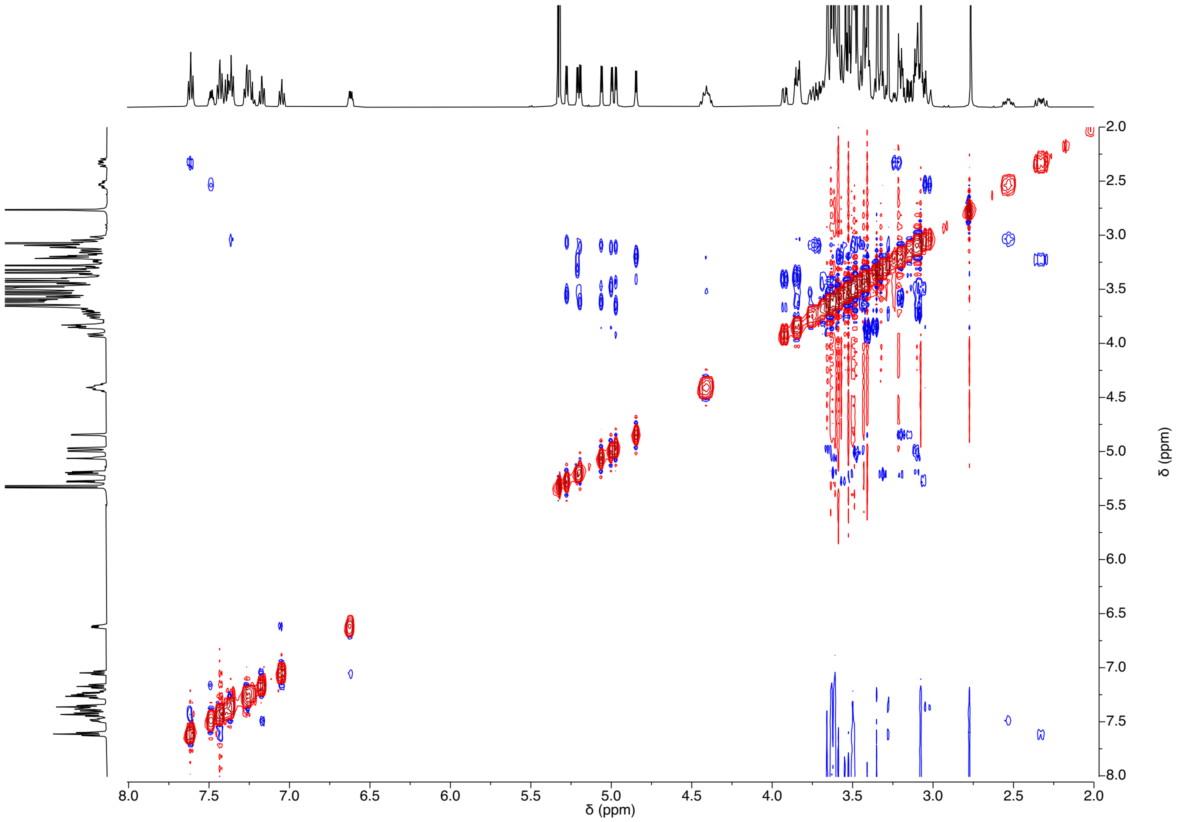

Figure S3f. ^{1}H/^{1}H ROESY NMR (500 MHz, $CDCl_3$) spectrum of compound **4c**.

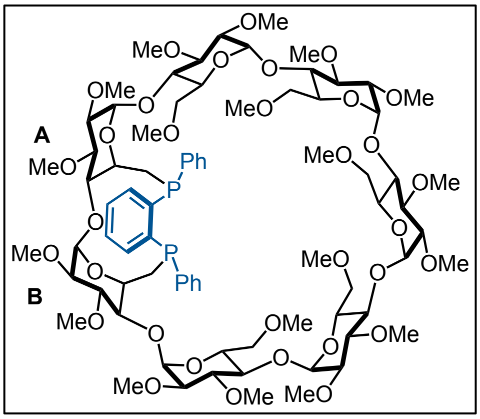

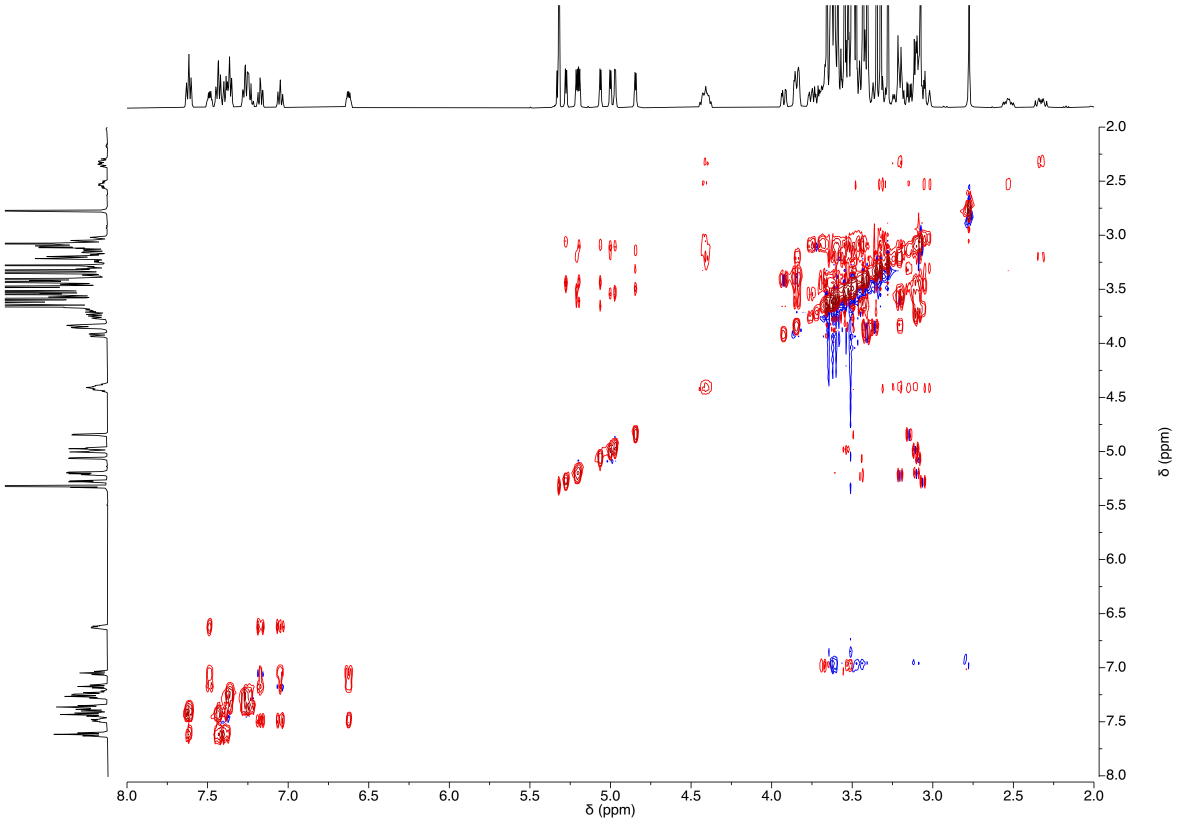

Figure S3g. $^{1}H/^{1}H$ TOCSY NMR (500 MHz, $CDCl_3$) spectrum of compound **4c**.

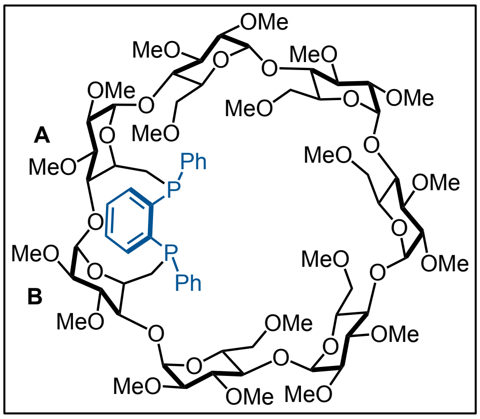

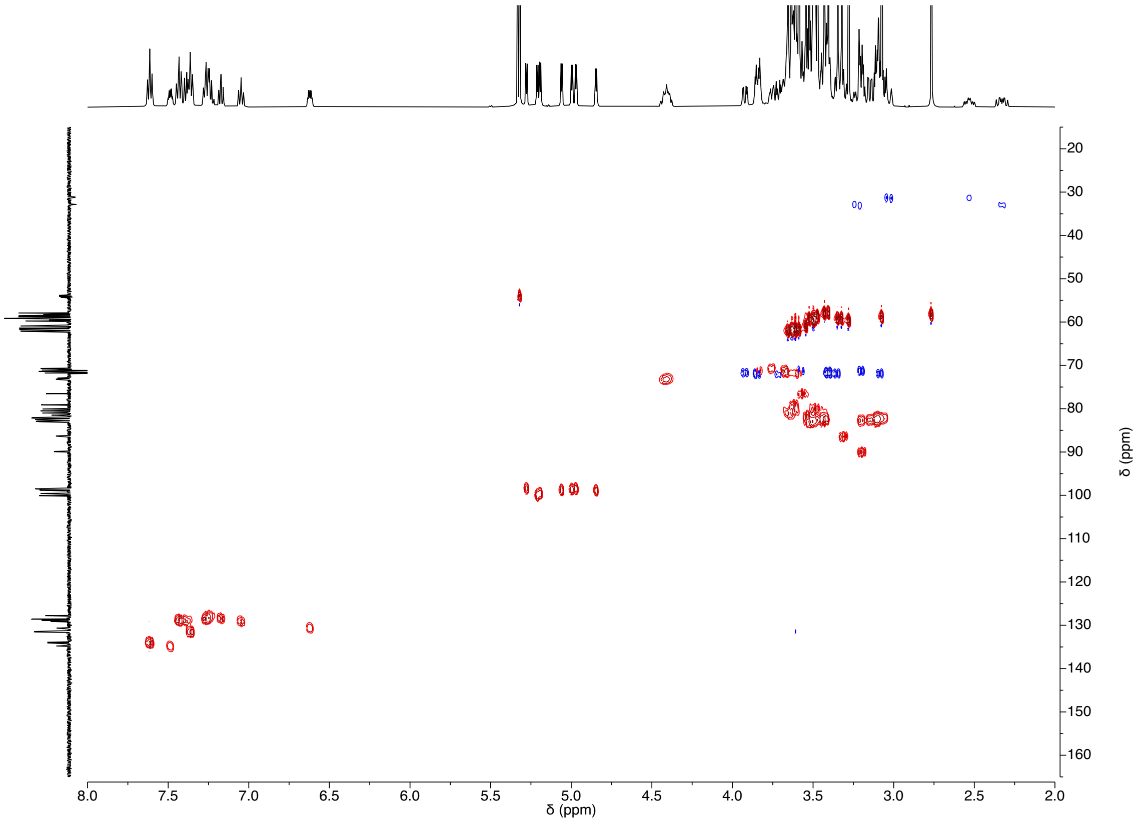

Figure S3h. $^{1}H/^{13}C\{^{1}H\}$ HSQC NMR (500 MHz, $CDCl_3$) spectrum of compound **4c**.

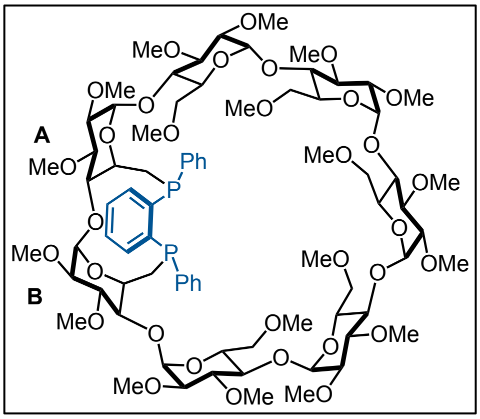

Acquisition Parameter

Source Type	ESI	Capillary	4500 V	Nebulizer	0.3 Bar	Set Hexapole RF	330.0 Vpp
Ion Polarity	Positive	Dry Heater	200 °C	Dry Gas	3.0 l/min	Set Capillary Exit	150.0 V

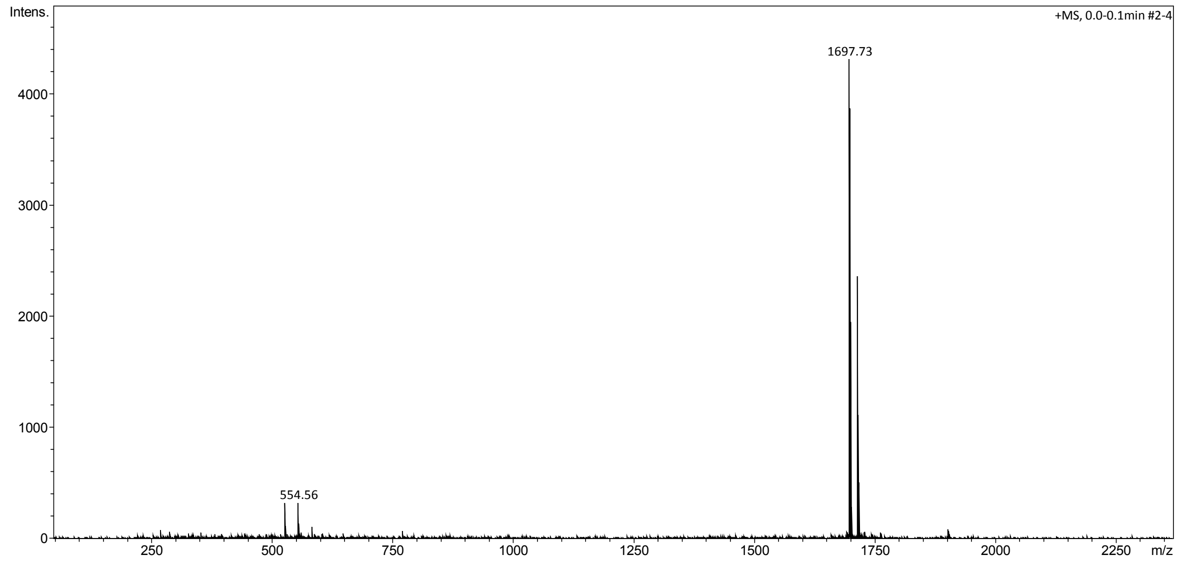

Figure S3i. ESI-TOF mass spectrum of compound **4c**.

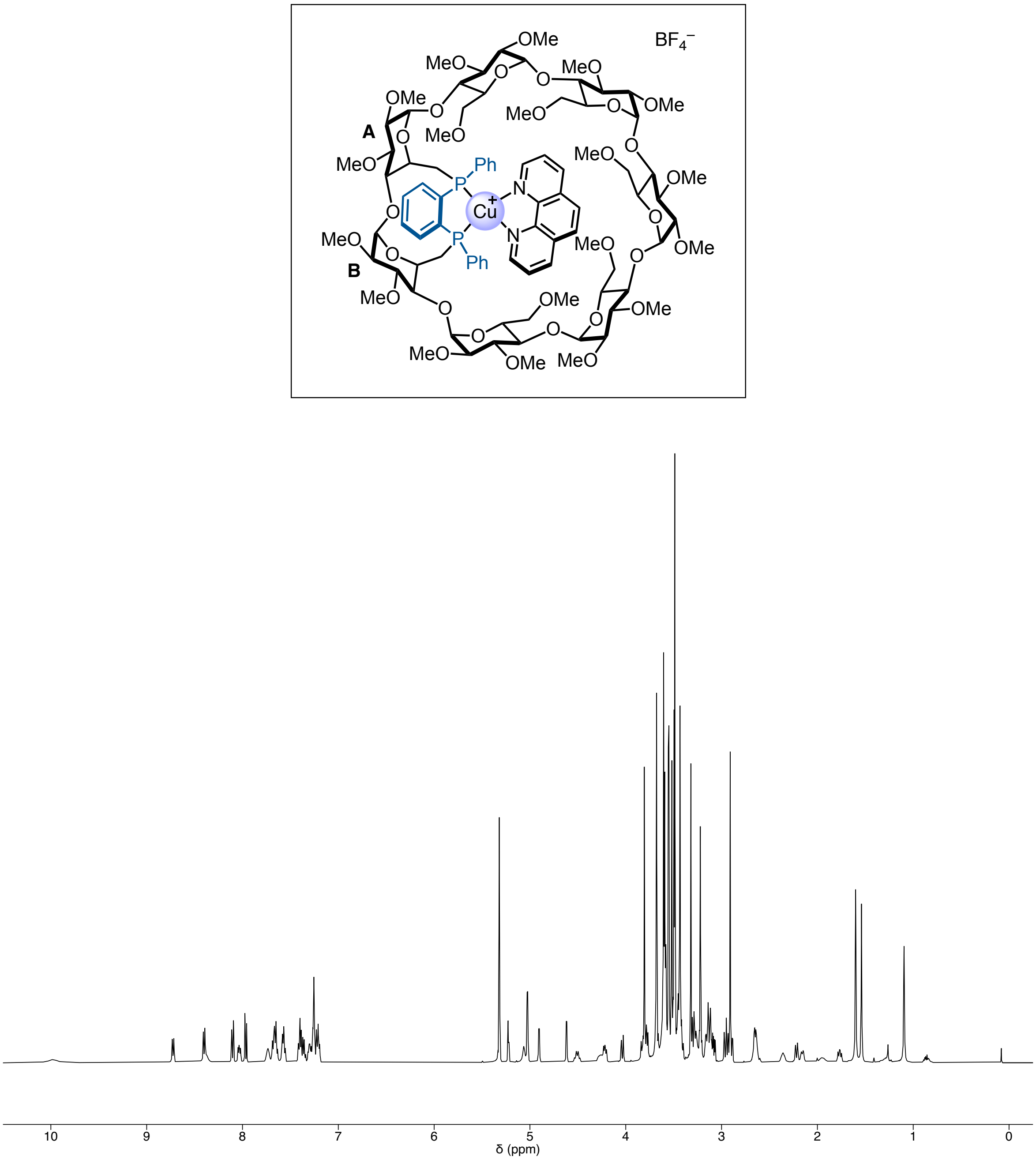

Figure S4a. ^{1}H NMR (500 MHz, CD_2Cl_2) spectrum of compound **5**.

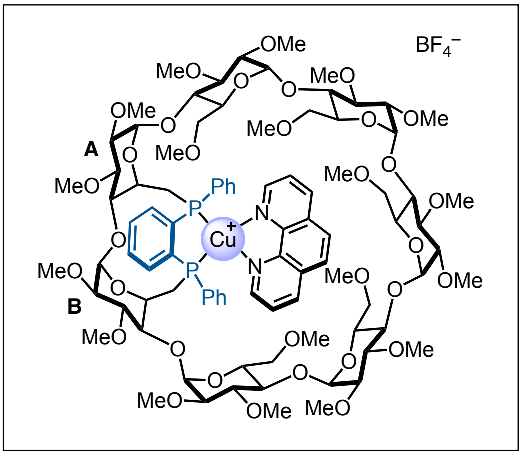

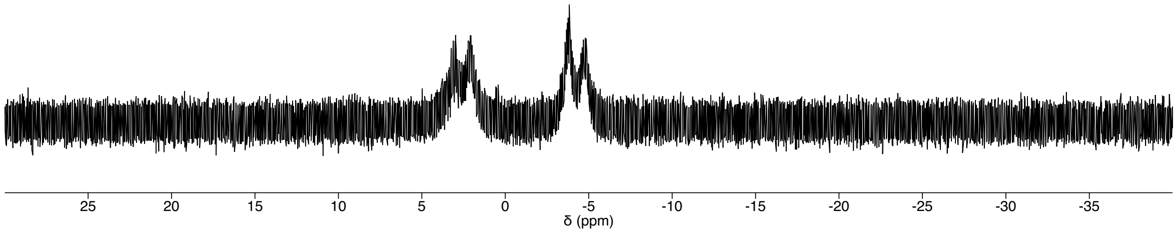

Figure S4b. $^{31}P\{^1H\}$ NMR (202.5 MHz, CD_2Cl_2) spectrum of compound **5**.

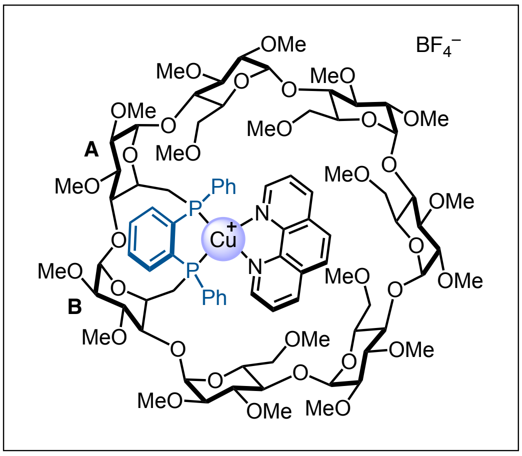

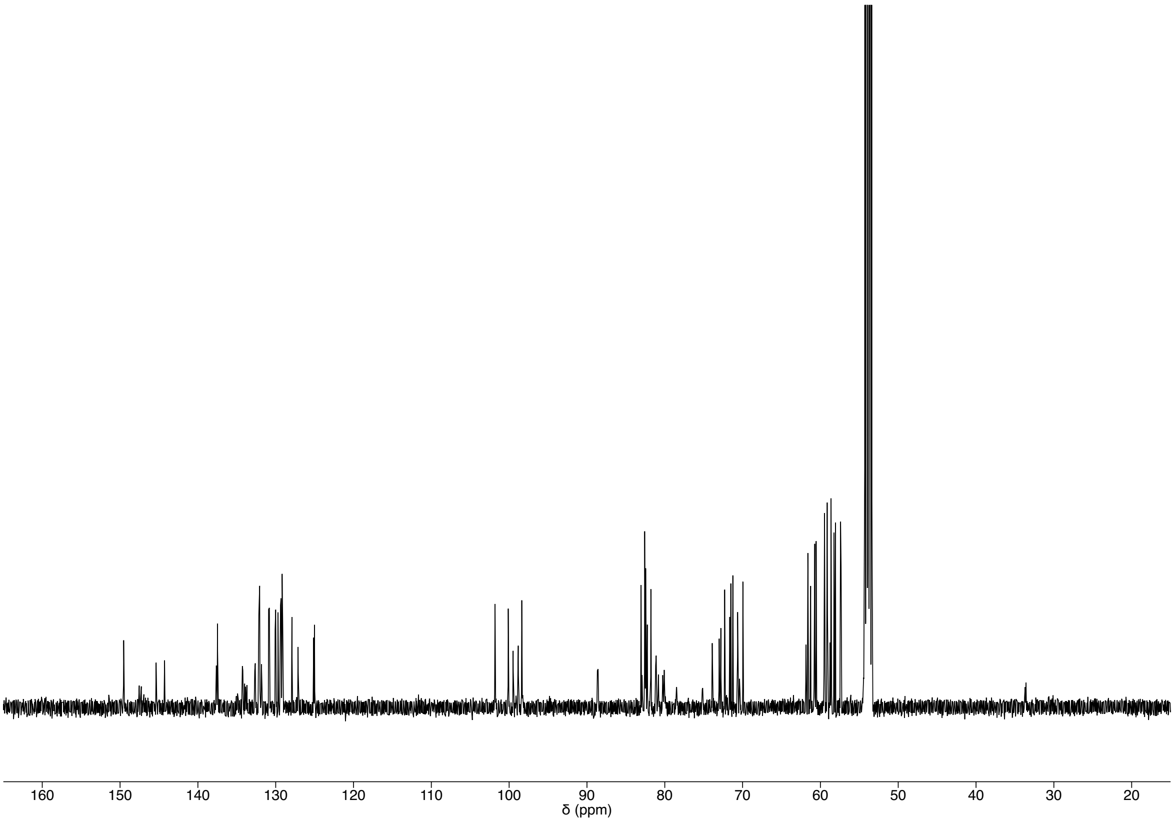

Figure S4c. $^{13}C\{^{1}H\}$ NMR (126 MHz, CD_2Cl_2) spectrum of compound **5**.

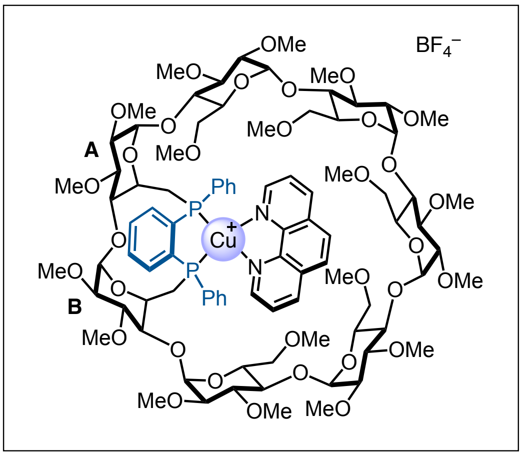

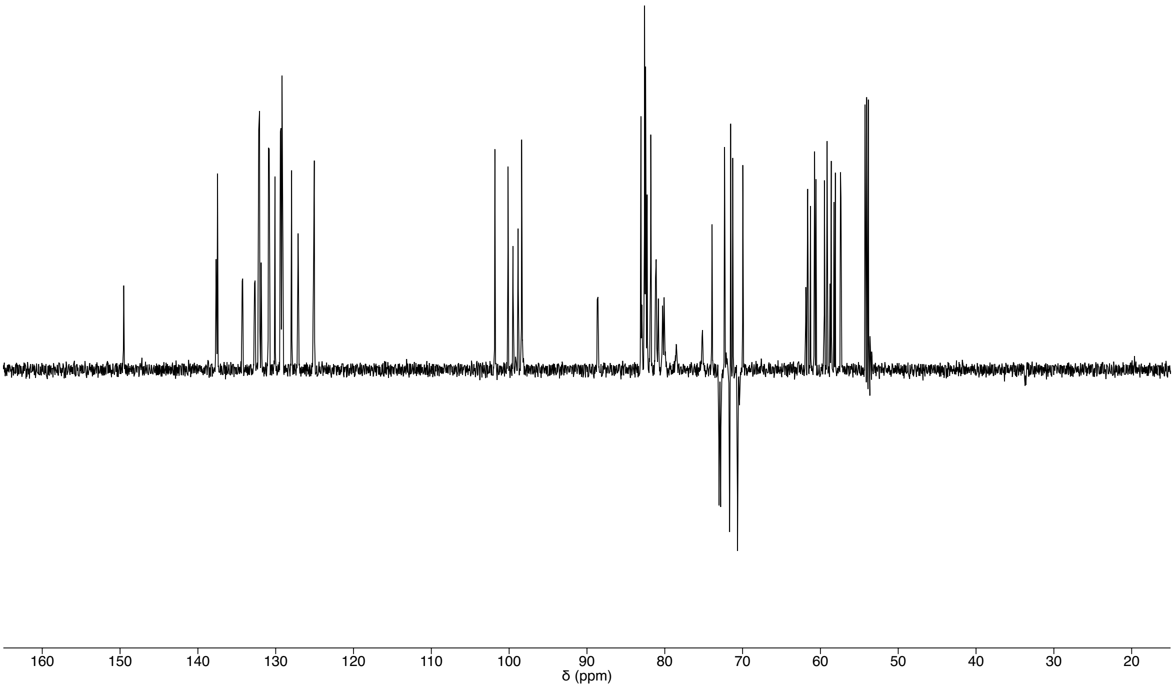

Figure S4d. DEPT 135 NMR (126 MHz, CD_2Cl_2) spectrum of compound **5**.

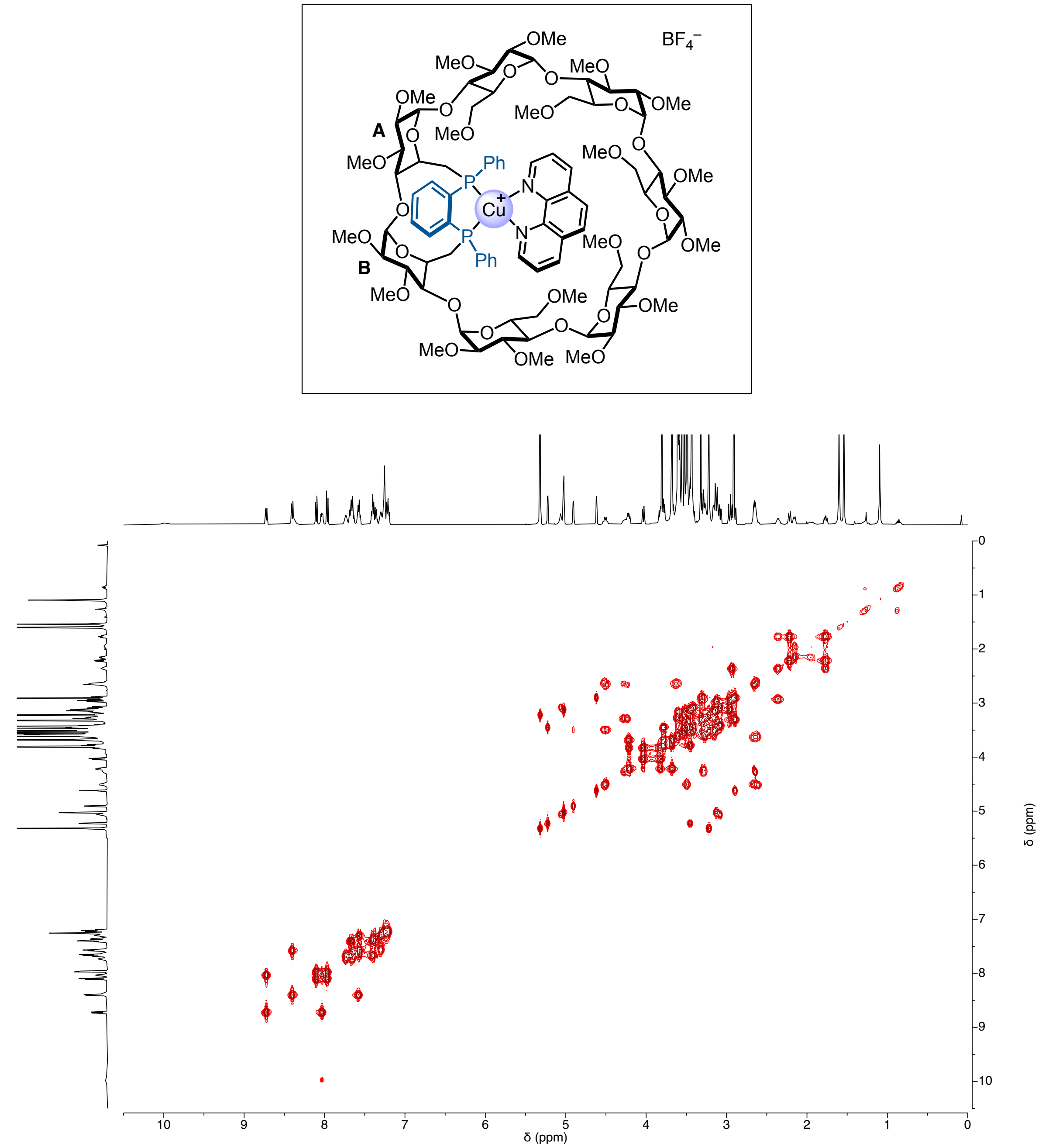

Figure S4e. $^1H/^1H$ COSY NMR (500 MHz, CD_2Cl_2) spectrum of compound **5**.

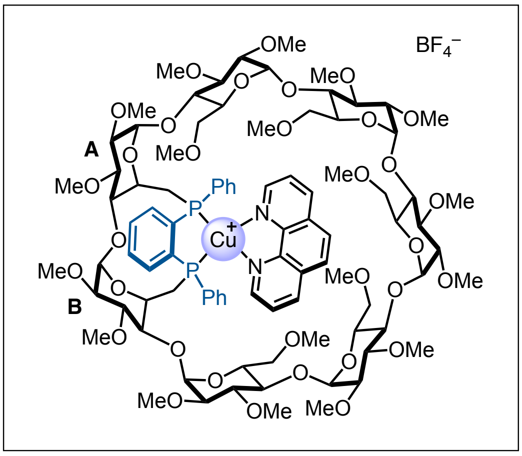

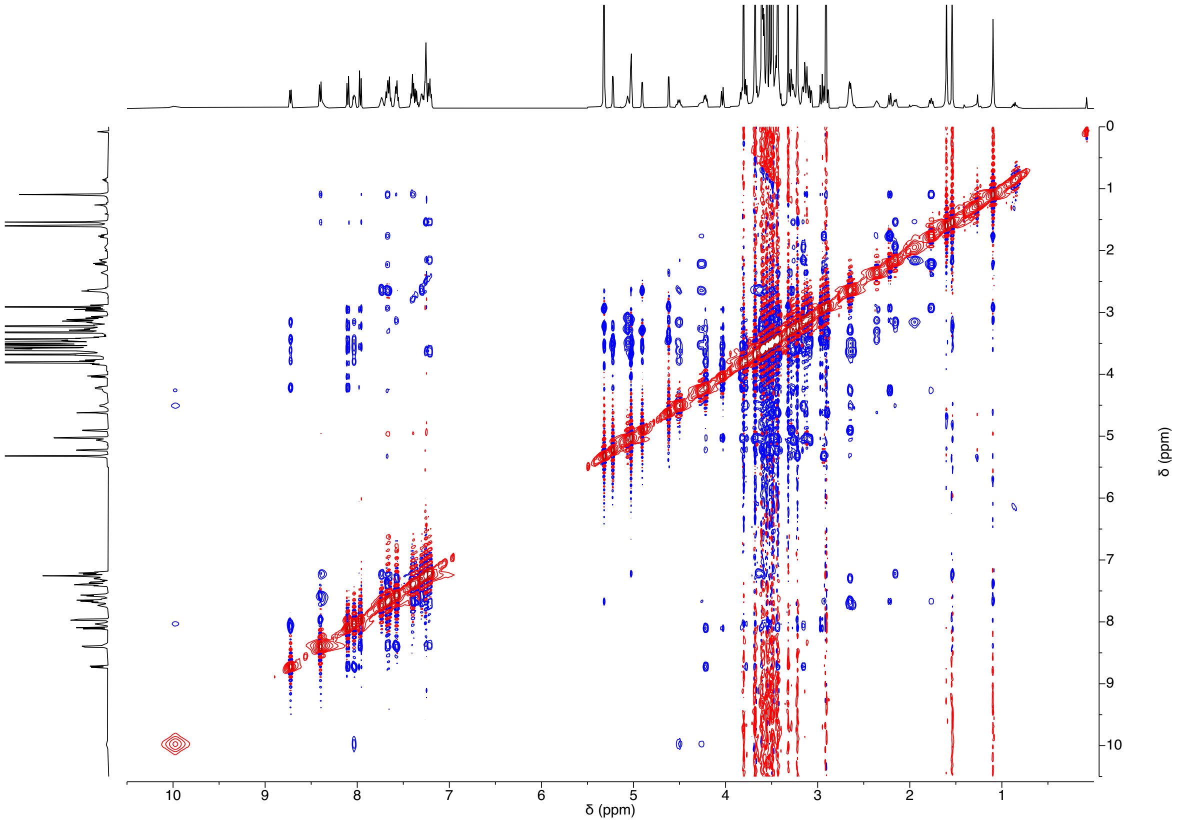

Figure S4f. $^1H/^1H$ ROESY NMR (500 MHz, CD_2Cl_2) spectrum of compound **5**.

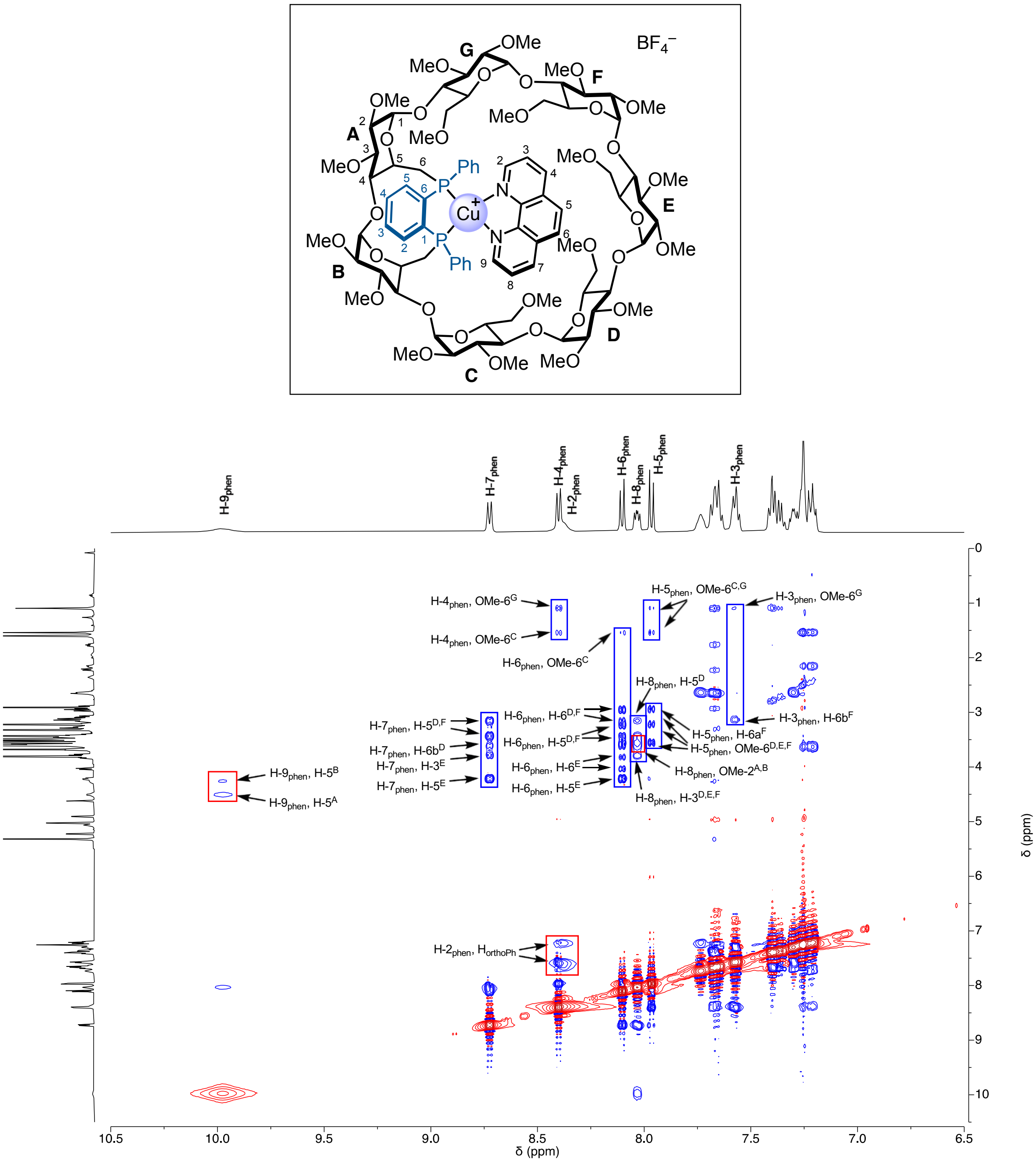

Figure S4g. Part of the ^{1}H/^{1}H ROESY NMR (500 MHz, CD_2Cl_2) spectrum of compound **5** showing cross peaks arising from through-space correlations between phenanthroline and CD protons.

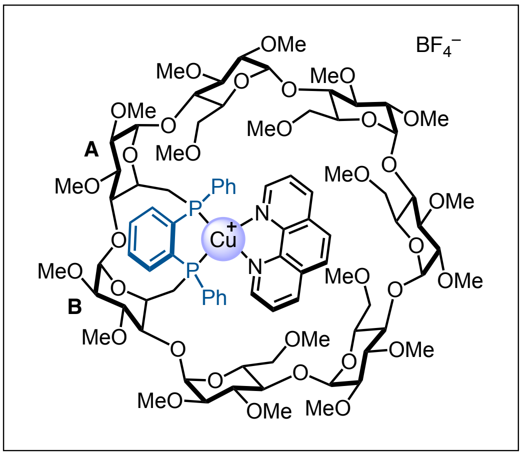

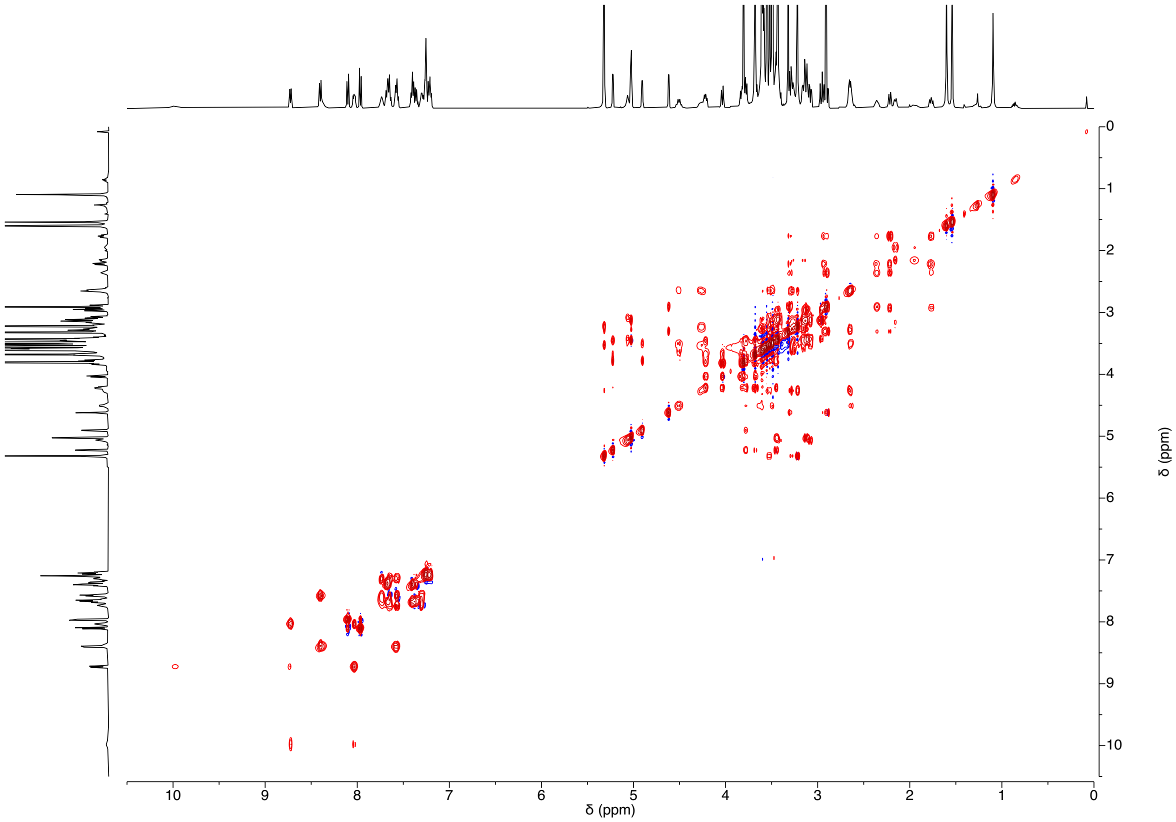

Figure S4h. ^{1}H/^{1}H TOCSY NMR (500 MHz, CD_2Cl_2) spectrum of compound **5**.

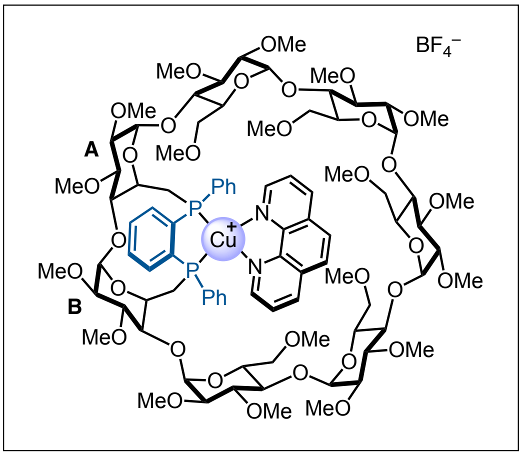

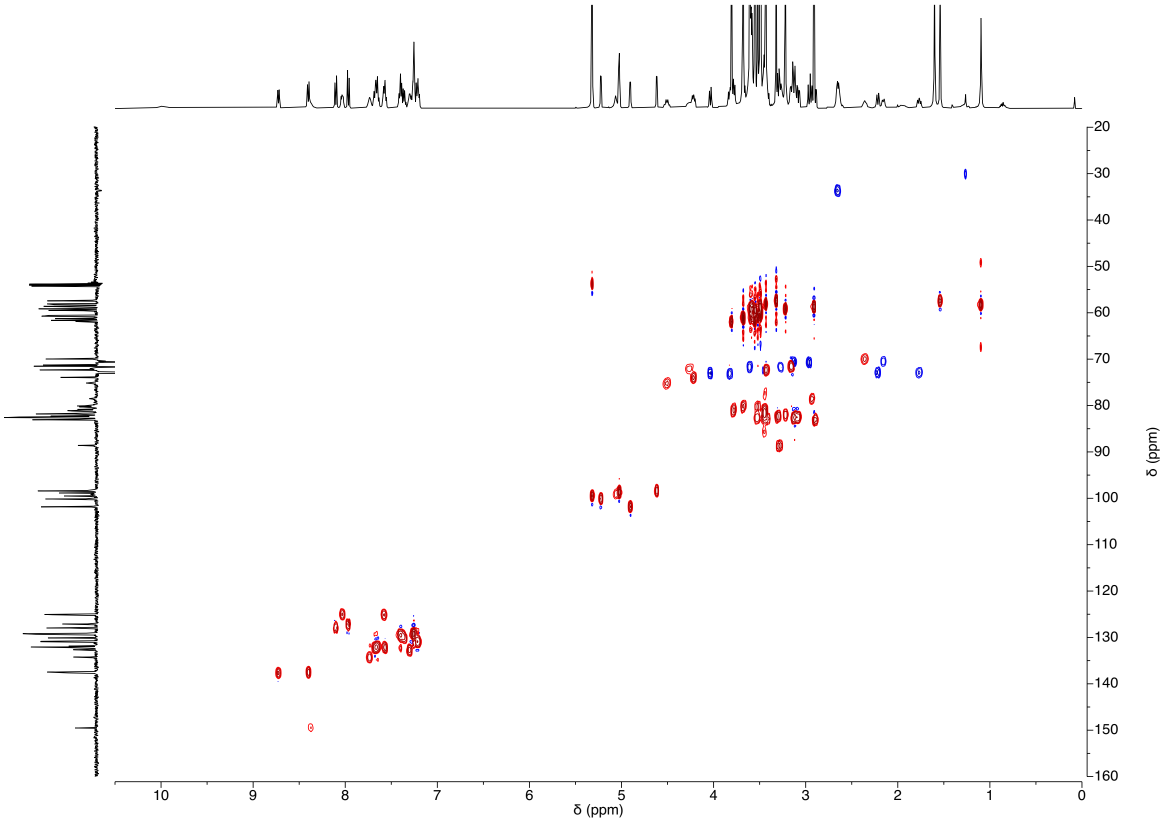

Figure S4i. $^1H/^{13}C\{^1H\}$ HSQC NMR (500 MHz, CD_2Cl_2) spectrum of compound **5**.

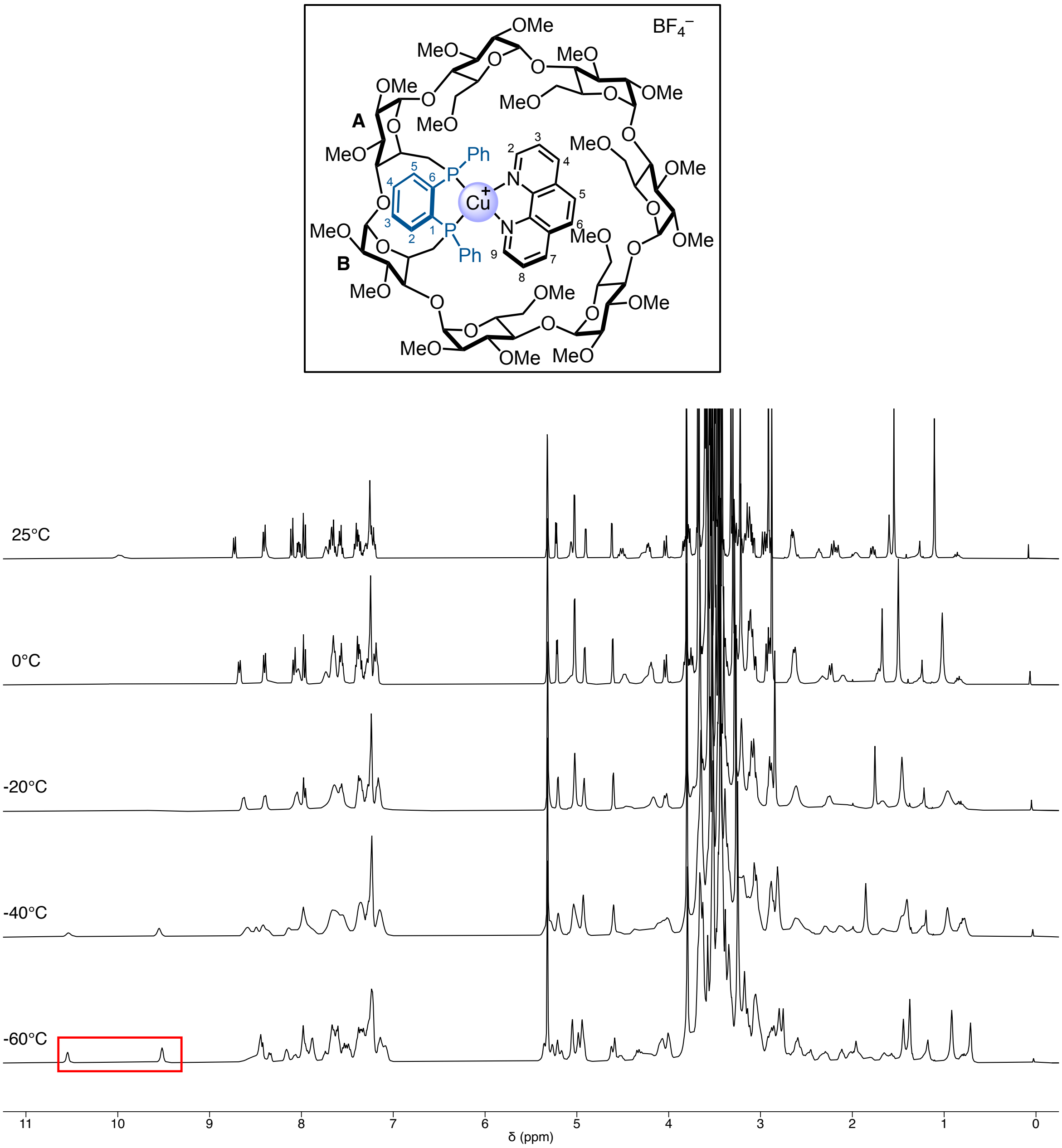

Figure S4j. ^{1}H NMR (400 MHz, CD_2Cl_2) spectra of compound **5** recorded at low temperatures temperatures showing the phen H-9 signals corresponding to the two conformers of **6** (red rectangle).

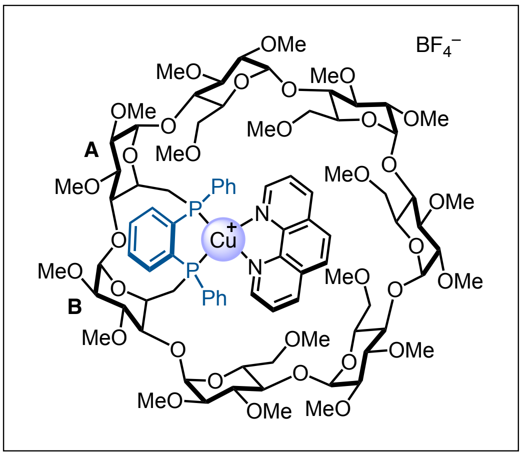

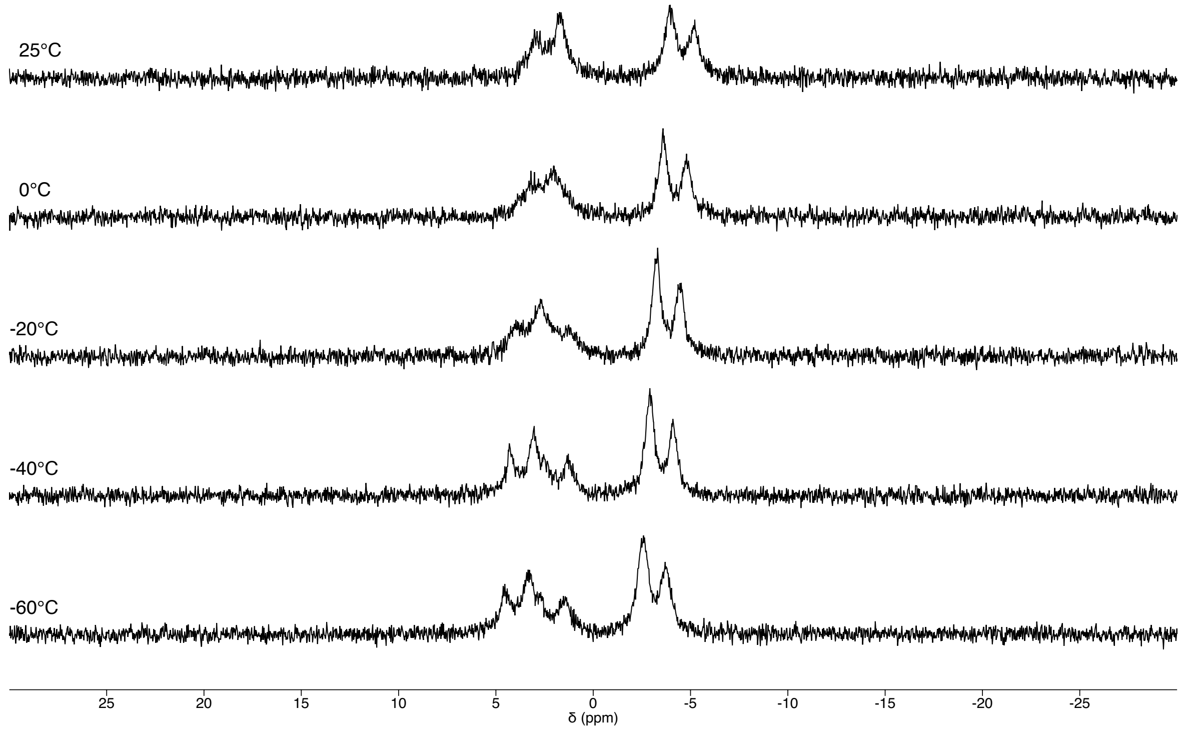

Figure S4k. $^{31}P\{^{1}H\}$ NMR (162 MHz, CD_2Cl_2) spectra of compound **5** recorded at low temperatures.

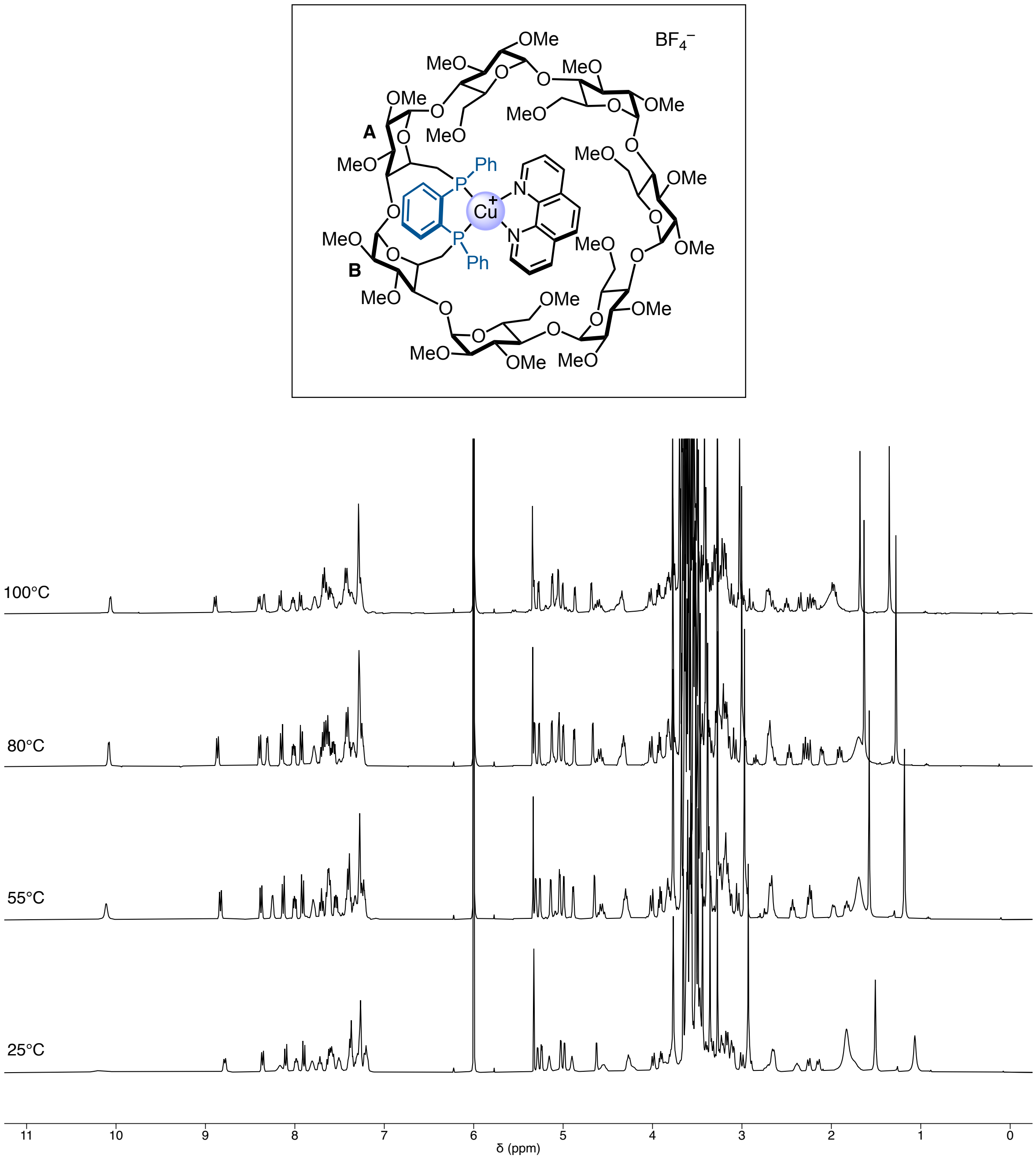

Figure S4l. ^{1}H NMR (400 MHz, $C_2D_2Cl_4$) spectra of compound **5** recorded at high temperatures.

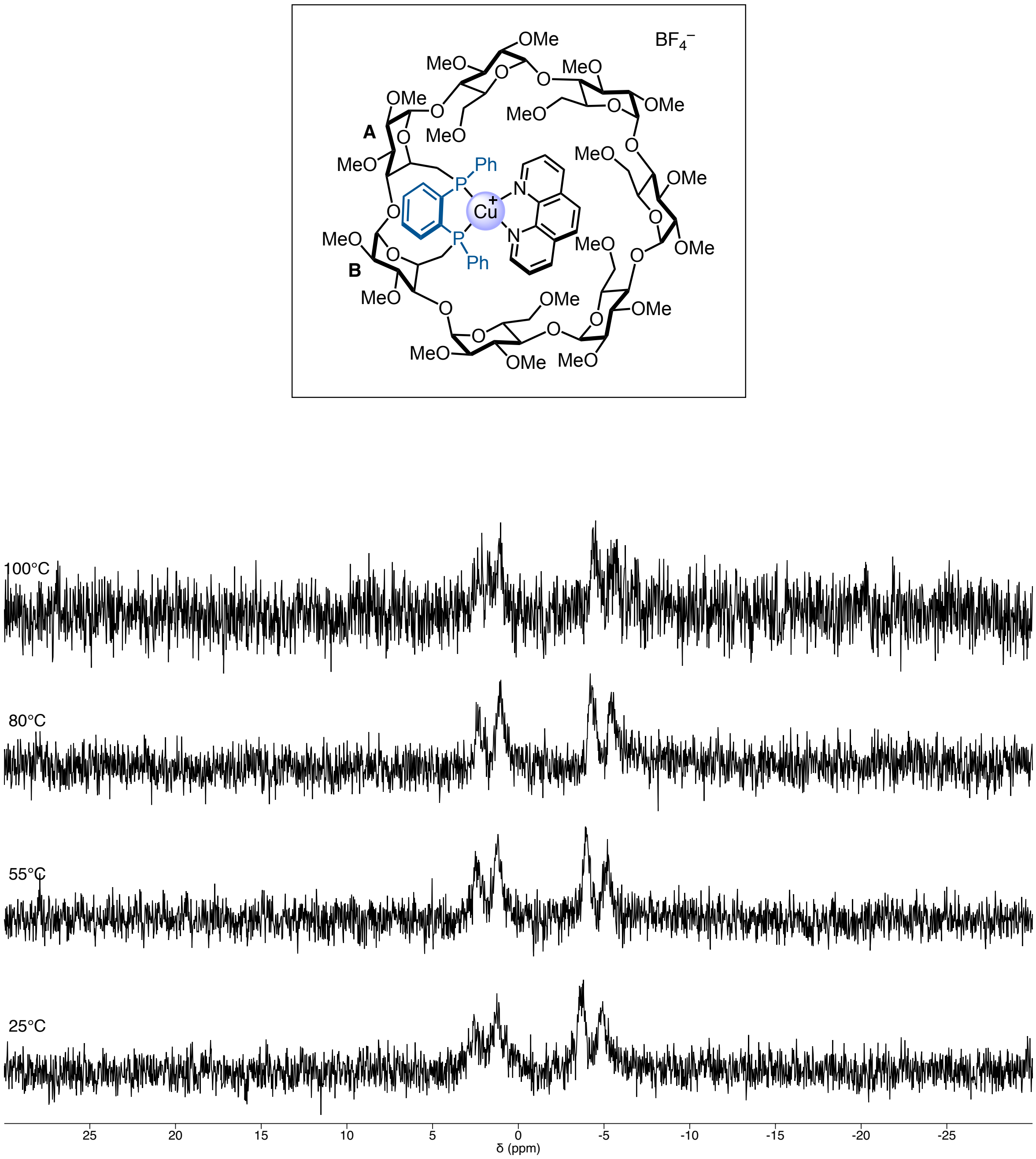

Figure S4m. $^{31}P\{^{1}H\}$ NMR (162 MHz, $C_2D_2Cl_4$) spectra of compound **5** recorded at high temperatures.

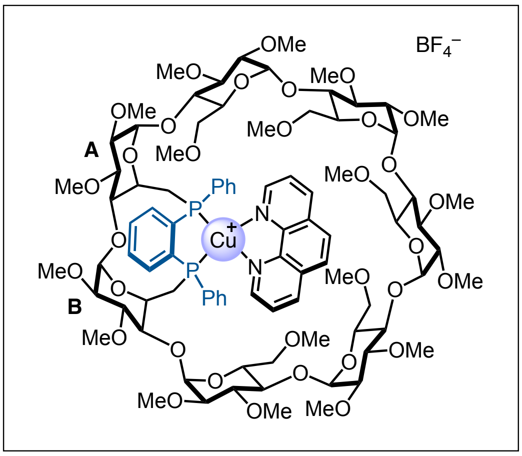

Acquisition Parameter

Source Type	ESI	Capillary	4500 V	Nebulizer	0.3 Bar	Set Hexapole RF	330.0 Vpp
Ion Polarity	Positive	Dry Heater	200 °C	Dry Gas	3.0 l/min	Set Capillary Exit	150.0 V

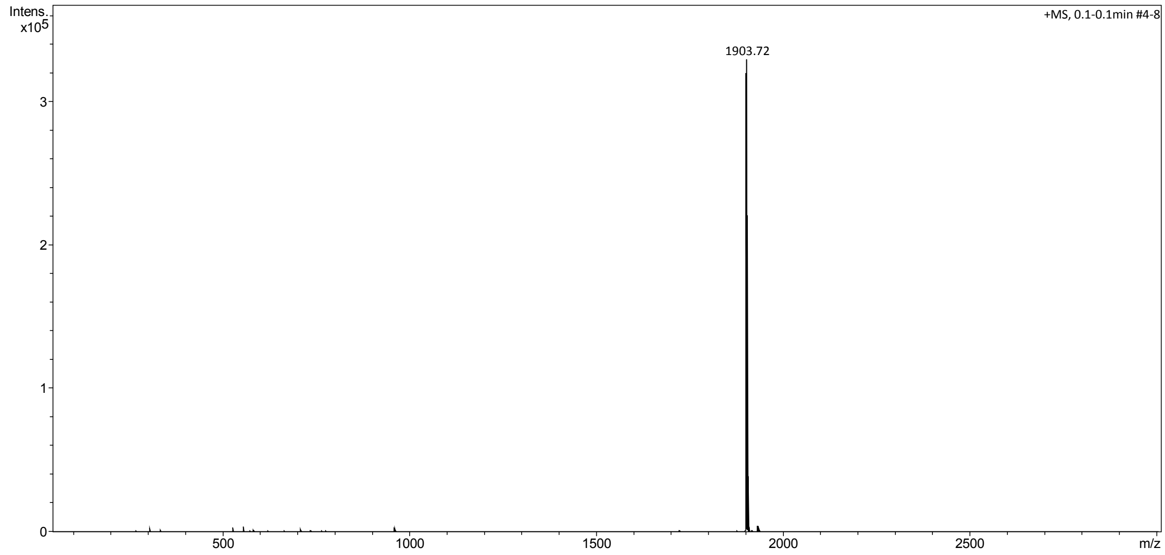

Figure S4n. ESI-TOF mass spectrum of compound **5**.

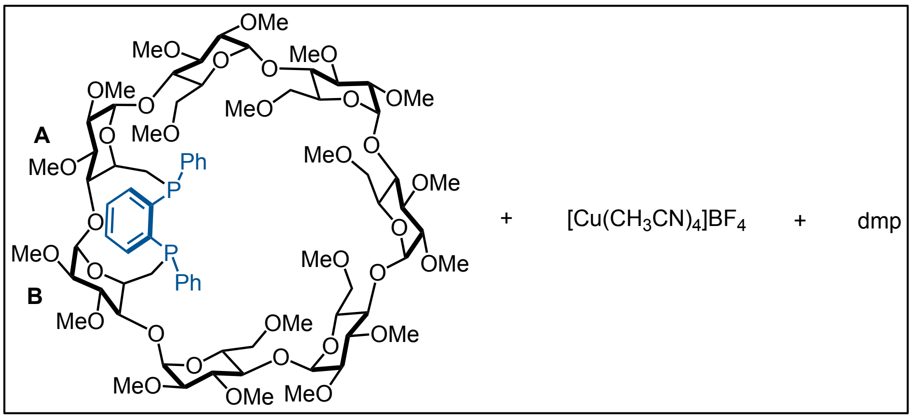

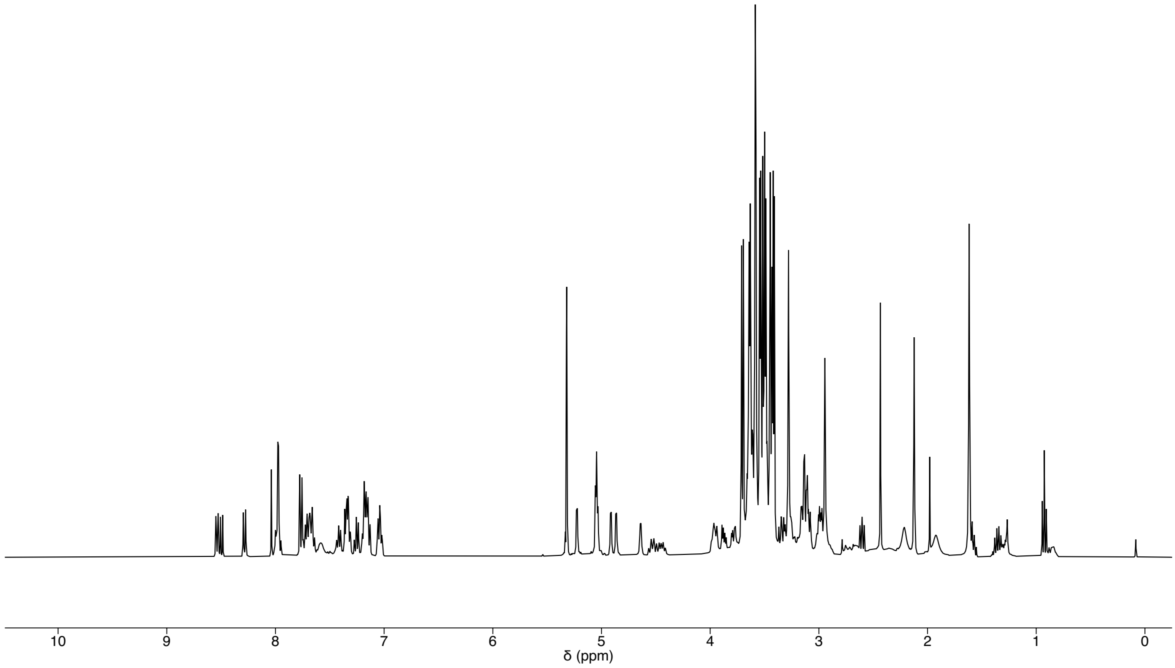

Figure S5a. ^{1}H NMR (400 MHz, CD_2Cl_2) spectrum of the crude mixture obtained from **4c**, $[Cu(CH_3CN)_4]BF_4$ and dmp.

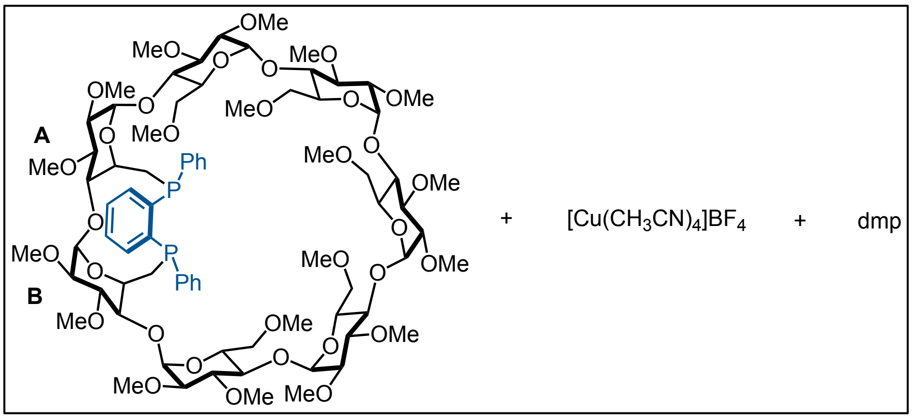

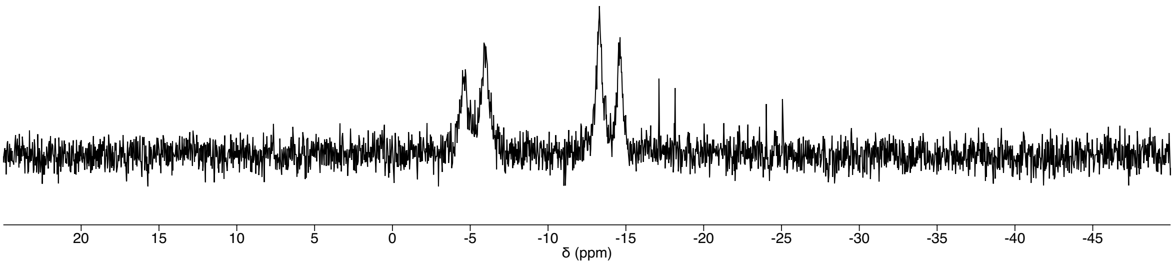

Figure S5b. $^{31}P\{^1H\}$ NMR (162 MHz, CD_2Cl_2) spectrum of the crude mixture obtained from **4c**, $[Cu(CH_3CN)_4]BF_4$ and dmp.

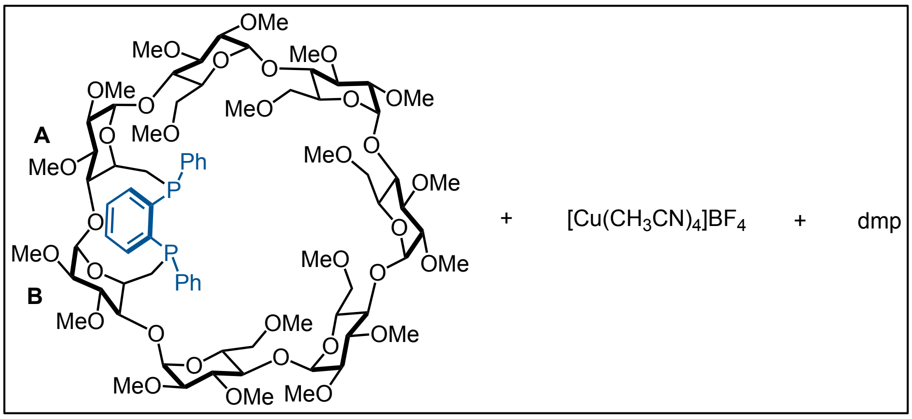

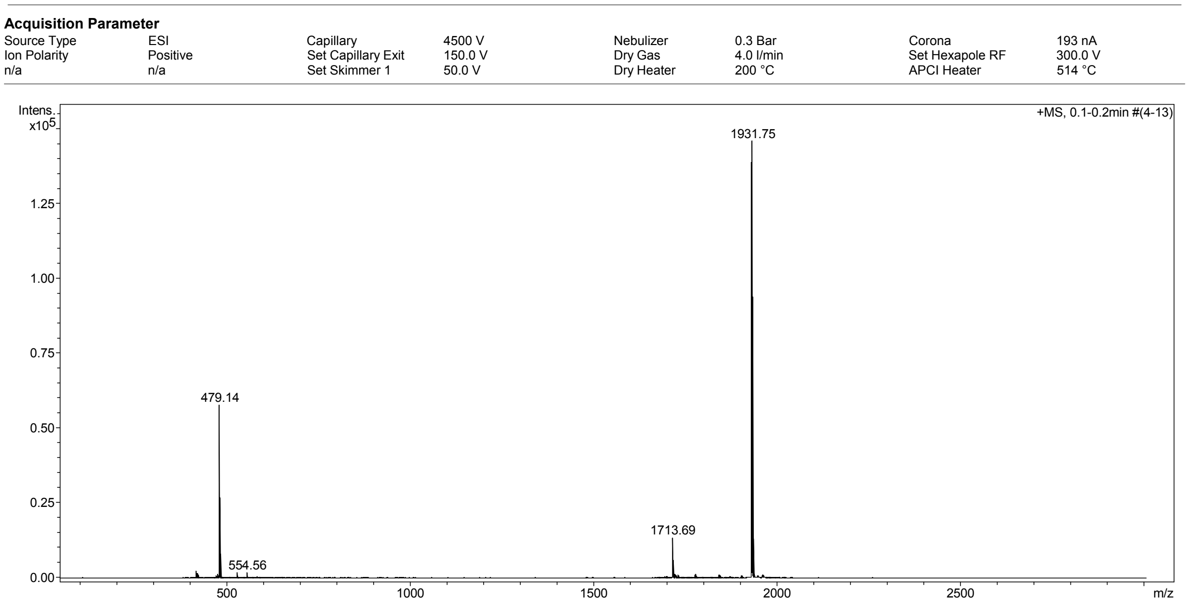

Figure S5c. ESI-TOF mass spectrum of the crude mixture obtained from **4c**, $[Cu(CH_3CN)_4]BF_4$ and dmp revealing the presence of the $[Cu(dmp)_2]^+$ (m/z calcd for $[Cu(dmp)_2]^+$ = 479.13) and [Cu(**4c**)(dmp)]$^+$ (m/z calcd for [Cu(**4c**)(dmp)]$^+$ = 1931.55) cations.

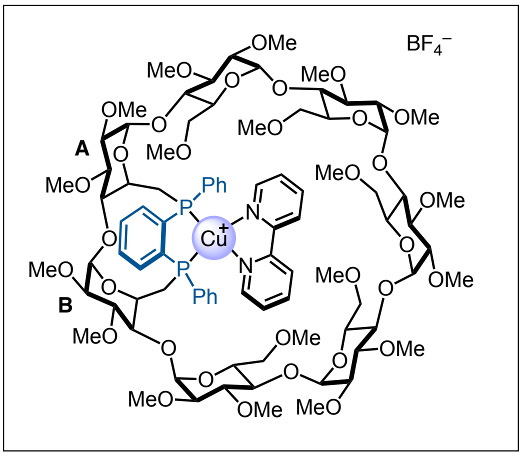

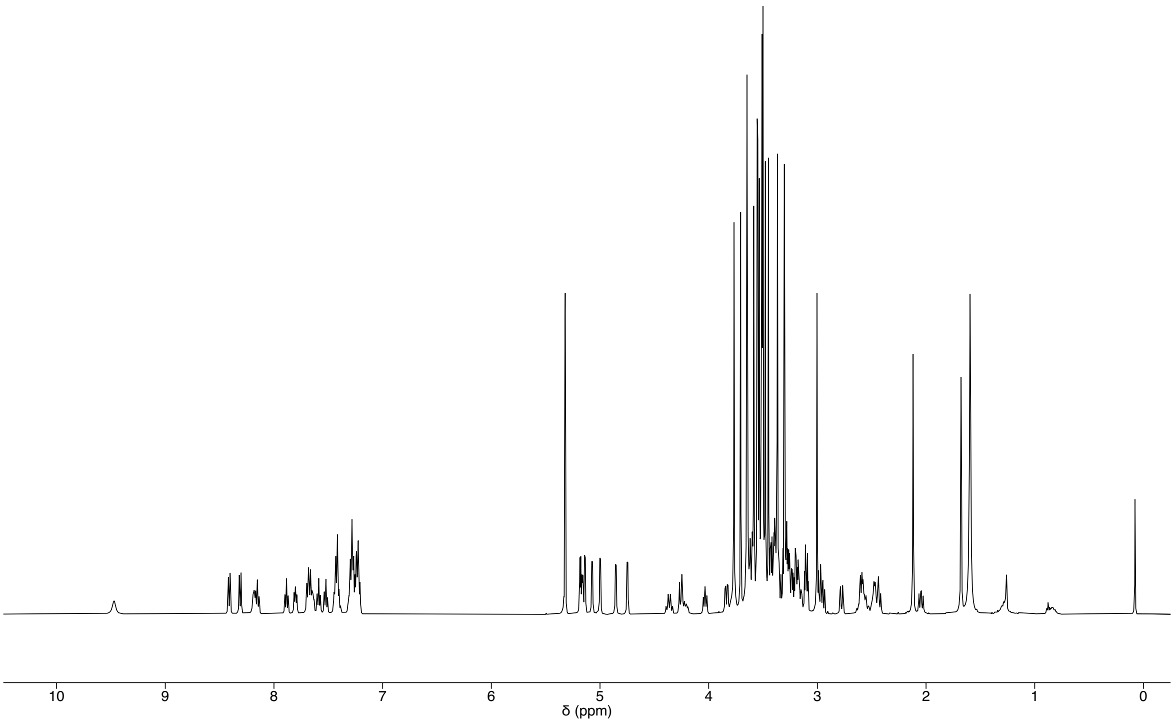

Figure S6a. ^{1}H NMR (500 MHz, CD_2Cl_2) spectrum of compound **6**.

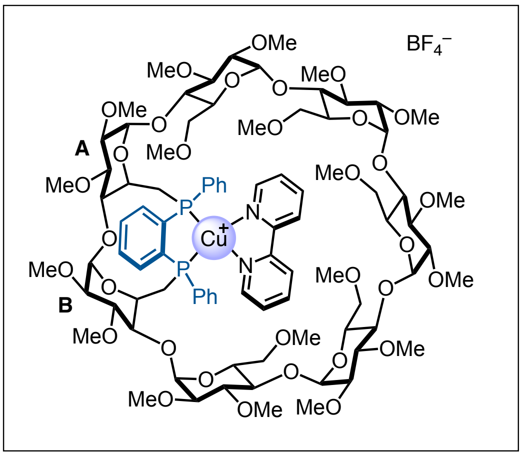

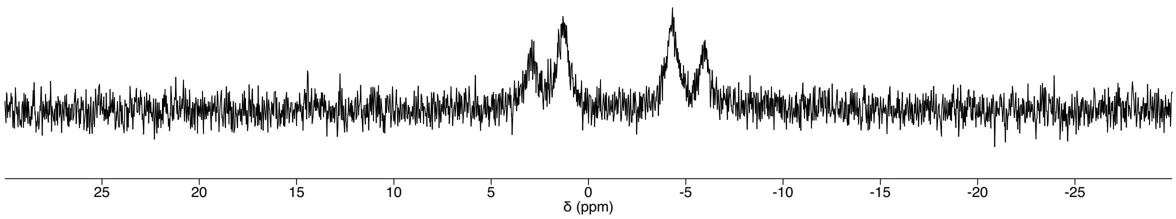

Figure S6b. $^{31}P\{^{1}H\}$ NMR (121.5 MHz, CD_2Cl_2) spectrum of compound **6**.

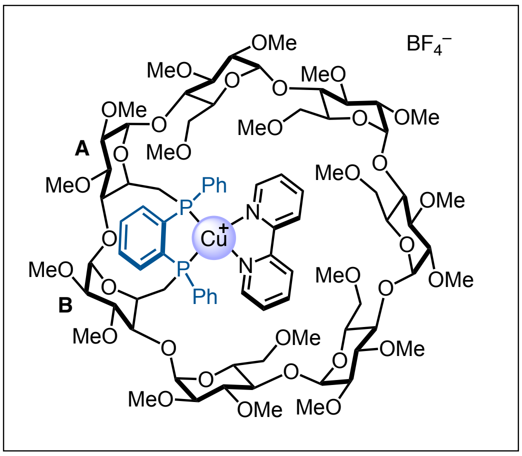

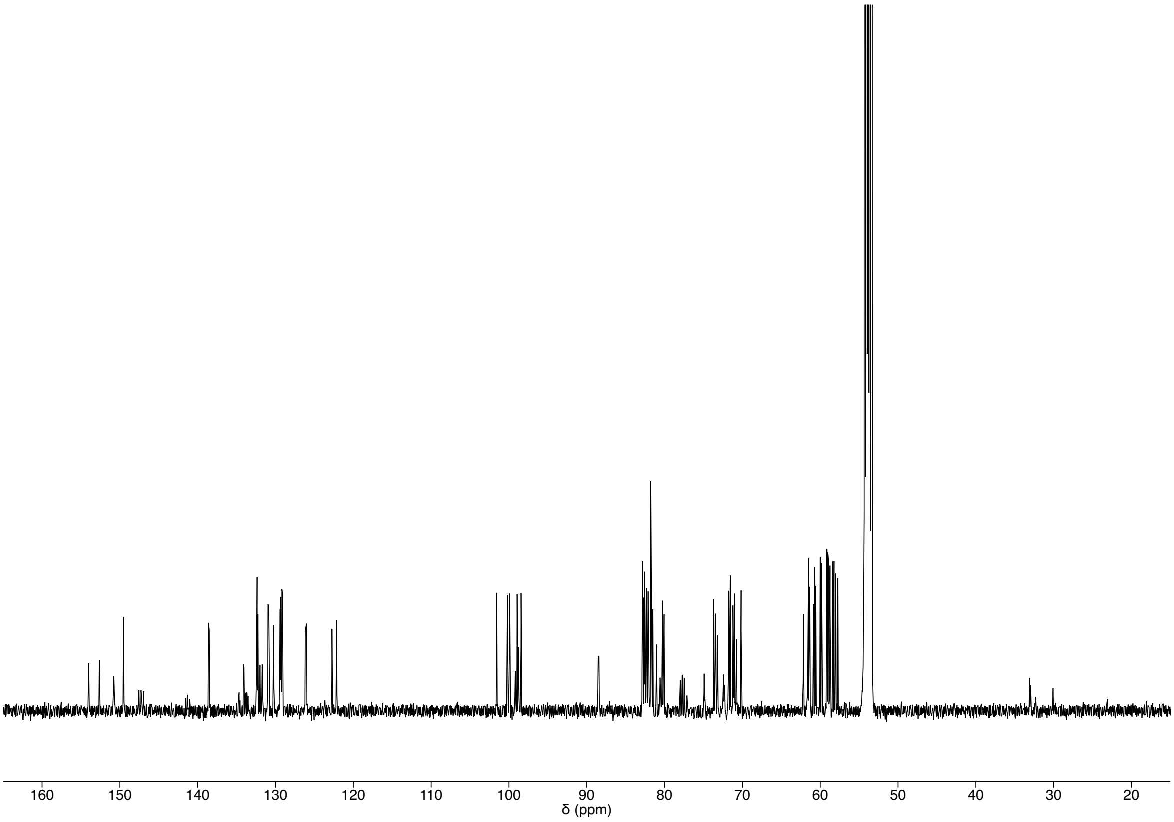

Figure S6c. $^{13}C\{^1H\}$ NMR (126 MHz, CD_2Cl_2) spectrum of compound **6**.

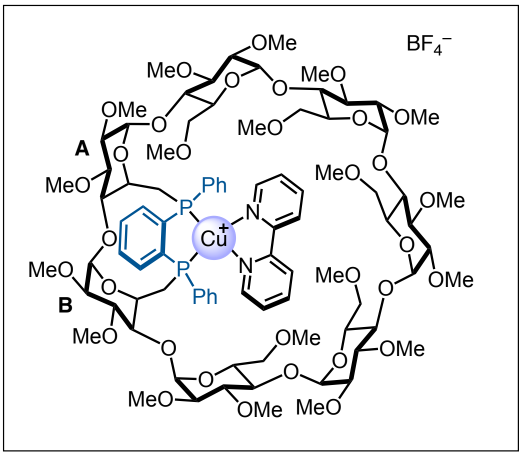

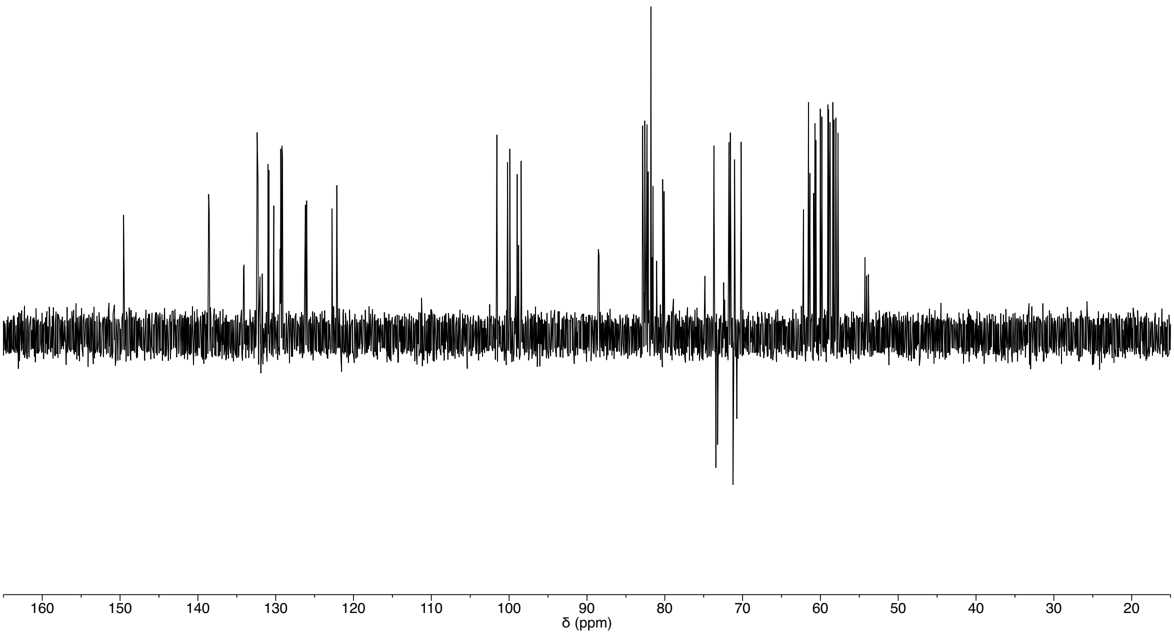

Figure S6d. DEPT 135 NMR (126 MHz, CD_2Cl_2) spectrum of compound **6**.

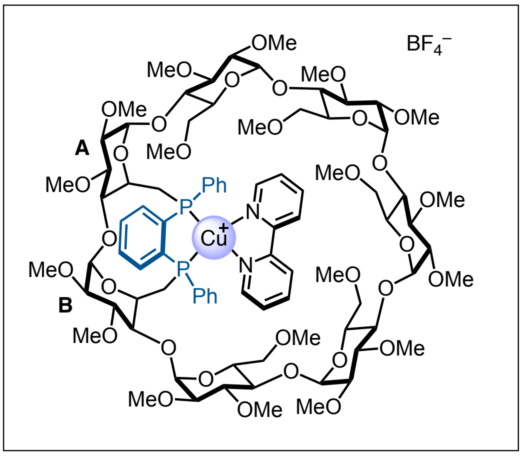

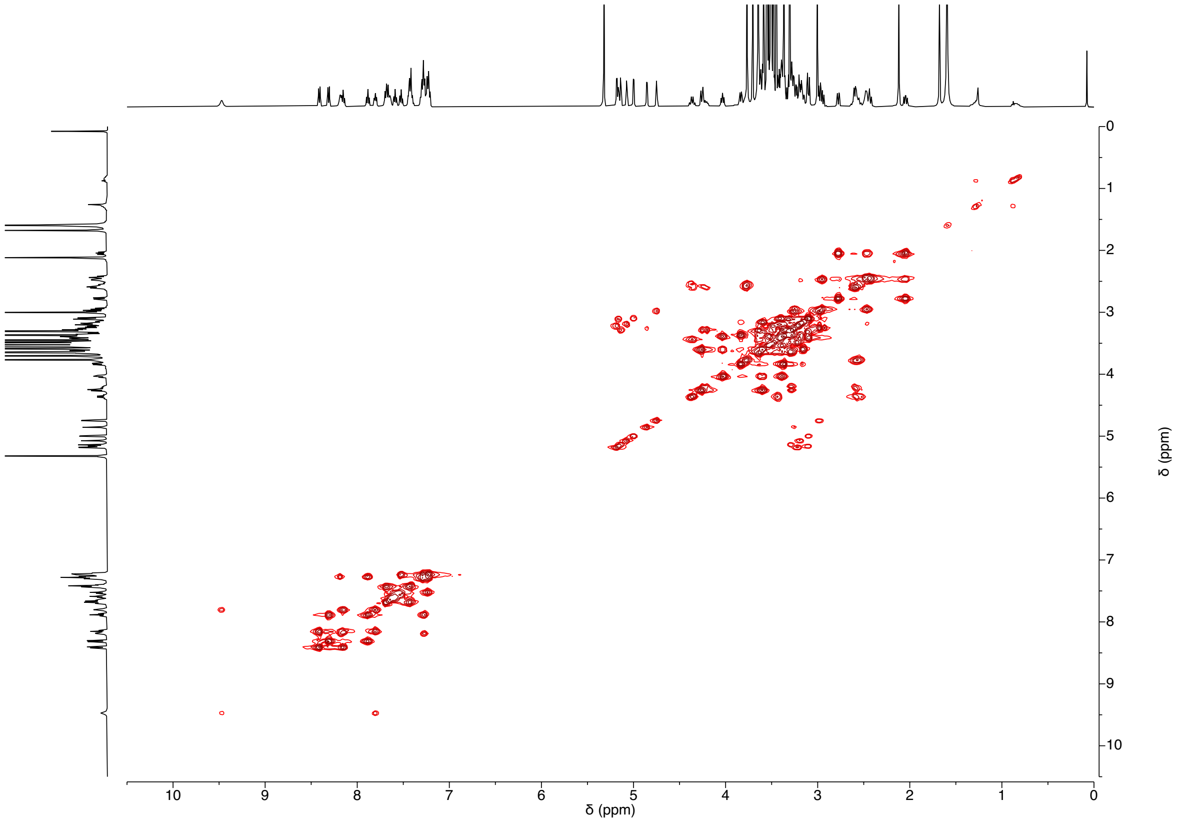

Figure S6e. ^{1}H/^{1}H COSY NMR (500 MHz, CD_2Cl_2) spectrum of compound **6**.

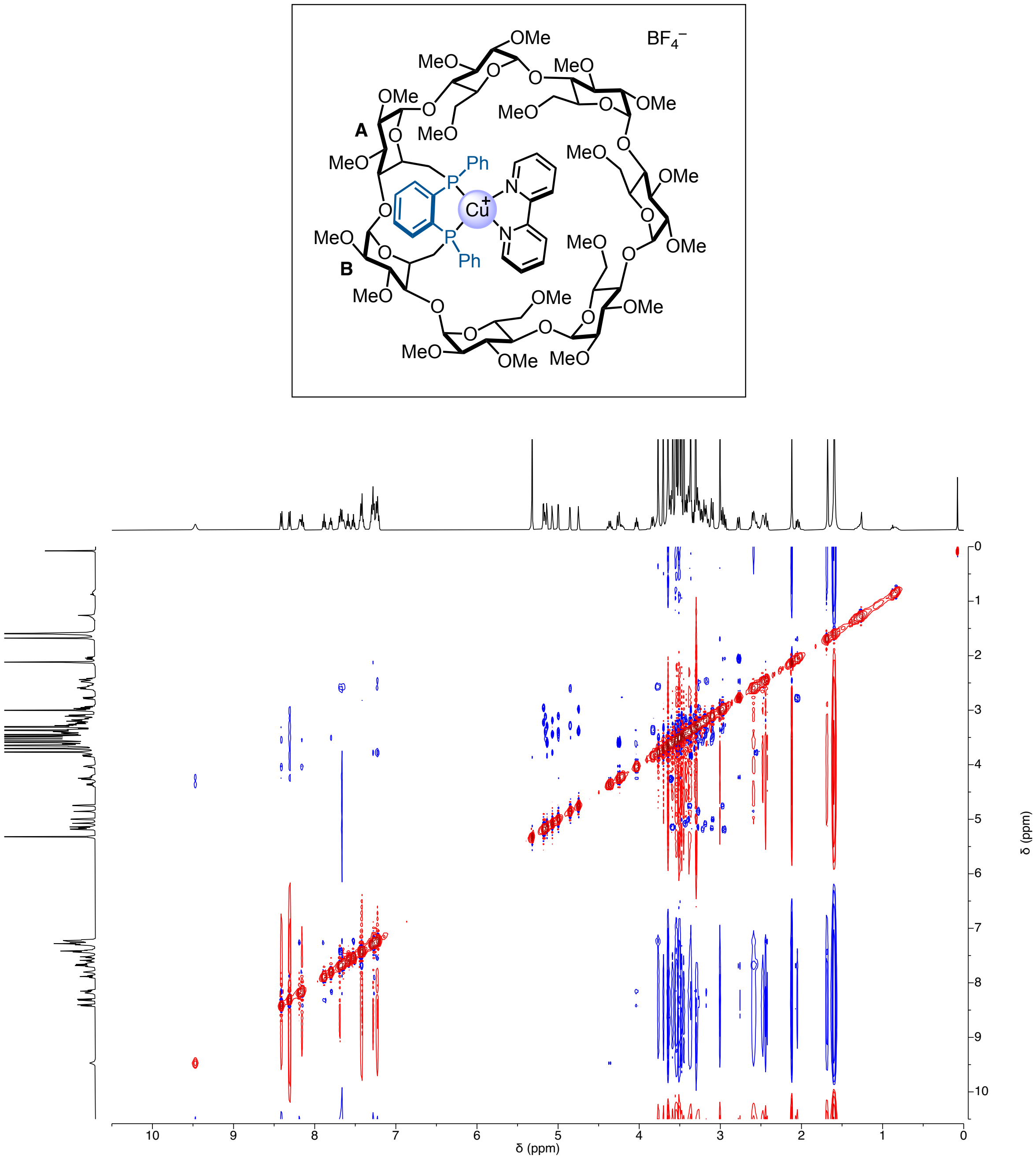

Figure S6f. $^1H/^1H$ ROESY NMR (500 MHz, CD_2Cl_2) spectrum of compound **6**.

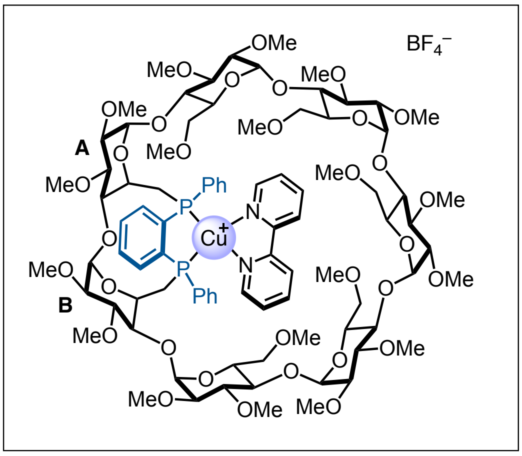

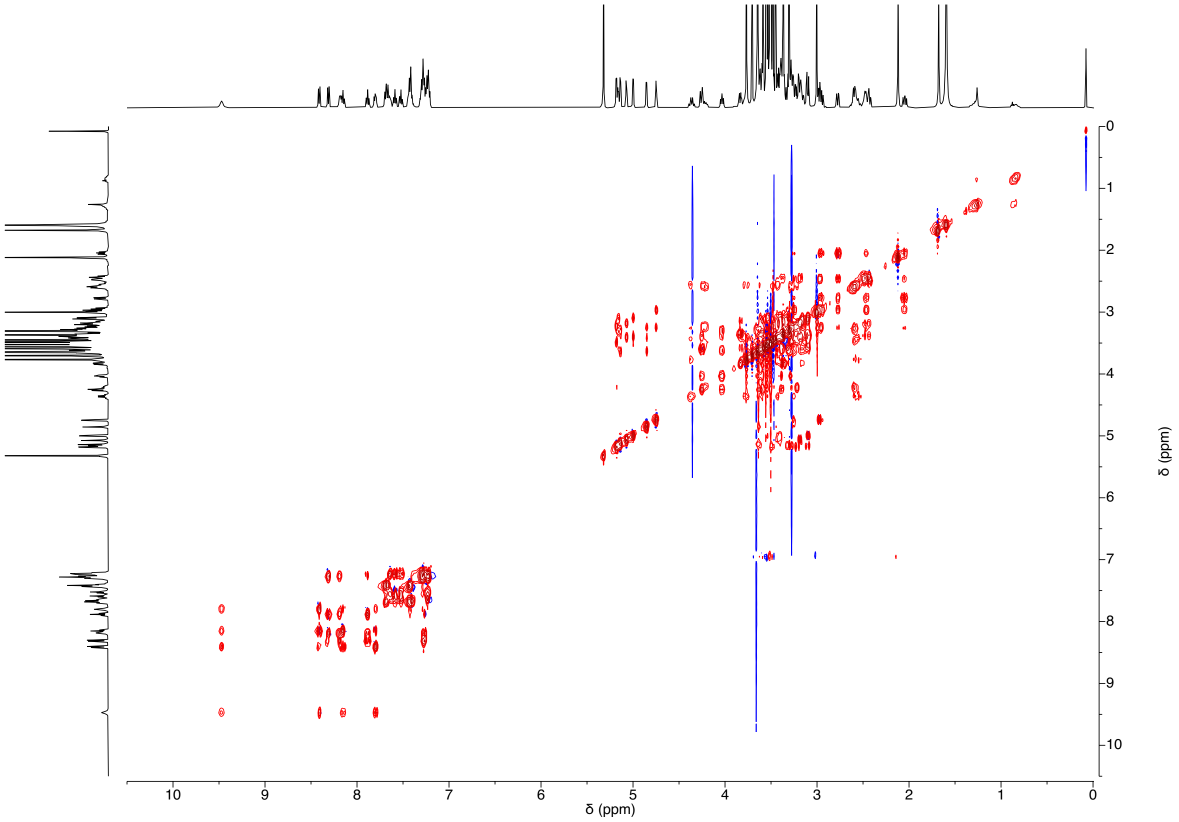

Figure S6g. $^{1}H/^{1}H$ TOCSY NMR (500 MHz, CD_2Cl_2) spectrum of compound **6**.

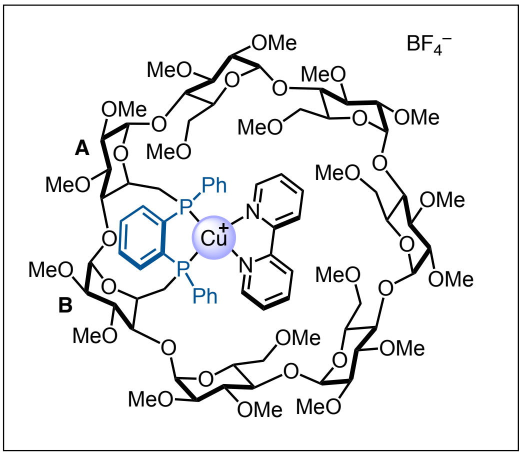

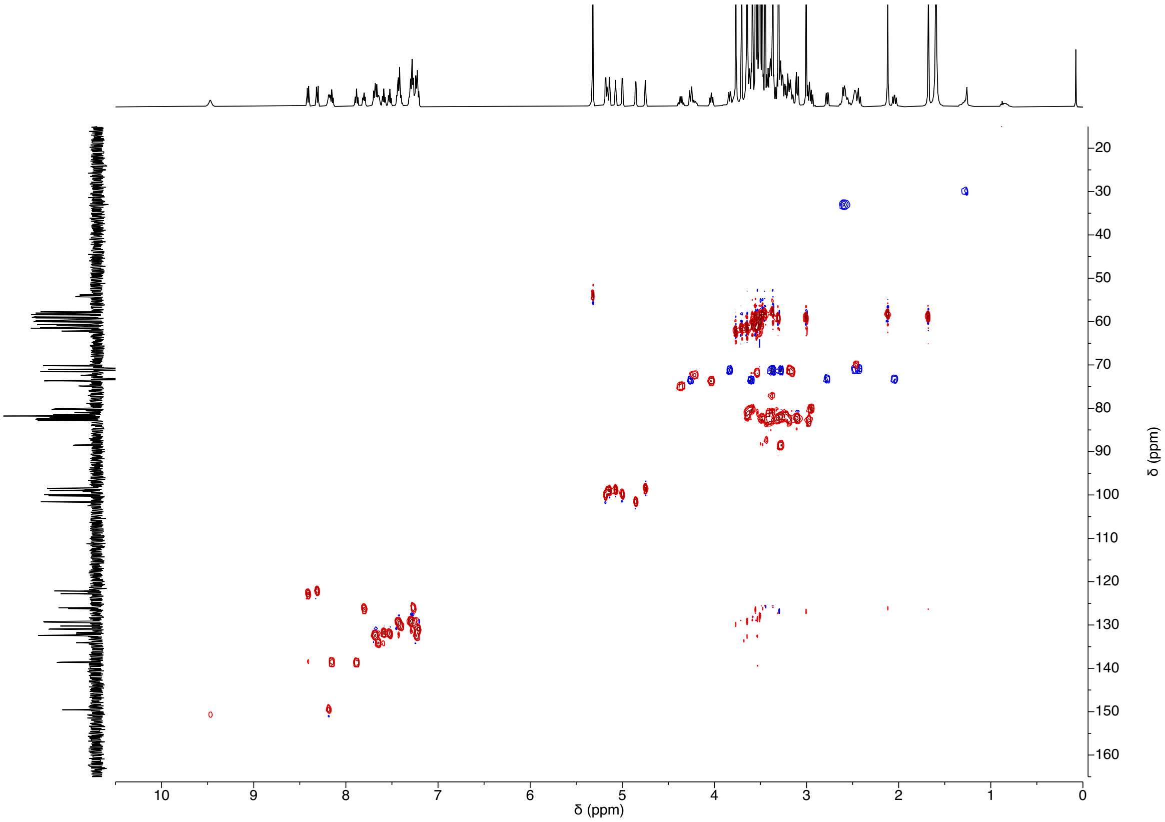

Figure S6h. $^{1}H/^{13}C\{^{1}H\}$ HSQC NMR (500 MHz, CD_2Cl_2) spectrum of compound **6**.

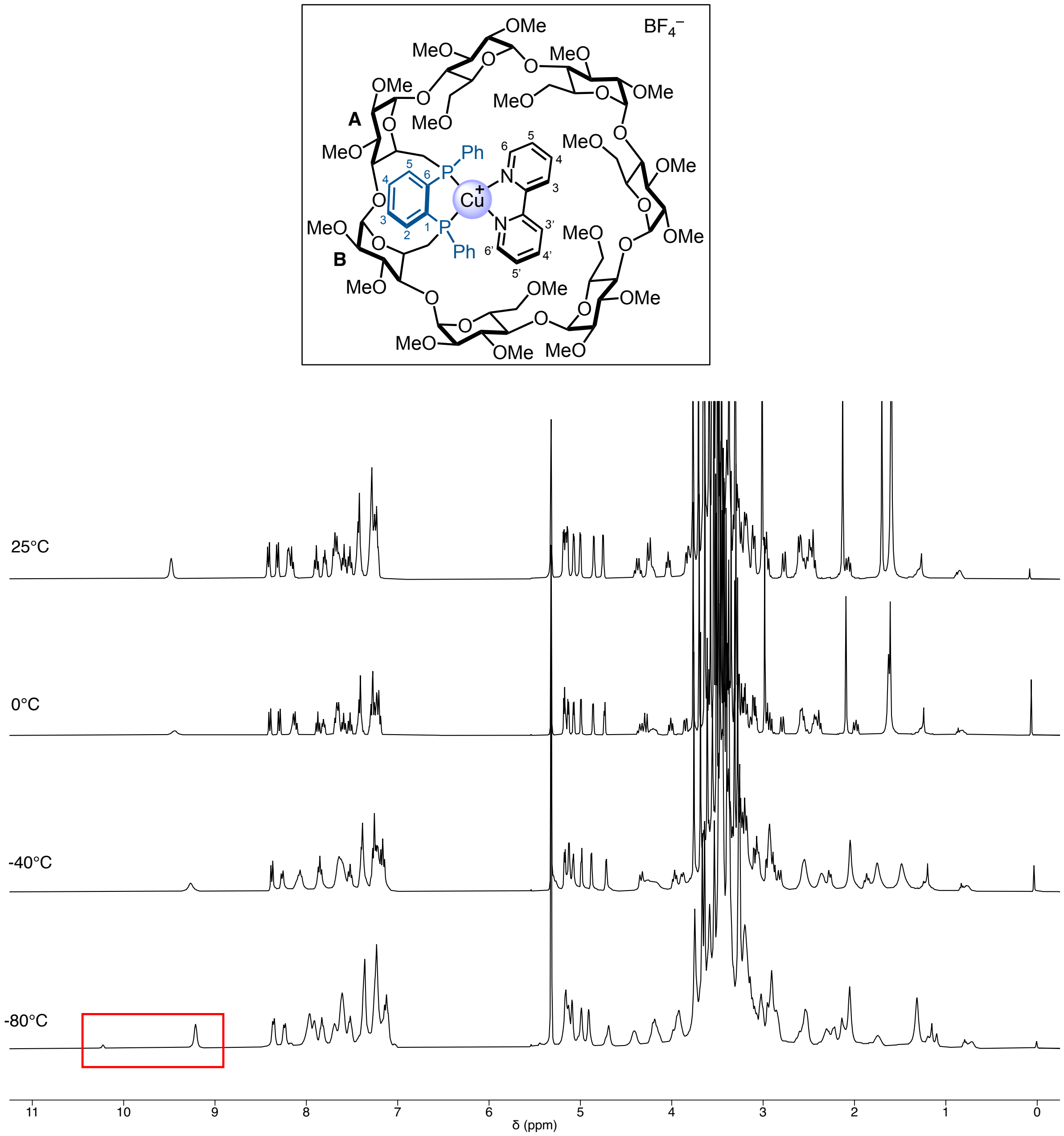

Figure S6i. ^{1}H NMR (400 MHz, CD_2Cl_2) spectra of compound **6** recorded at low temperatures showing the bpy H-6' signals corresponding to the two conformers of **6** (red rectangle).

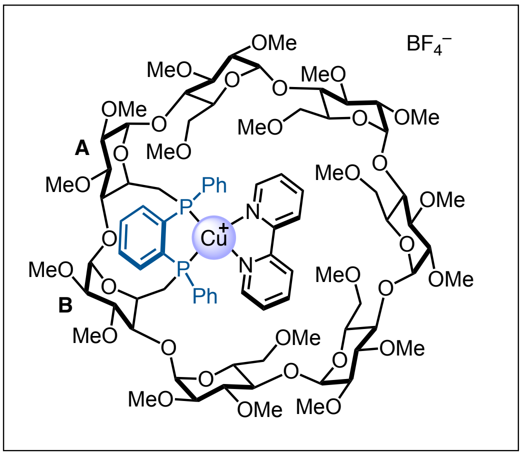

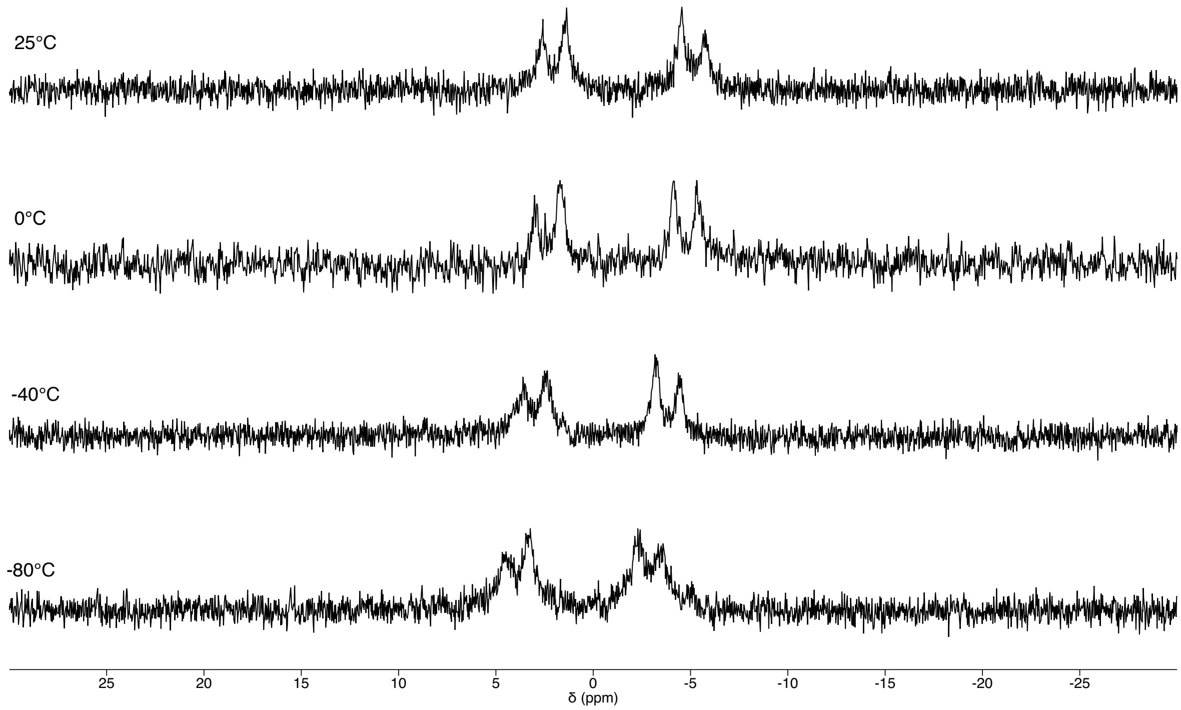

Figure S6j. $^{31}P\{^{1}H\}$ NMR (162 MHz, CD_2Cl_2) spectra of compound **6** recorded at low temperatures.

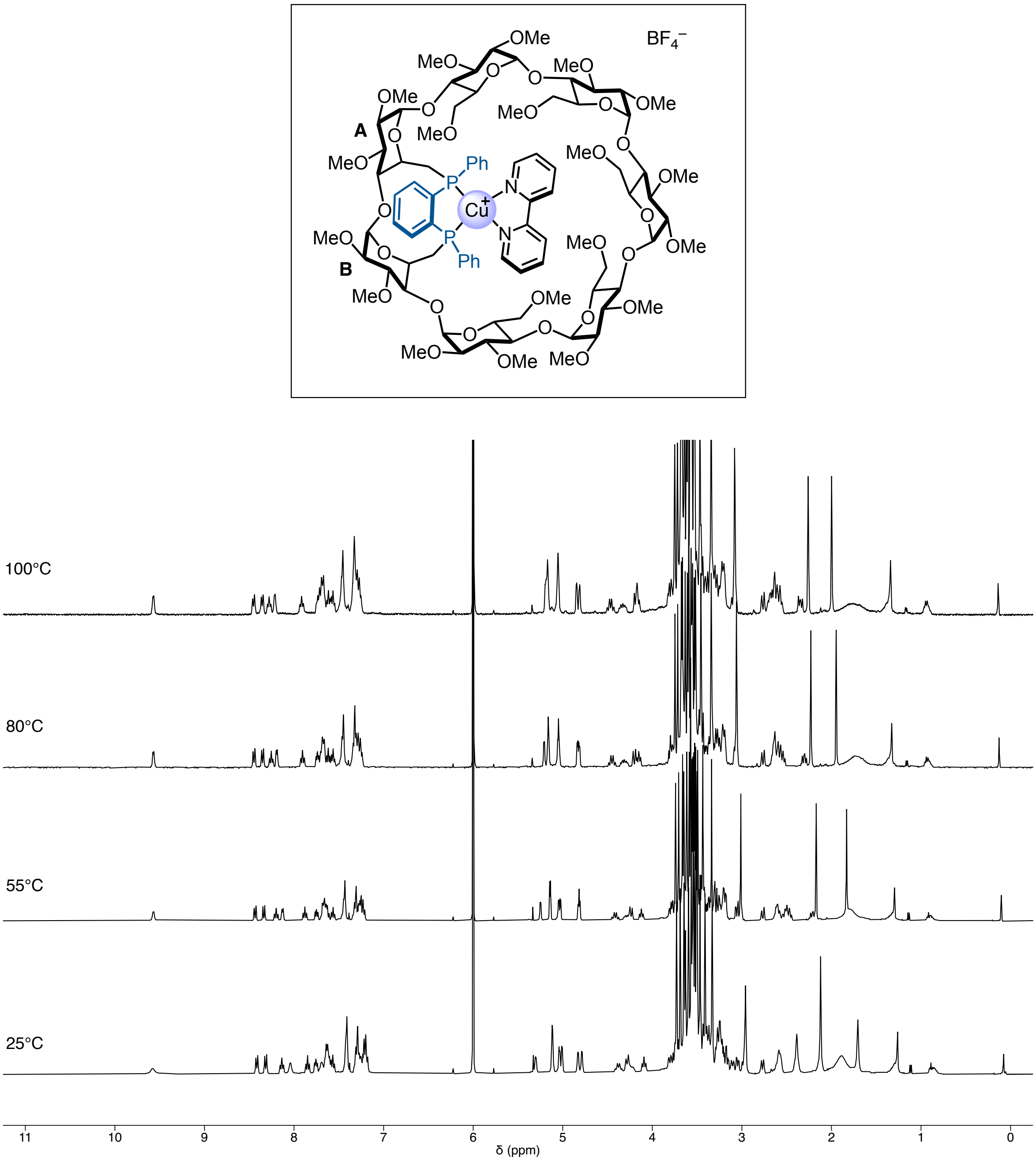

Figure S6k. ^{1}H NMR (400 MHz, $C_2D_2Cl_4$) spectra of compound **6** recorded at high temperatures.

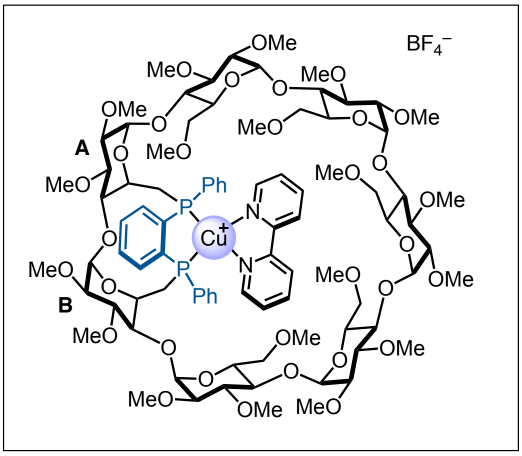

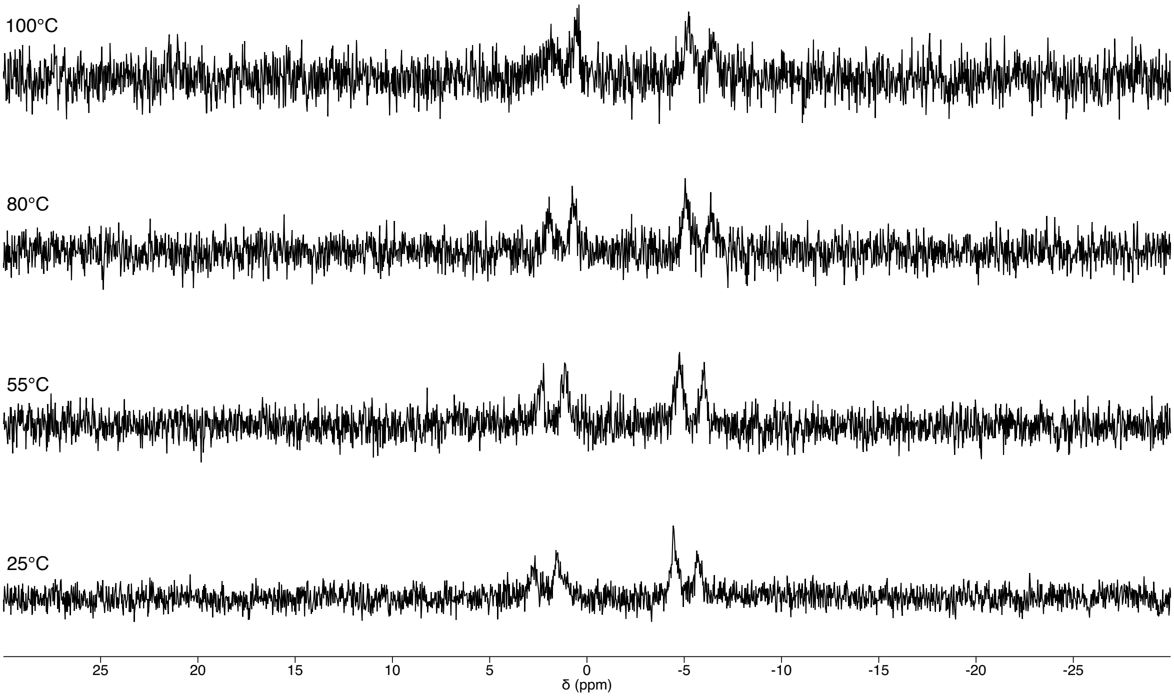

Figure S6I. $^{31}P\{^{1}H\}$ NMR (162 MHz, $C_2D_2Cl_4$) spectra of compound **6** recorded at high temperatures.

.

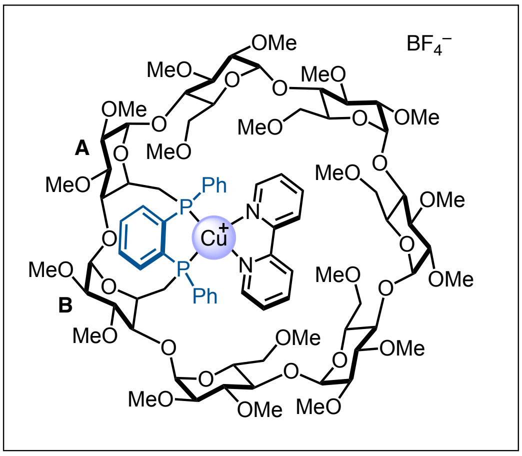

Acquisition Parameter

Source Type	ESI	Capillary	4500 V	Nebulizer	0.3 Bar	Corona	172 nA
Ion Polarity	Positive	Set Capillary Exit	150.0 V	Dry Gas	4.0 l/min	Set Hexapole RF	300.0 V
n/a	n/a	Set Skimmer 1	50.0 V	Dry Heater	200 °C	APCI Heater	514 °C

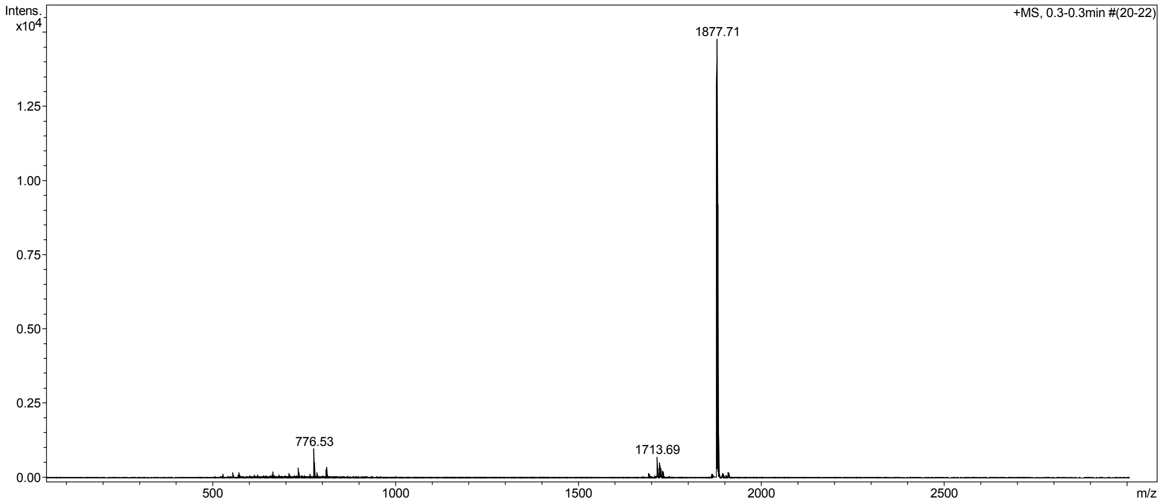

Figure S6m. ESI-TOF mass spectrum of compound **6**.

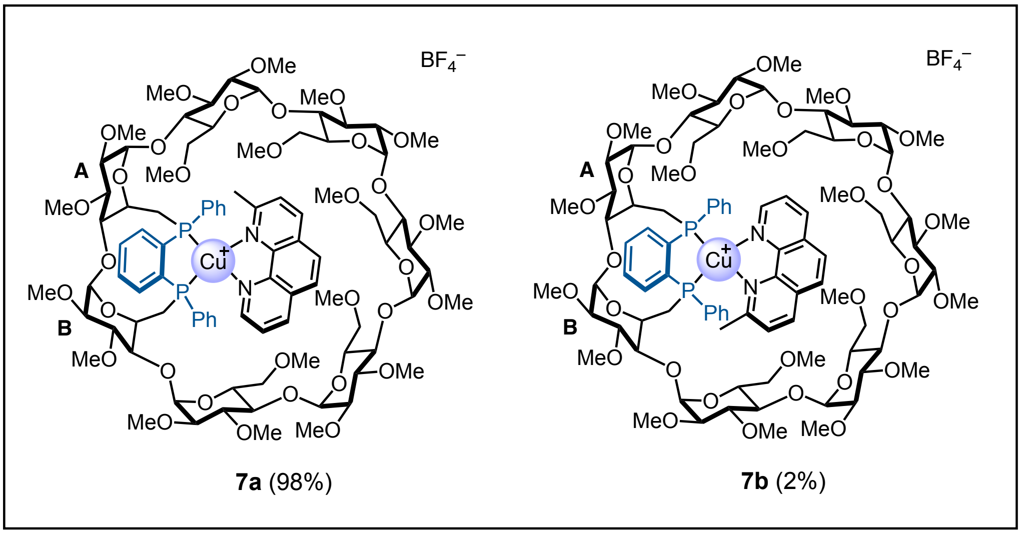

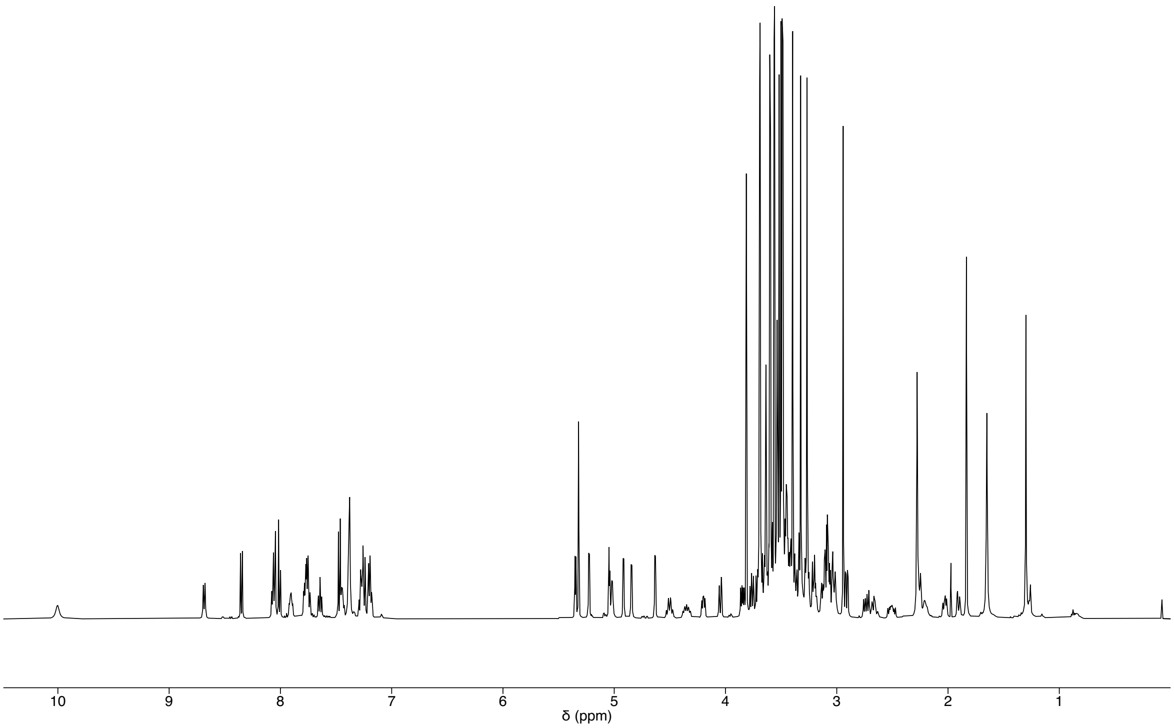

Figure S7a. ^{1}H NMR (500 MHz, CD_2Cl_2) spectrum of compounds **7a,b**.

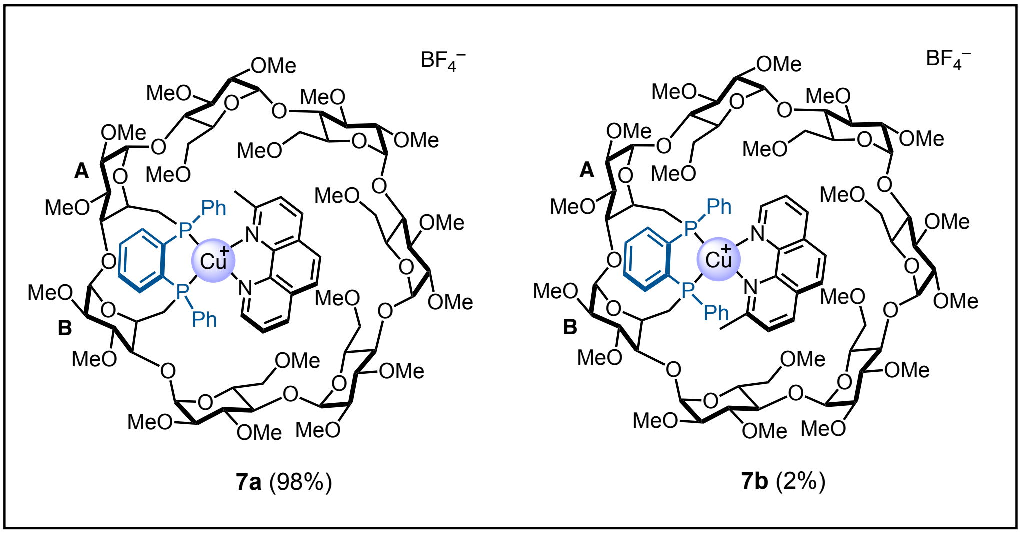

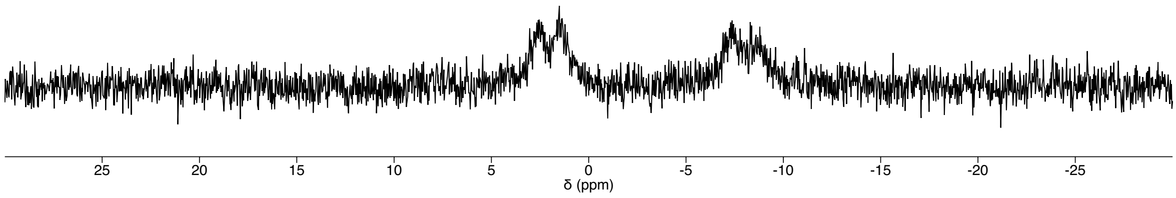

Figure S7b. $^{31}P\{^{1}H\}$ NMR (202.5 MHz, CD_2Cl_2) spectrum of compounds **7a,b**.

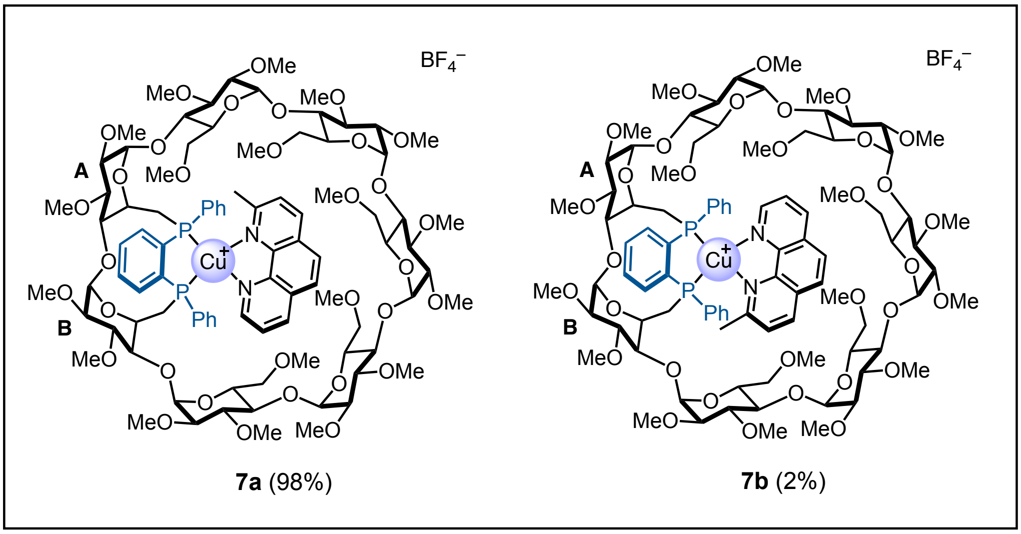

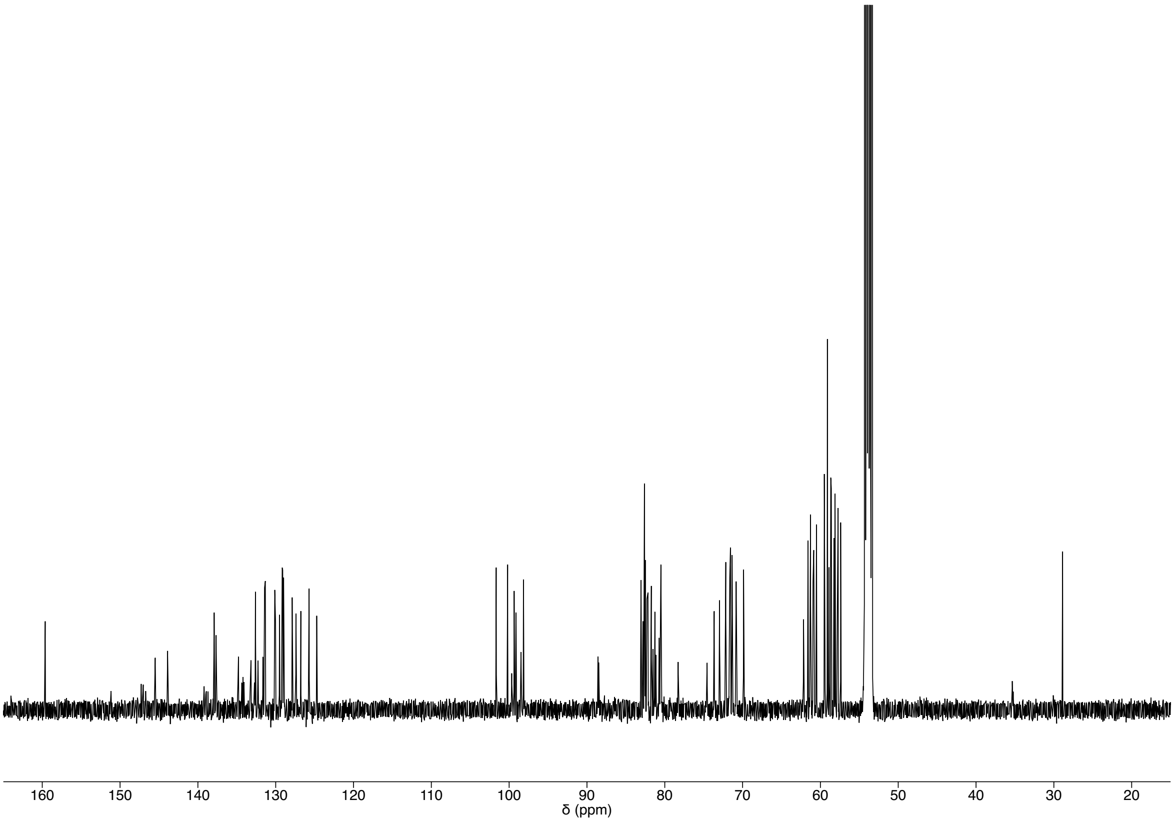

Figure S7c. $^{13}C\{^{1}H\}$ NMR (126 MHz, CD_2Cl_2) spectrum of compounds **7a,b**.

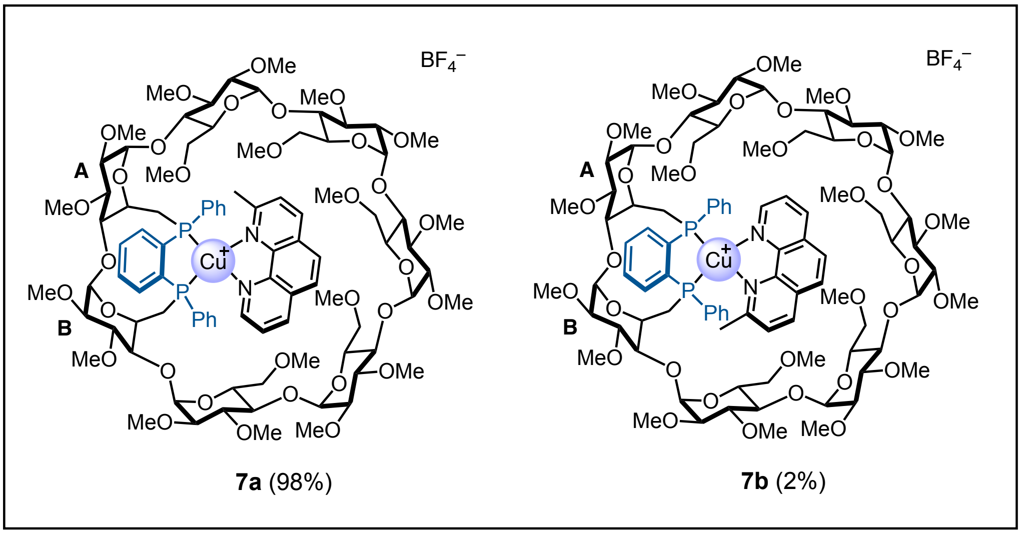

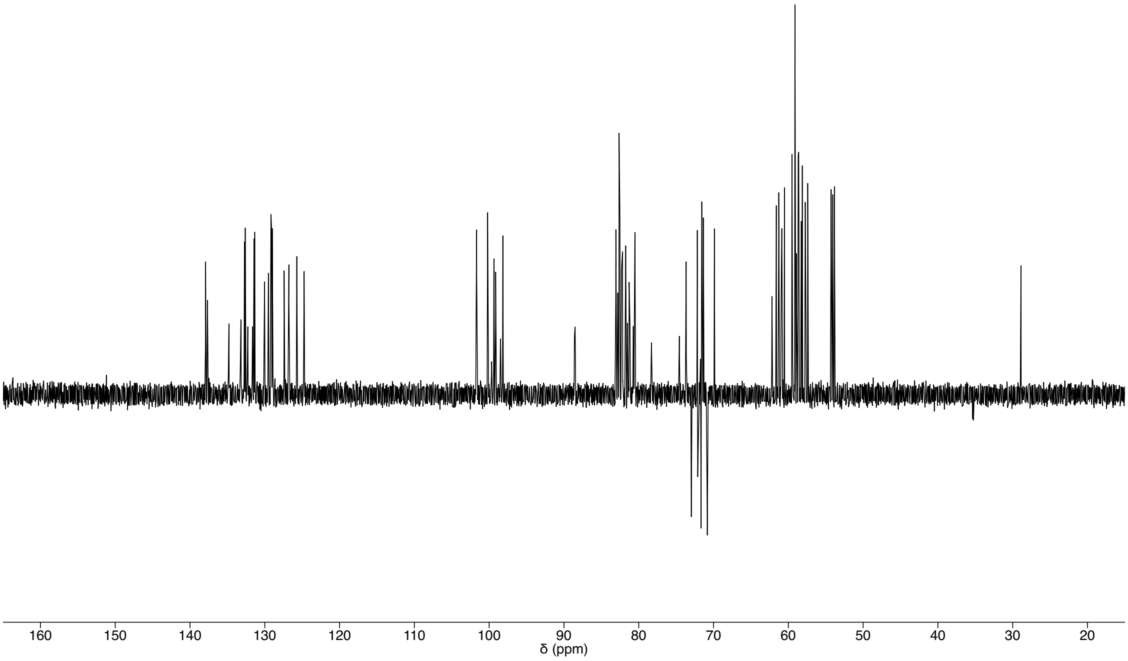

Figure S7d. DEPT 135 NMR (126 MHz, CD_2Cl_2) spectrum of compounds **7a,b**.

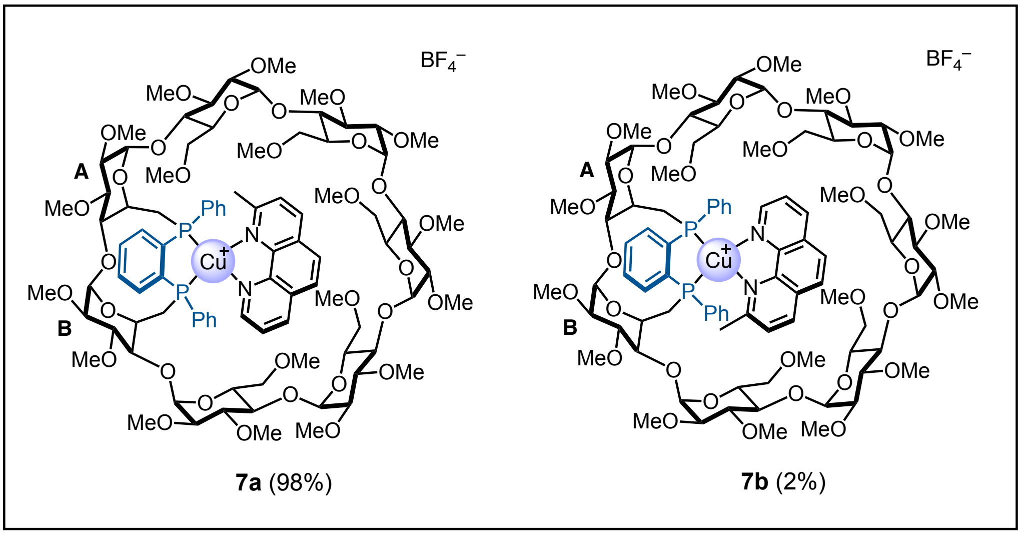

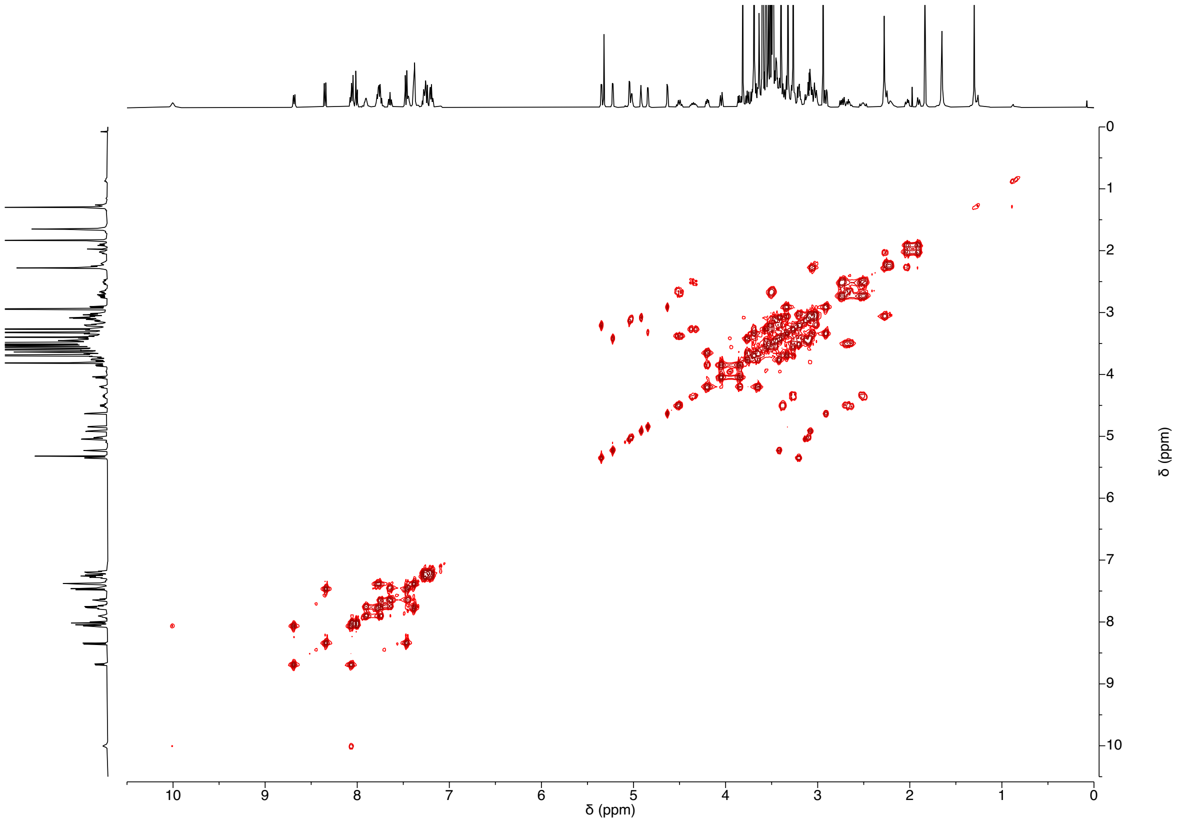

Figure S7e. ^{1}H/^{1}H COSY NMR (500 MHz, CD_2Cl_2) spectrum of compounds **7a,b**.

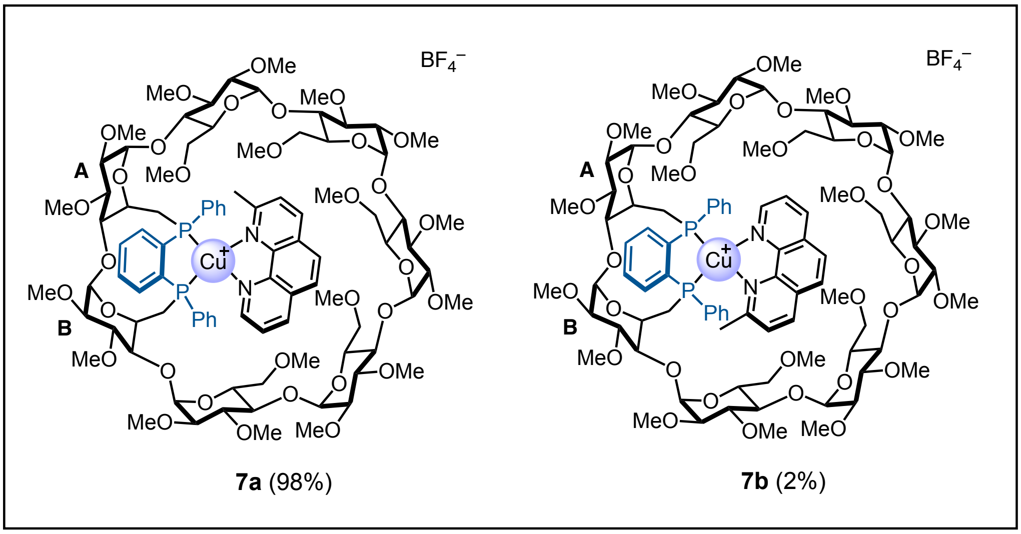

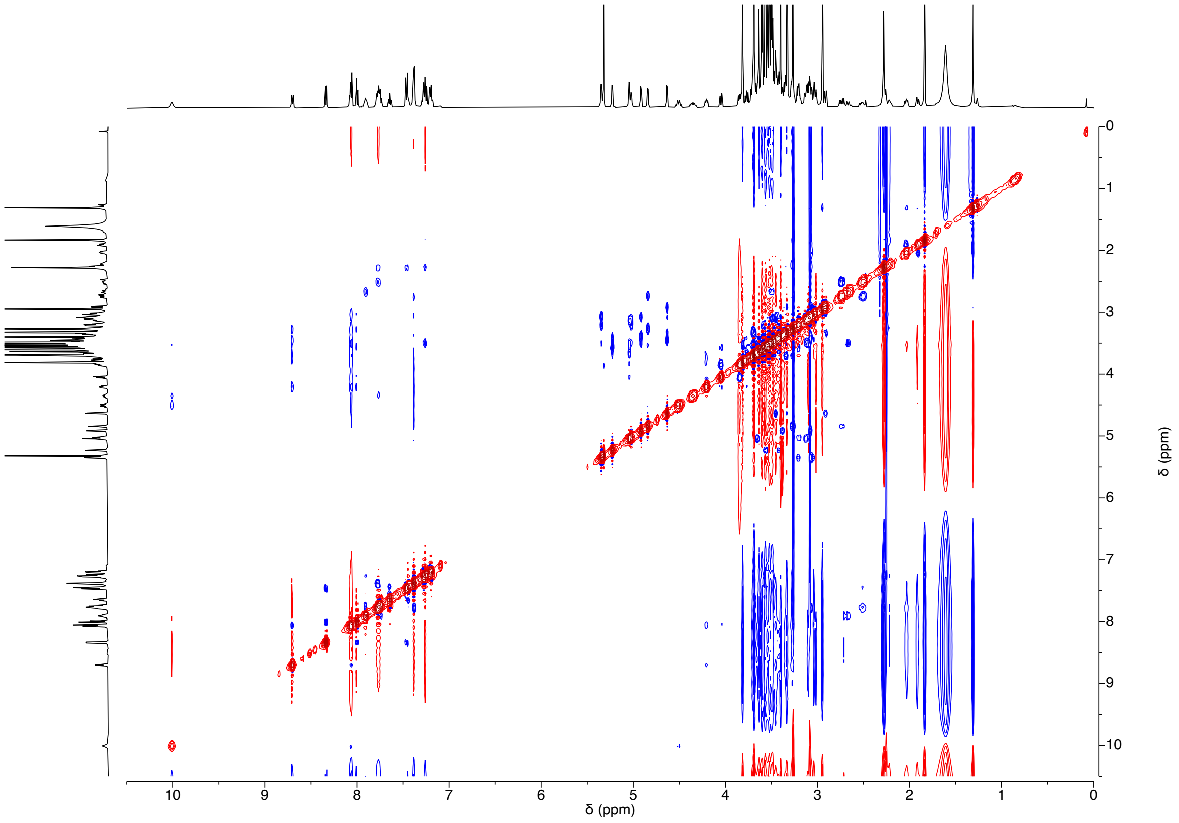

Figure S7f. $^{1}H/^{1}H$ ROESY NMR (500 MHz, CD_2Cl_2) spectrum of compounds **7a,b**.

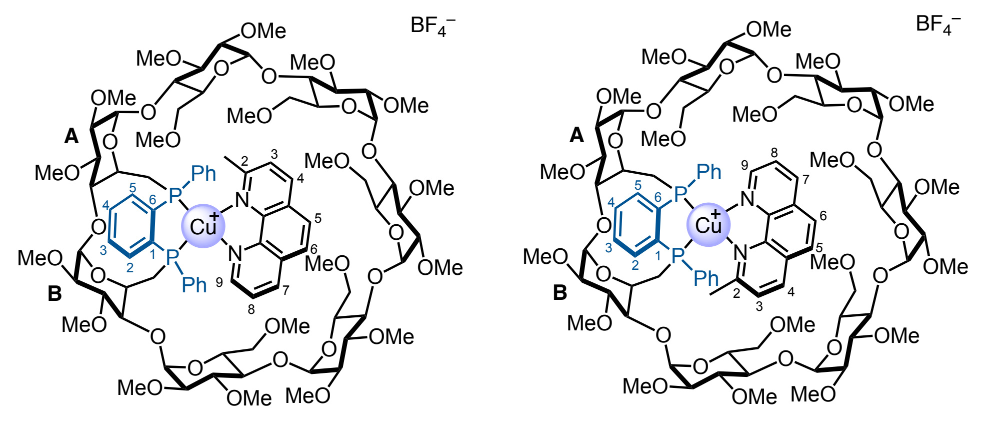

7a (98%) **7b** (2%)

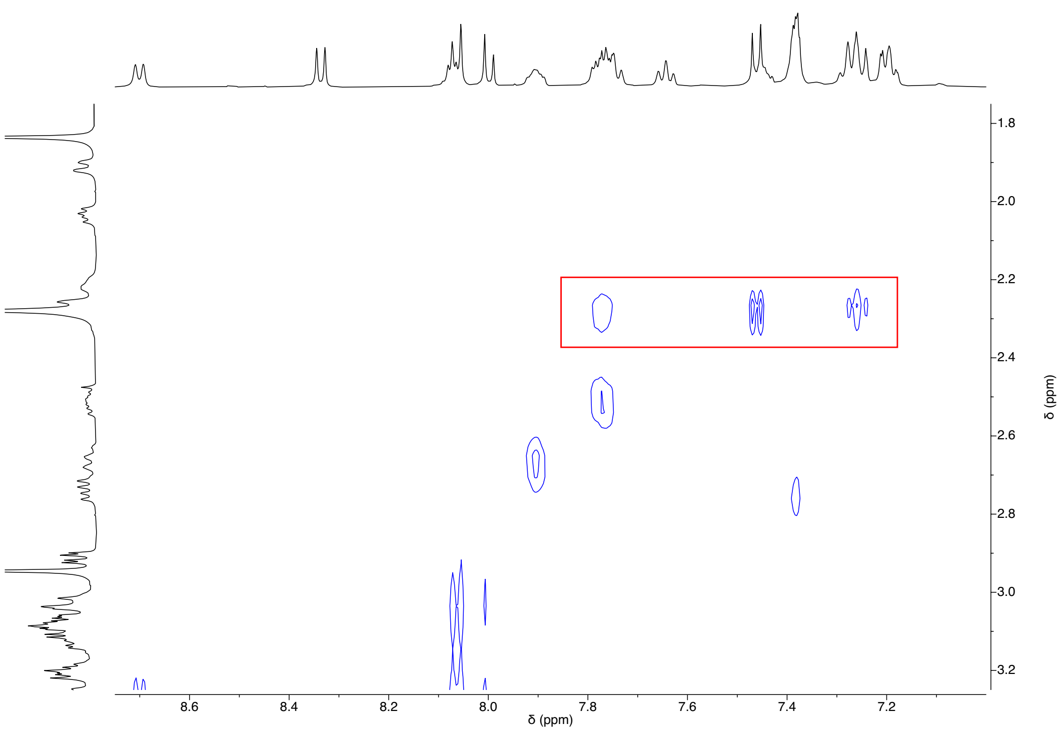

Figure S7g. Part of the ^{1}H/^{1}H ROESY NMR (500 MHz, CD_2Cl_2) spectrum of compounds **7a,b** showing correlations between aromatic diphosphane protons and the mmp 2-methyl protons of **7a** (red rectangle).

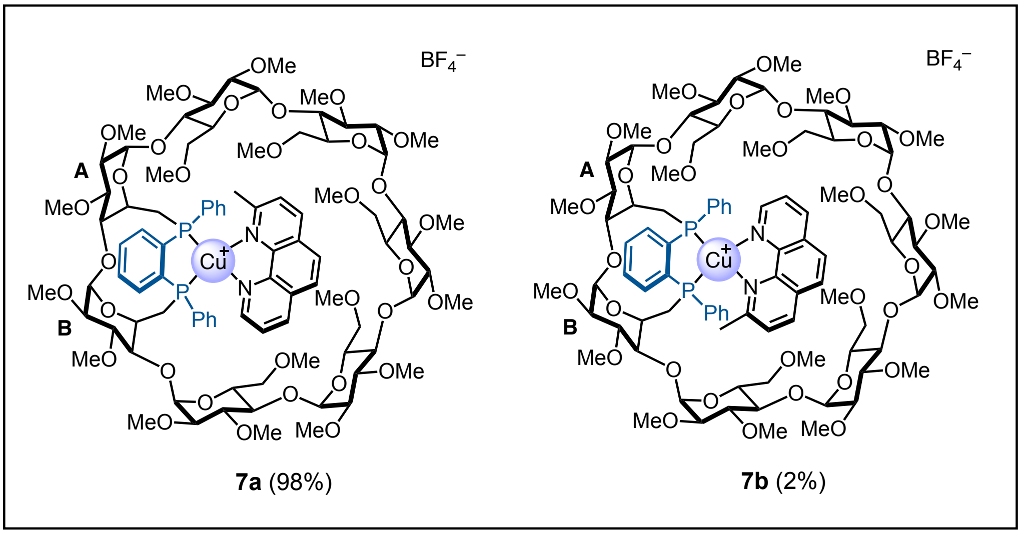

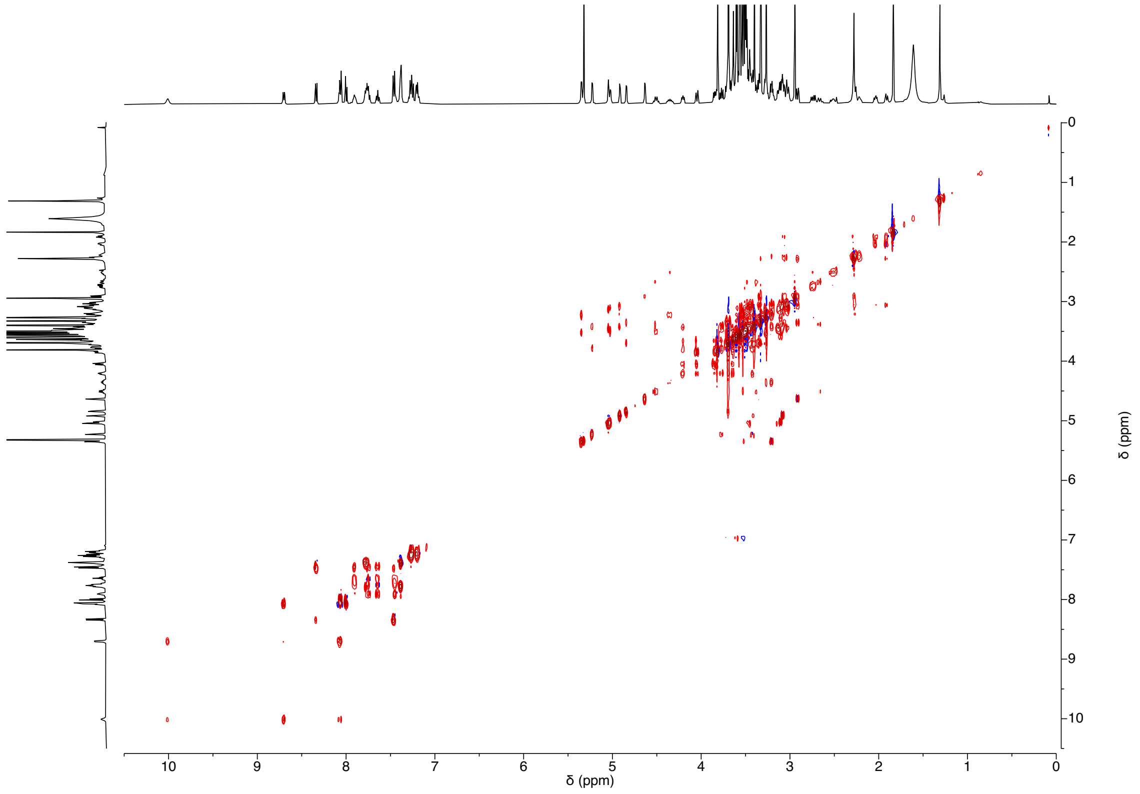

Figure S7h. ^{1}H/^{1}H TOCSY NMR (500 MHz, CD_2Cl_2) spectrum of compounds **7a,b**.

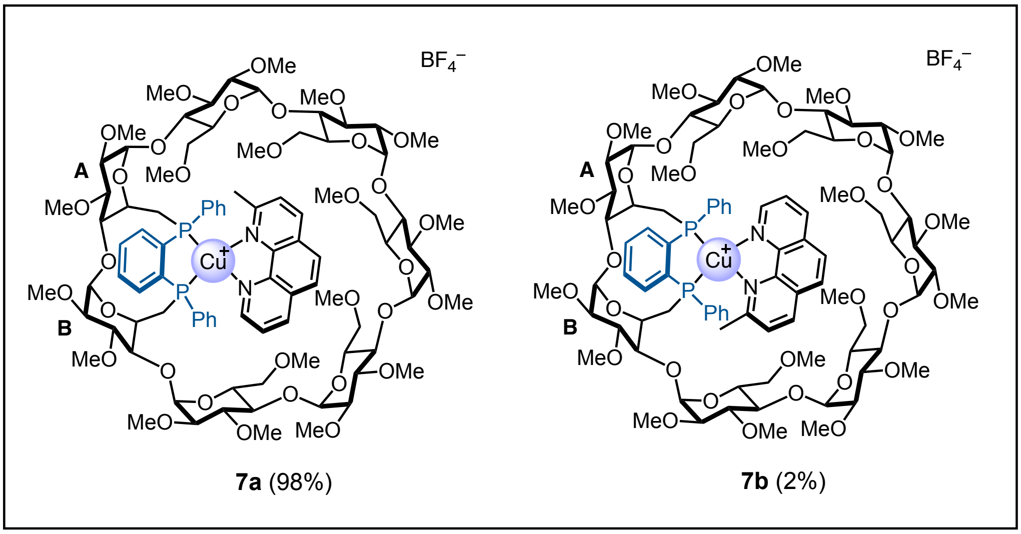

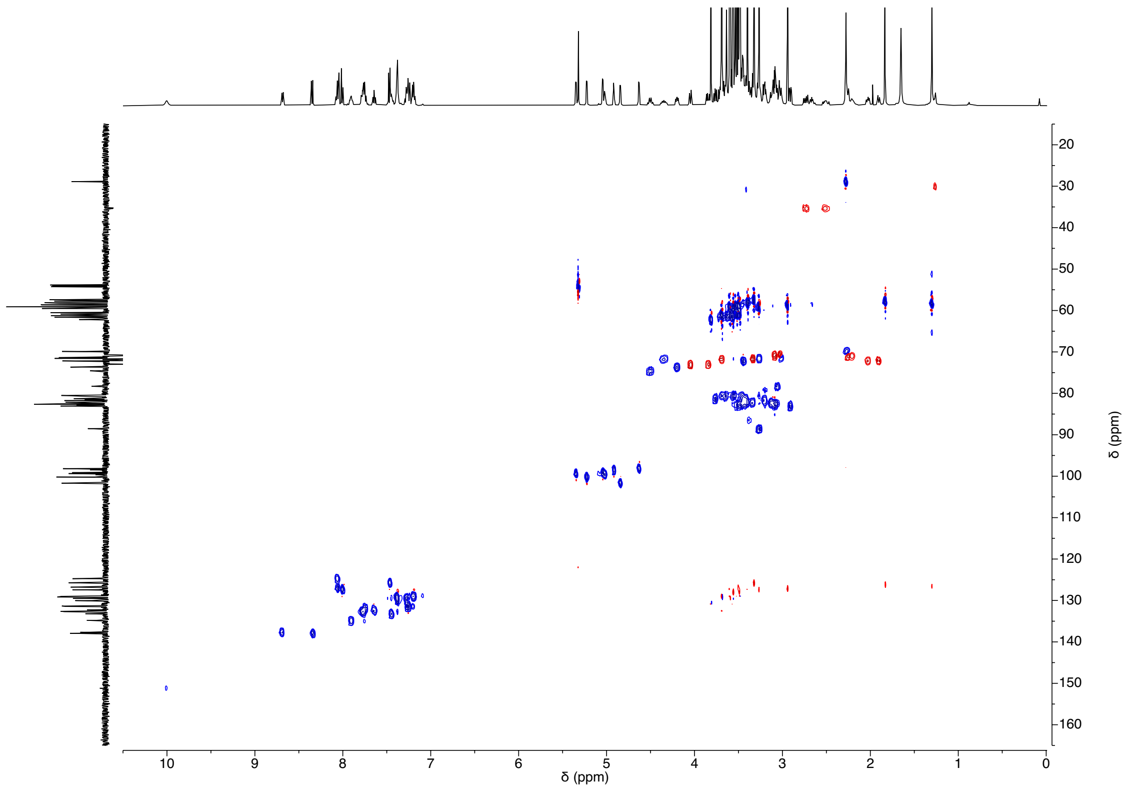

Figure S7i. ¹H/¹³C{¹H} HSQC NMR (500 MHz, CD_2Cl_2) spectrum of compounds **7a,b**.

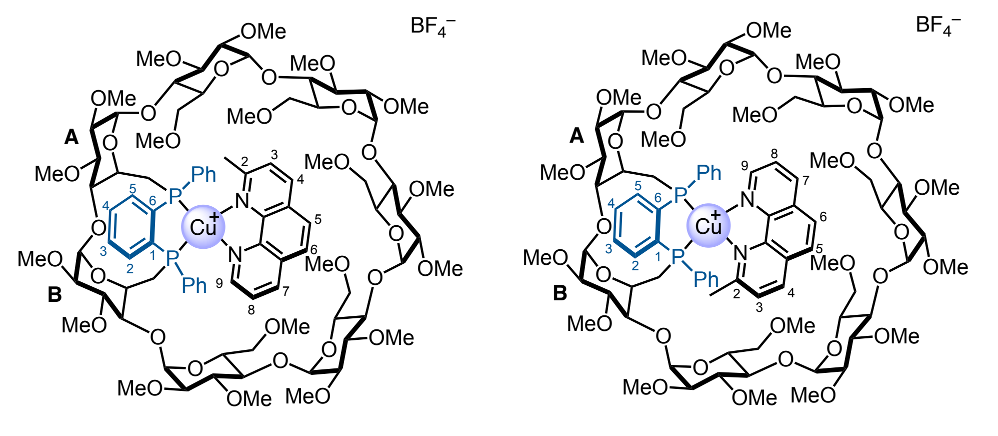

7a (98%) **7b** (2%)

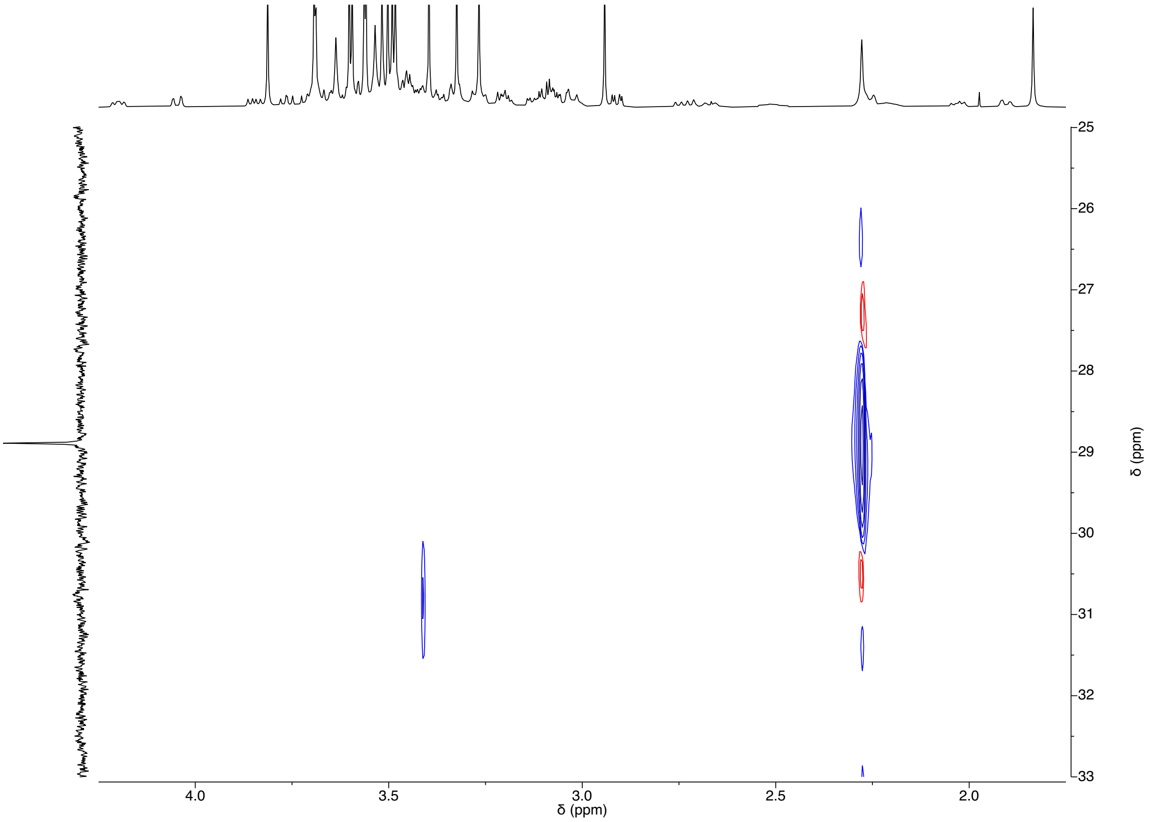

Figure S7j. Part of $^{1}H/^{13}C\{^{1}H\}$ HSQC NMR (500 MHz, CD_2Cl_2) spectrum of compounds **7a,b** showing correlations between the mmp 2-methyl protons of the two isomers (**7a** and **7b**) and their corresponding carbons.

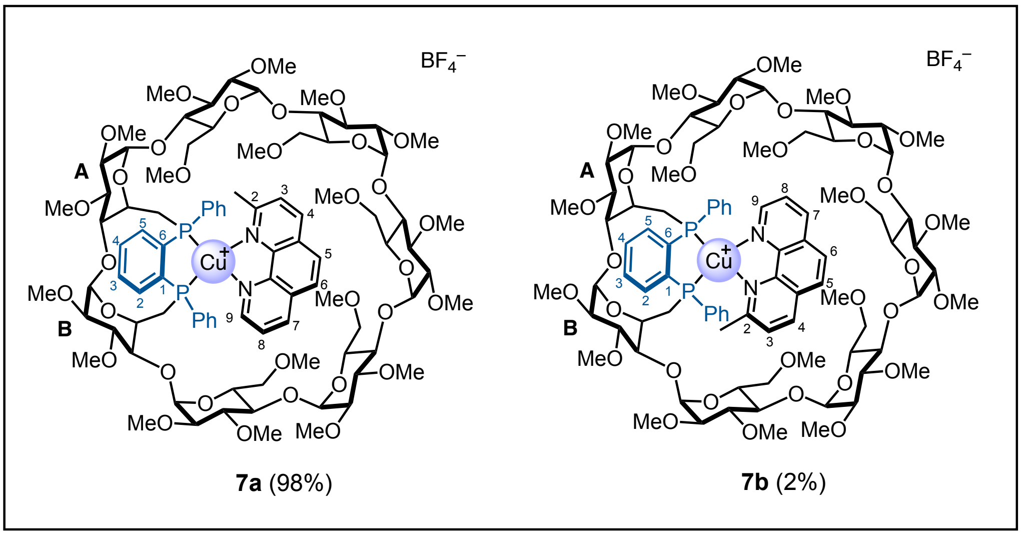

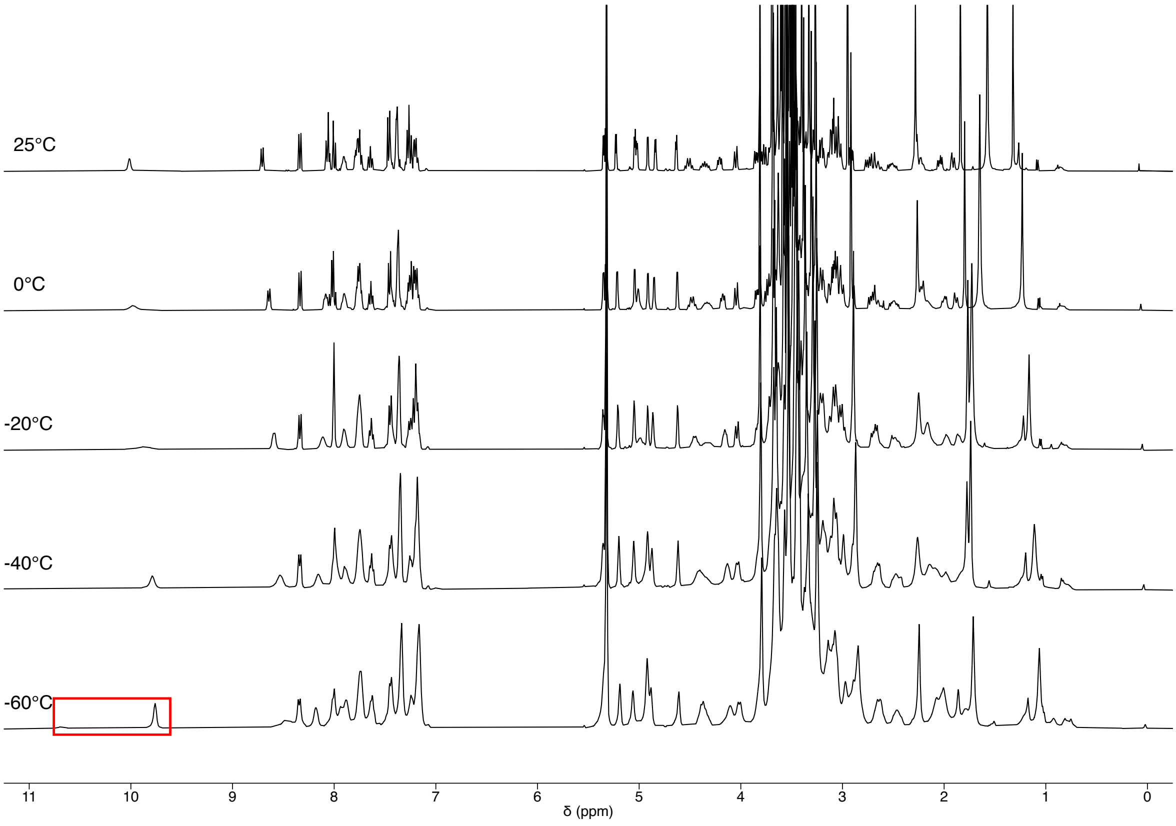

Figure S7k. ^{1}H NMR (400 MHz, CD_2Cl_2) spectra of compounds **7a,b** recorded at low temperatures showing the mmp H-9 signals corresponding to the two conformers of **7a** (red rectangle).

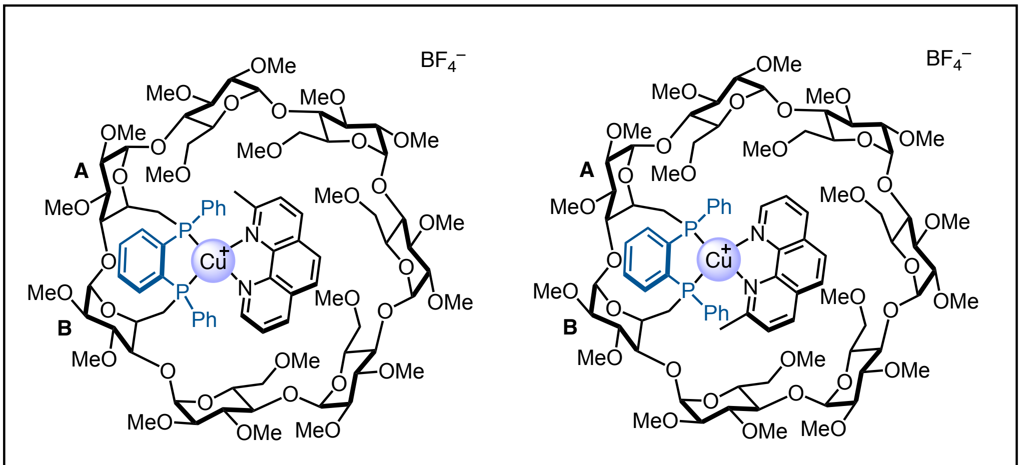

7a (98%) **7b** (2%)

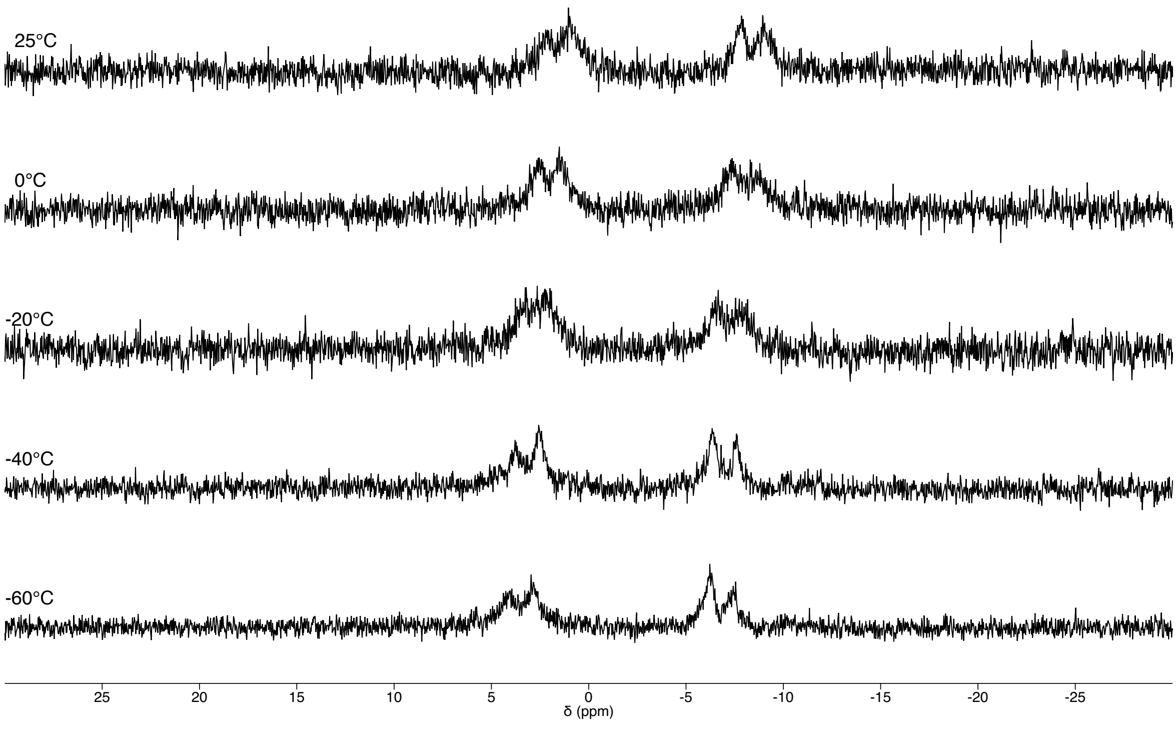

Figure S7I. $^{31}P\{^1H\}$ NMR (162 MHz, CD_2Cl_2) spectra of compounds **7a,b** recorded at low temperatures.

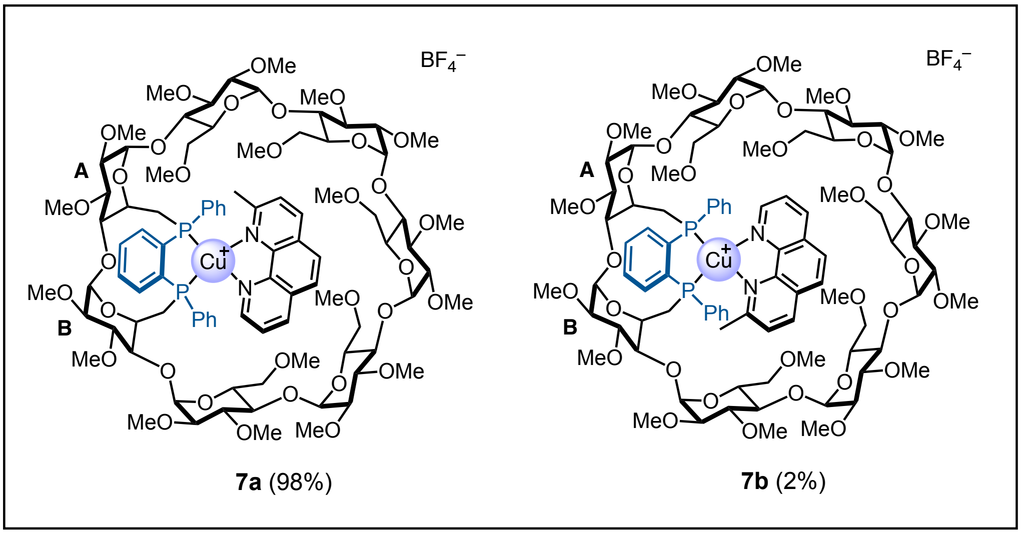

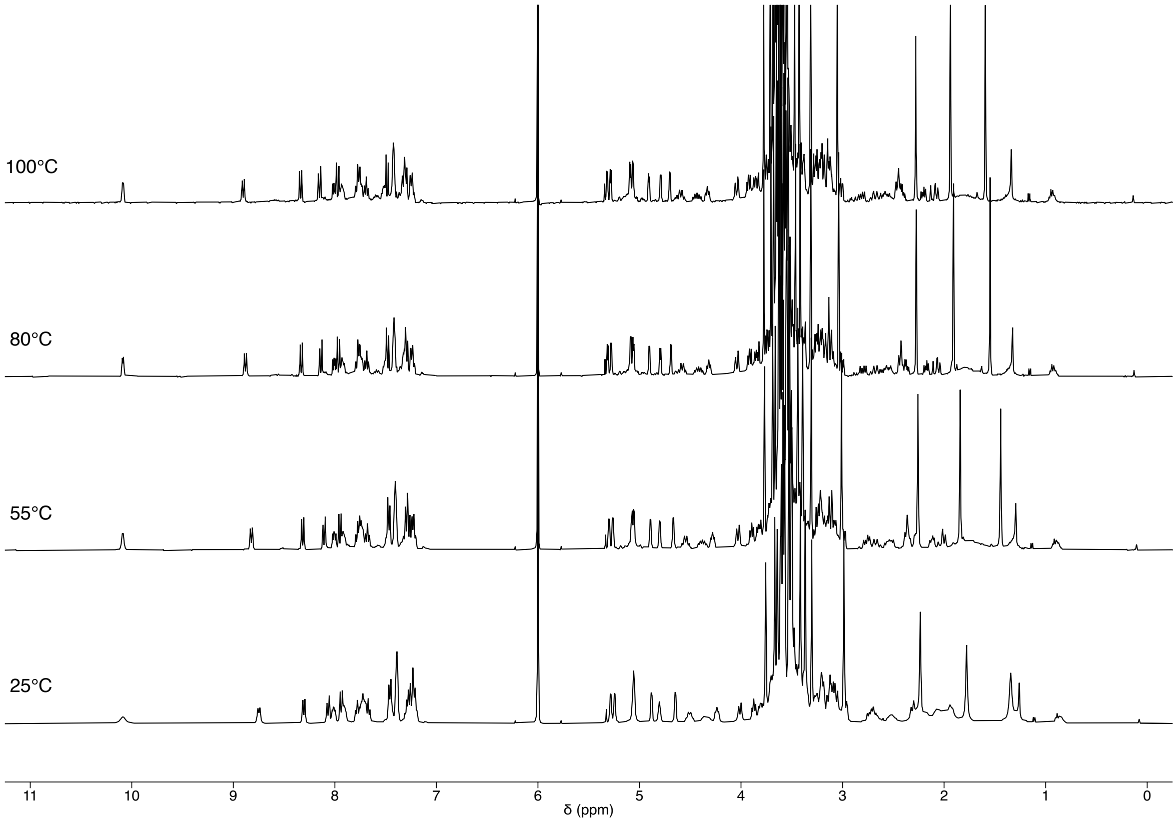

Figure S7m. ^{1}H NMR (400 MHz, $C_2D_2Cl_4$) spectra of compounds **7a,b** recorded at high temperatures.

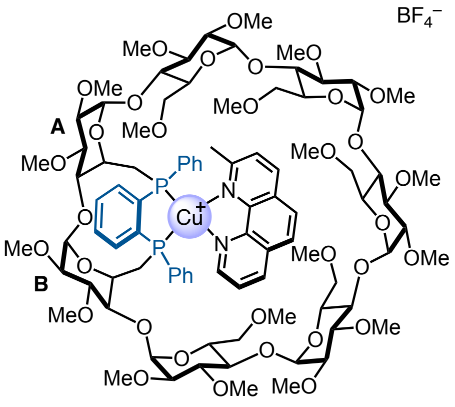

7a (98%)

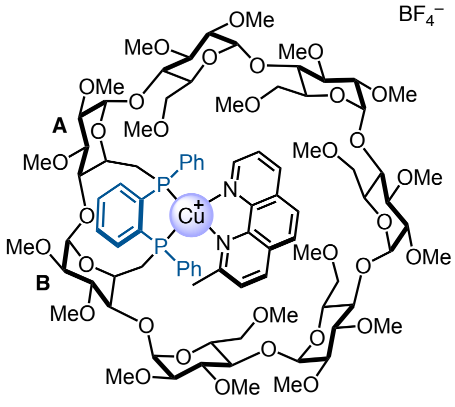

7b (2%)

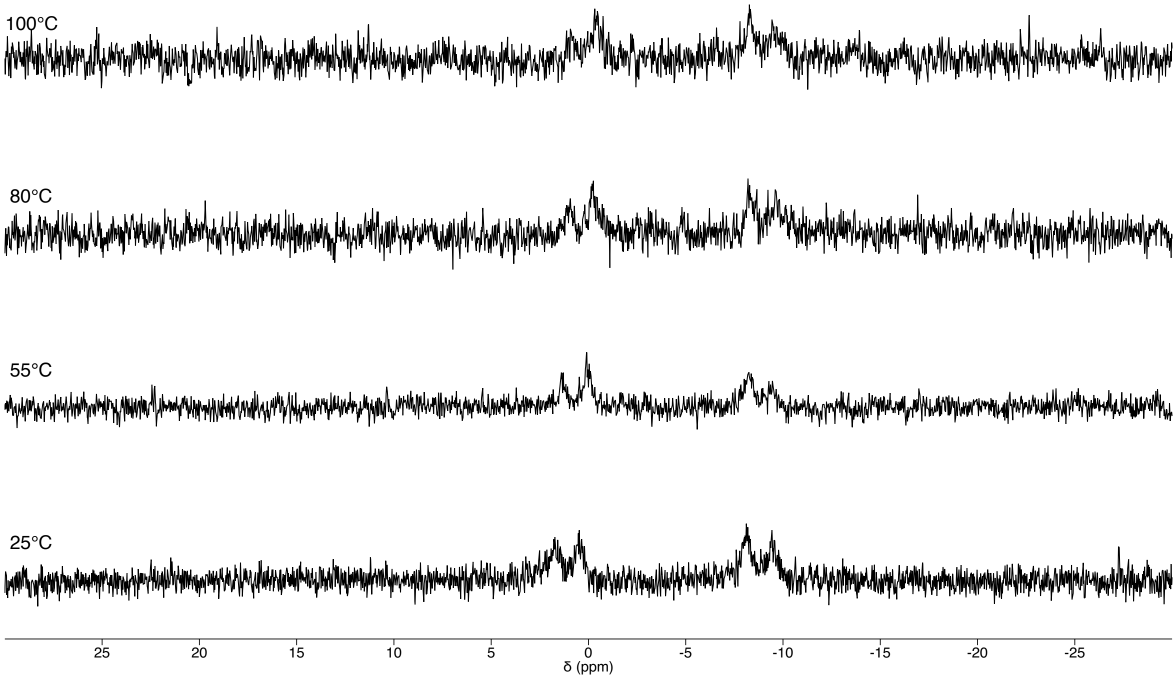

Figure S7n. $^{31}P\{^{1}H\}$ NMR (162 MHz, $C_2D_2Cl_4$) spectra of compounds **7a,b** recorded at high temperatures.

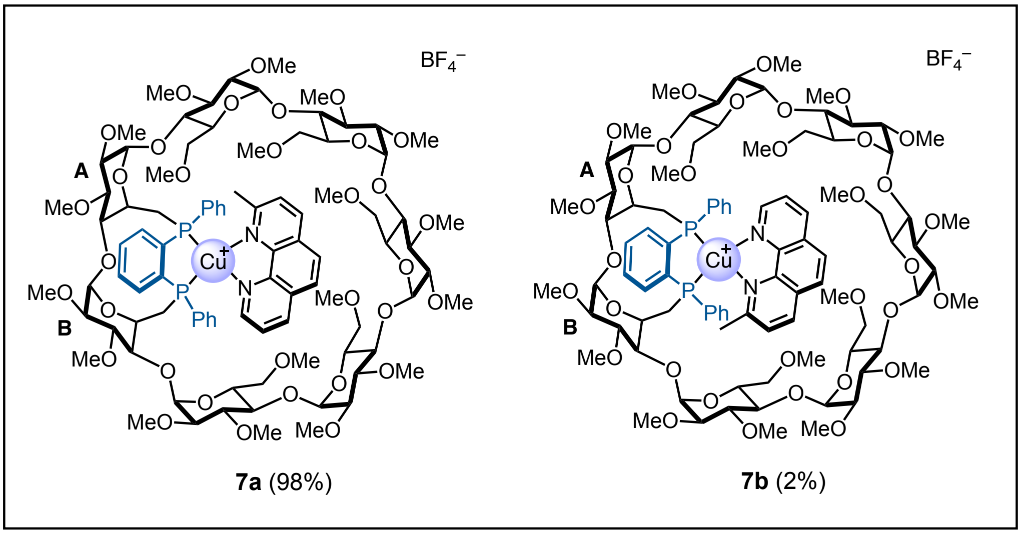

Acquisition Parameter

Source Type	ESI	Capillary	4500 V	Nebulizer	0.3 Bar	Corona	169 nA
Ion Polarity	Positive	Set Capillary Exit	150.0 V	Dry Gas	4.0 l/min	Set Hexapole RF	300.0 V
n/a	n/a	Set Skimmer 1	50.0 V	Dry Heater	200 °C	APCI Heater	514 °C

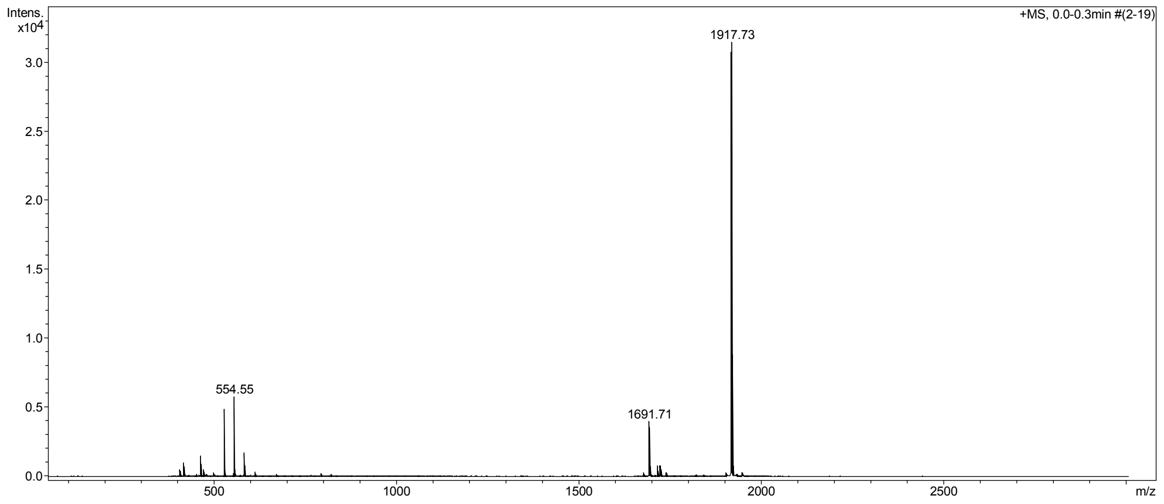

Figure S7o. ESI-TOF mass spectrum of compounds **7a,b**.

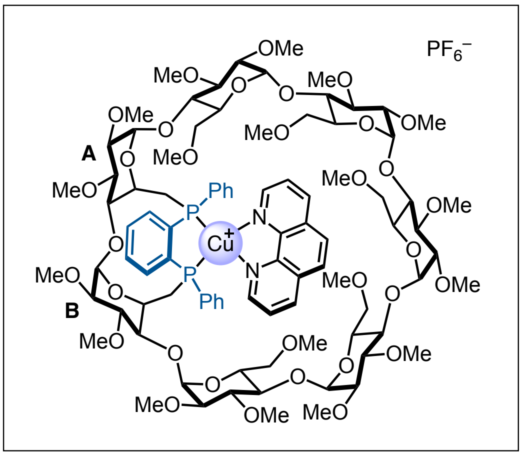

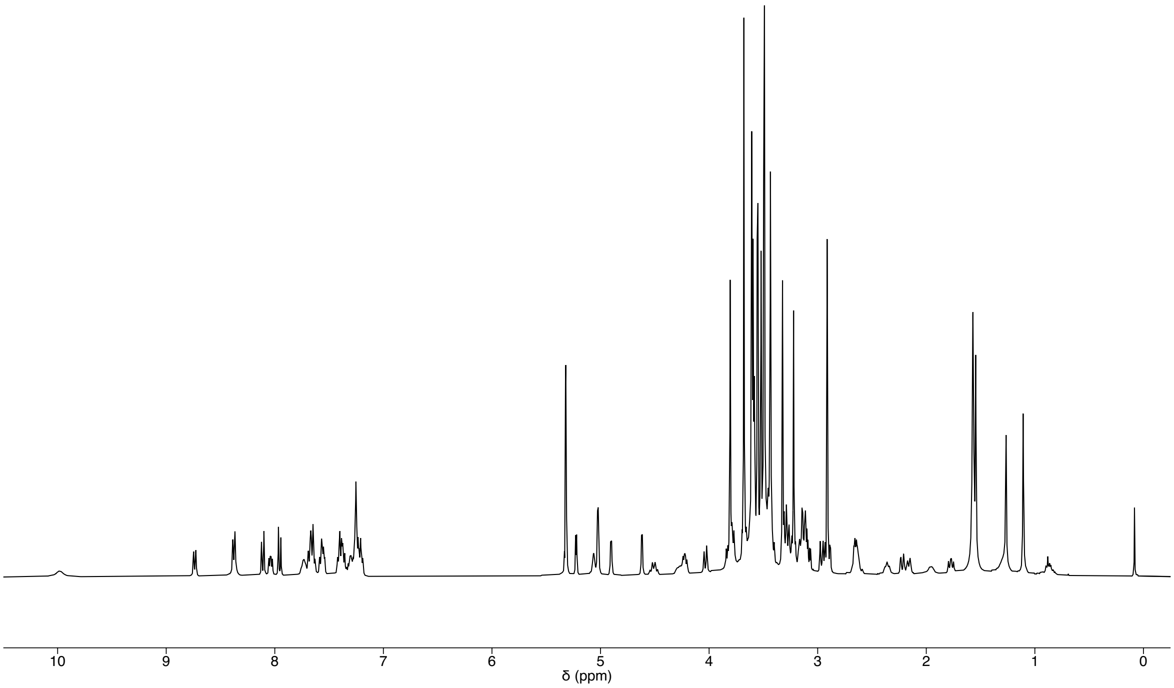

Figure S8a. ^{1}H NMR (400 MHz, CD_2Cl_2) spectrum of compound **8**.

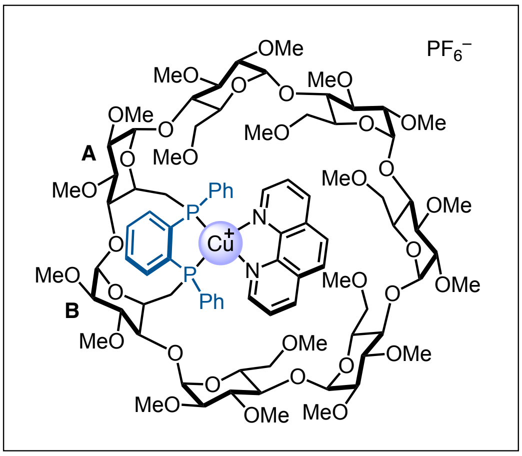

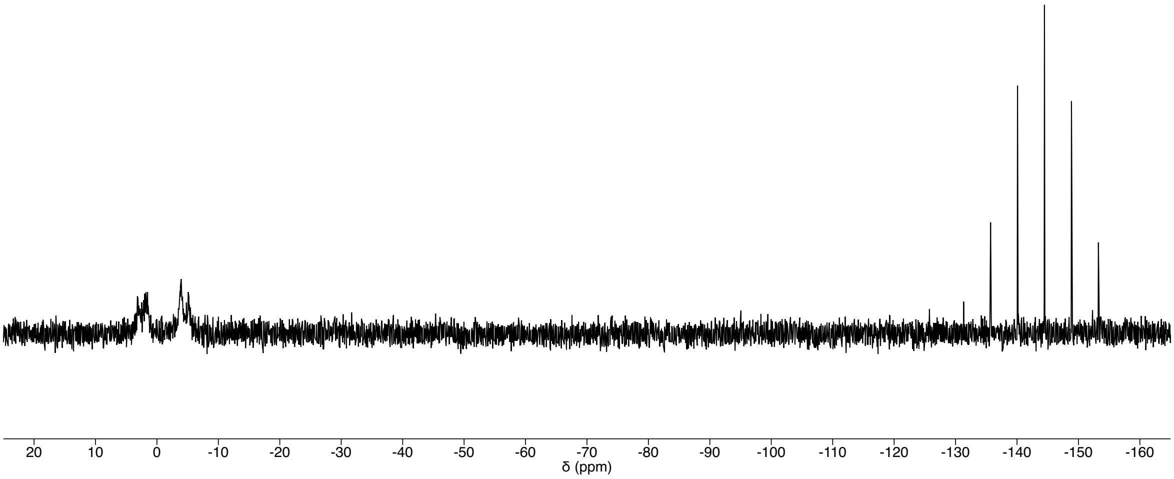

Figure S8b. $^{31}P\{^{1}H\}$ NMR (162 MHz, CD_2Cl_2) spectrum of compound **8**.

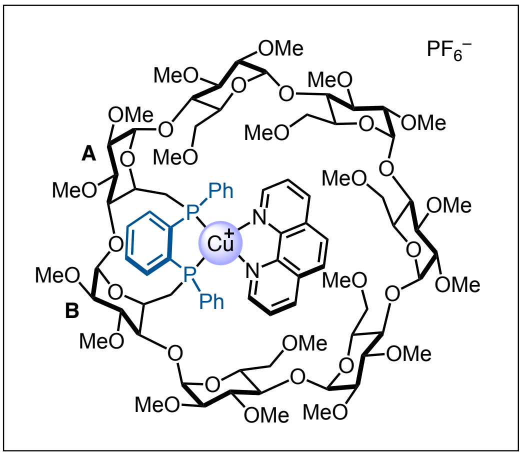

Acquisition Parameter

Source Type	ESI	Capillary	4500 V	Nebulizer	0.3 Bar	Corona	196 nA
Ion Polarity	Positive	Set Capillary Exit	150.0 V	Dry Gas	4.0 l/min	Set Hexapole RF	300.0 V
n/a	n/a	Set Skimmer 1	50.0 V	Dry Heater	200 °C	APCI Heater	514 °C

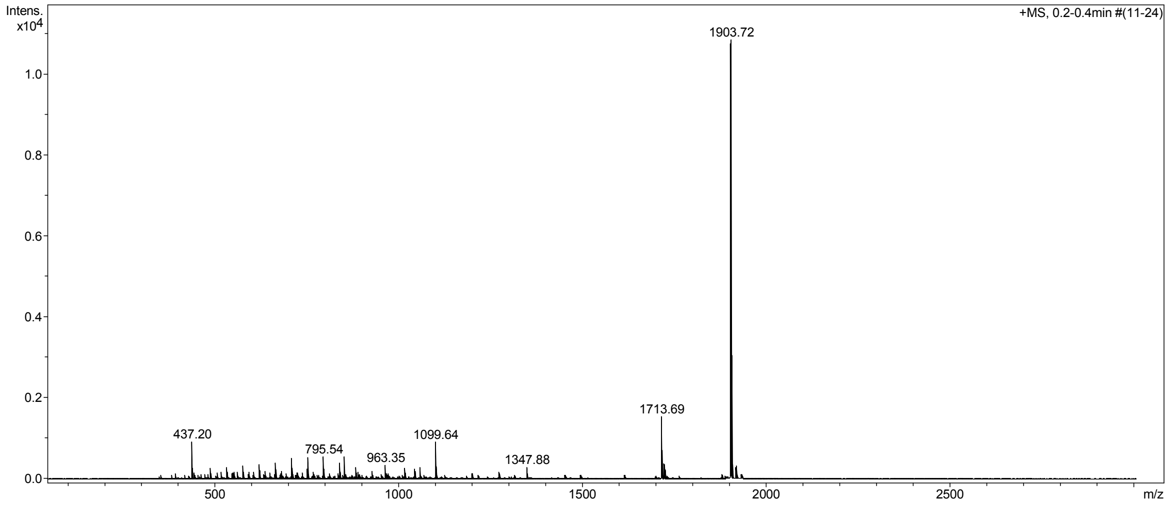

Figure S8c. ESI-TOF mass spectrum of compound **8**.

Electrochemical measurements

The electrochemical properties of compounds **4c**,**5**,**6**,**7a,b** were determined by cyclic voltammetry (CV) and Osteryoung square wave voltammetry (OSWV) in CH_2Cl_2. The solutions used during the electrochemical studies were typically 0.9 × 10^{-3} M in compound and 0.1 M in supporting electrolyte. The supporting electrolyte [$n$$Bu_4N$]$BF_4$ (Fluka, 99% electrochemical grade) was used as received and simply degassed under argon. DCM was dried using an MB SPS-800 solvent purification system just prior to use. The measurements were carried out with an Autolab PGSTAT100 potentiostat controlled by GPES 4.09 software. Experiments were performed at room temperature in a homemade, airtight three-electrode cell connected to a vacuum/argon line. The reference electrode consisted of a saturated calomel electrode (SCE) separated from the solution by a bridge compartment. The counter electrode was a platinum wire of about 1 cm^2 apparent surface. The working electrode was a Pt microdisk (0.5 mm diameter). Before each measurement, the solutions were degassed by bubbling Ar, and the working electrode was polished with a polishing machine (Presi P230). Under these experimental conditions, F_c^+/F_c is observed at +0.55 ± 0.01 V *vs* SCE. OSWVs were obtained using an amplitude of 20 mV, a frequency of 20 Hz, and a step potential of 5 mV.

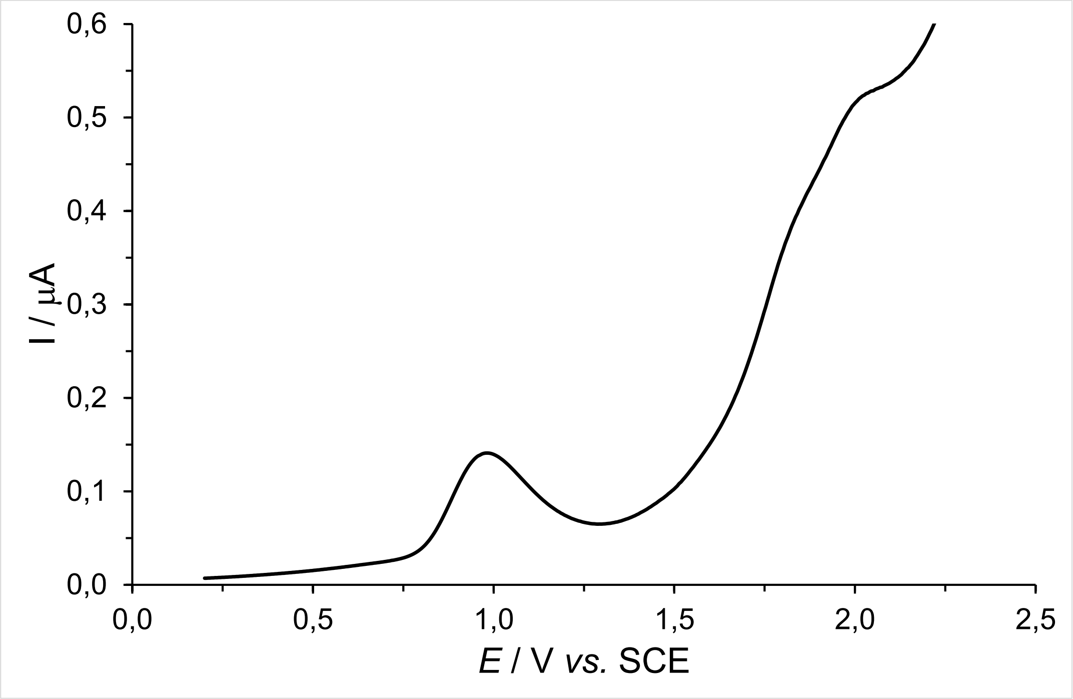

Figure S9a. OSWVs: anodic scan of ligand **4c** on a Pt electrode in CH_2Cl_2 + 0.1 M $[nBu_4N]BF_4$ at room temperature (frequency 20 Hz, amplitude 20 mV, step potential 5 mV.

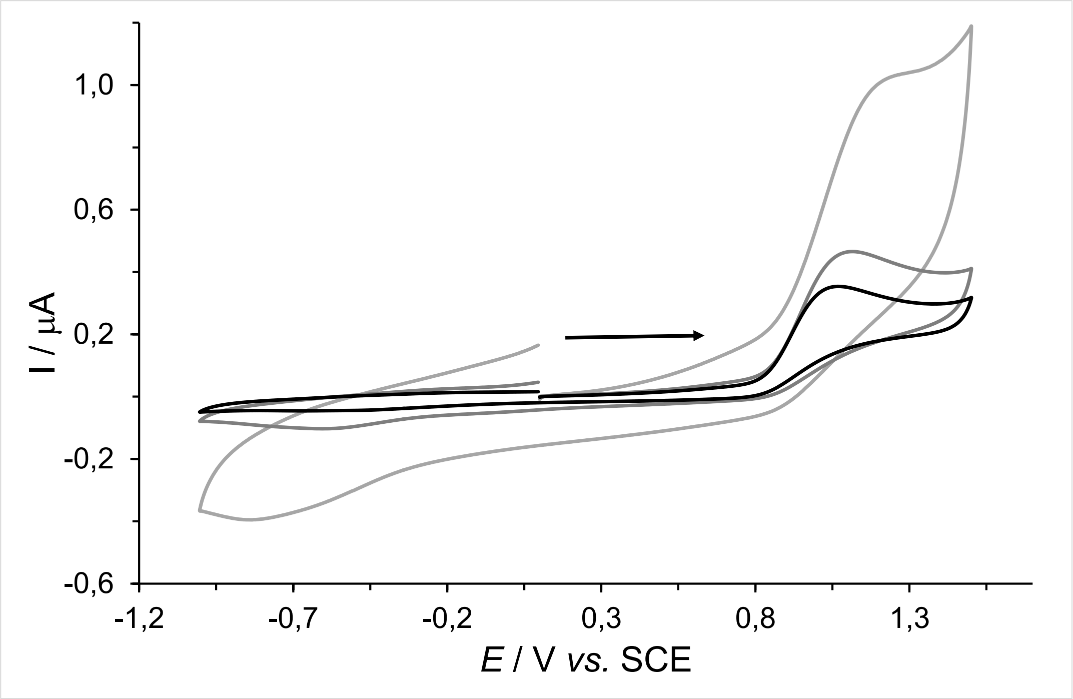

Figure S9b. Segmented cyclic voltammograms of ligand **4c** at 0.1 (black), 0.2 (gray), and 1.0 (light gray) V/s.

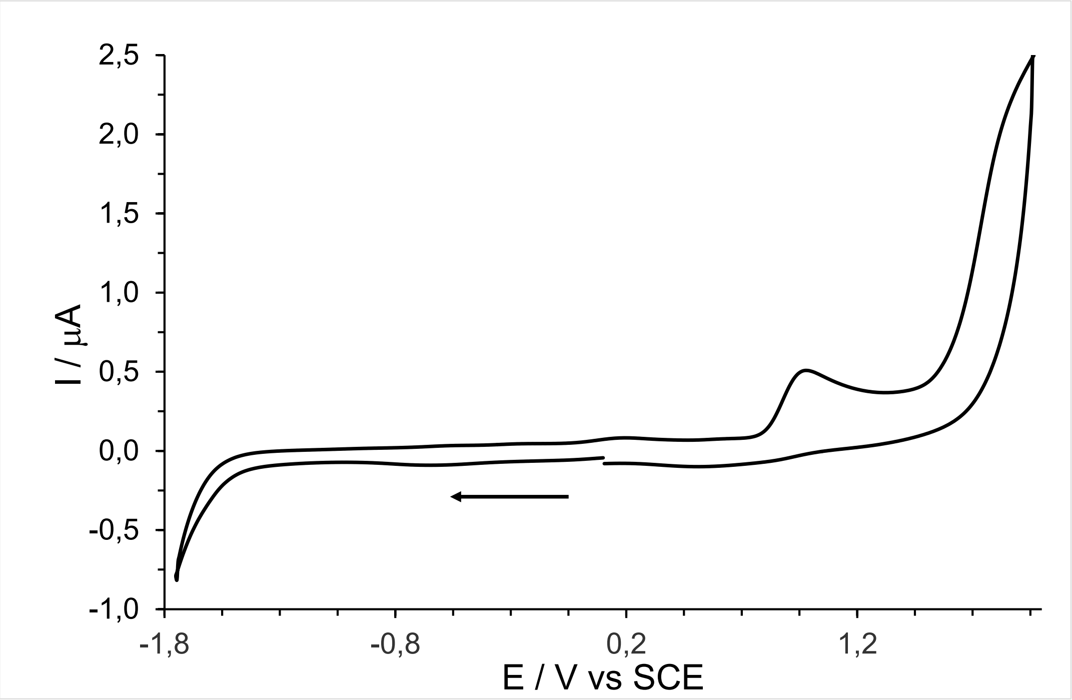

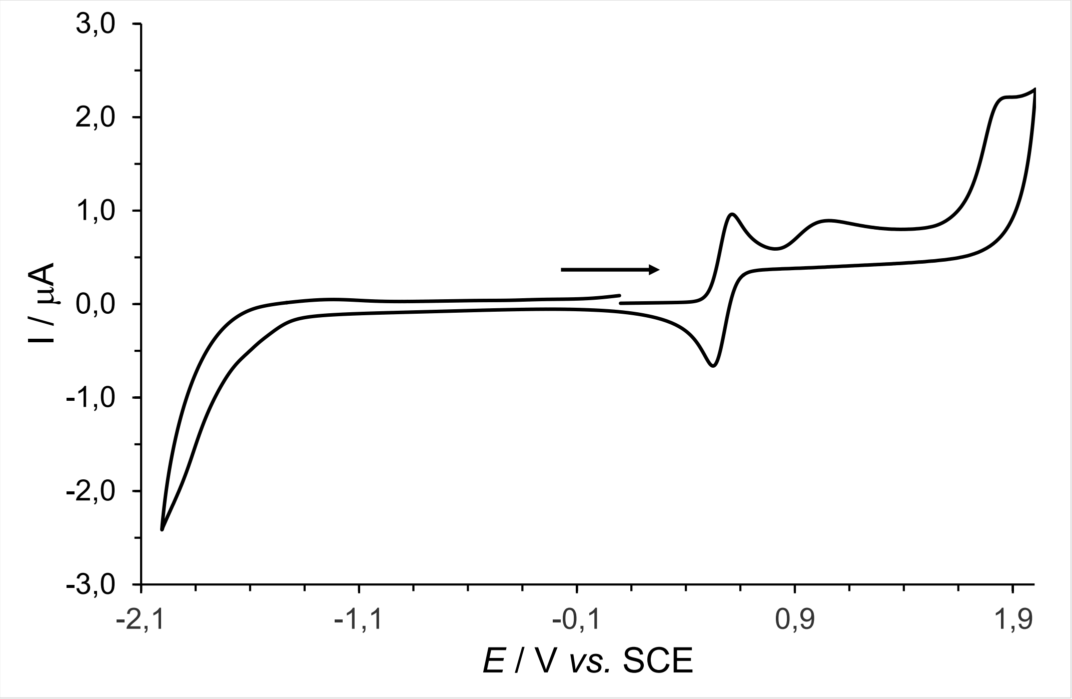

Figure S9c. Cyclic voltammograms of ligand **4c** alone (top), and in the presence of Fc at 0.2 V/s (bottom).

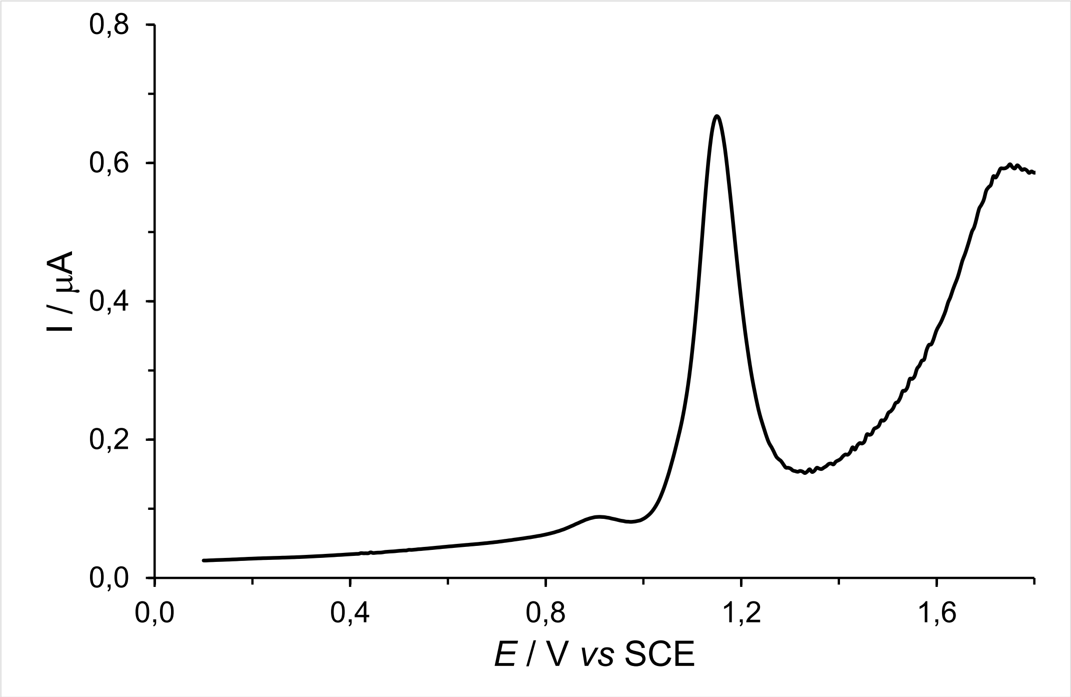

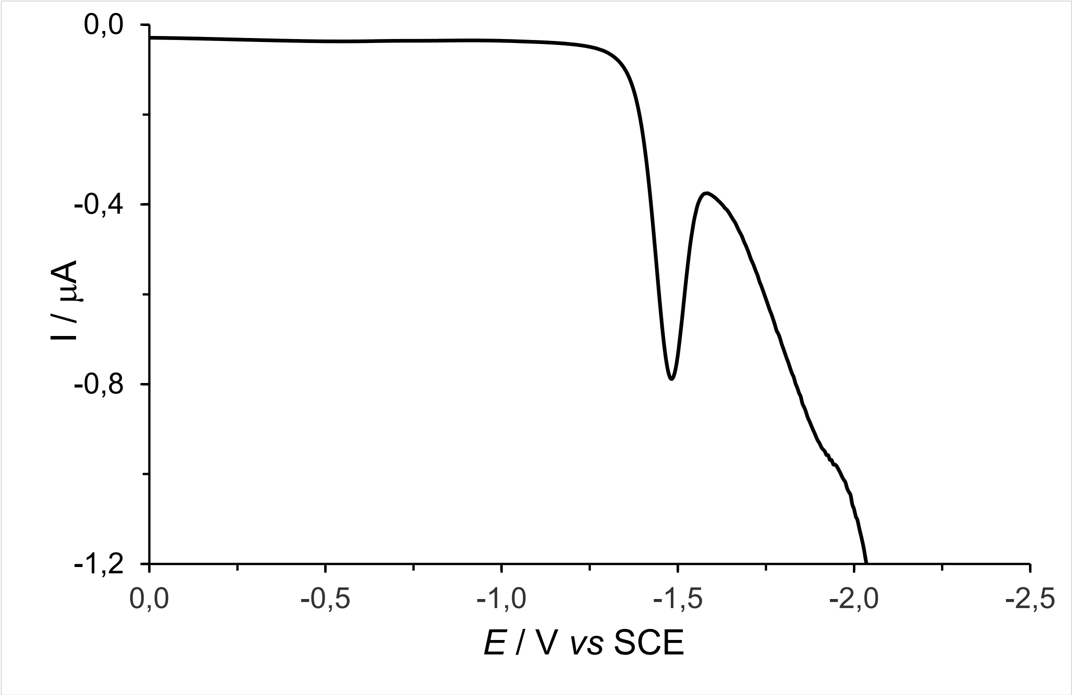

Figure S10a. OSWVs: anodic (top) and cathodic (bottom) scans of complex **5** on a Pt electrode in CH_2Cl_2 + 0.1 M $[nBu_4N]BF_4$ at room temperature (frequency 20 Hz, amplitude 20 mV, step potential 5 mV).

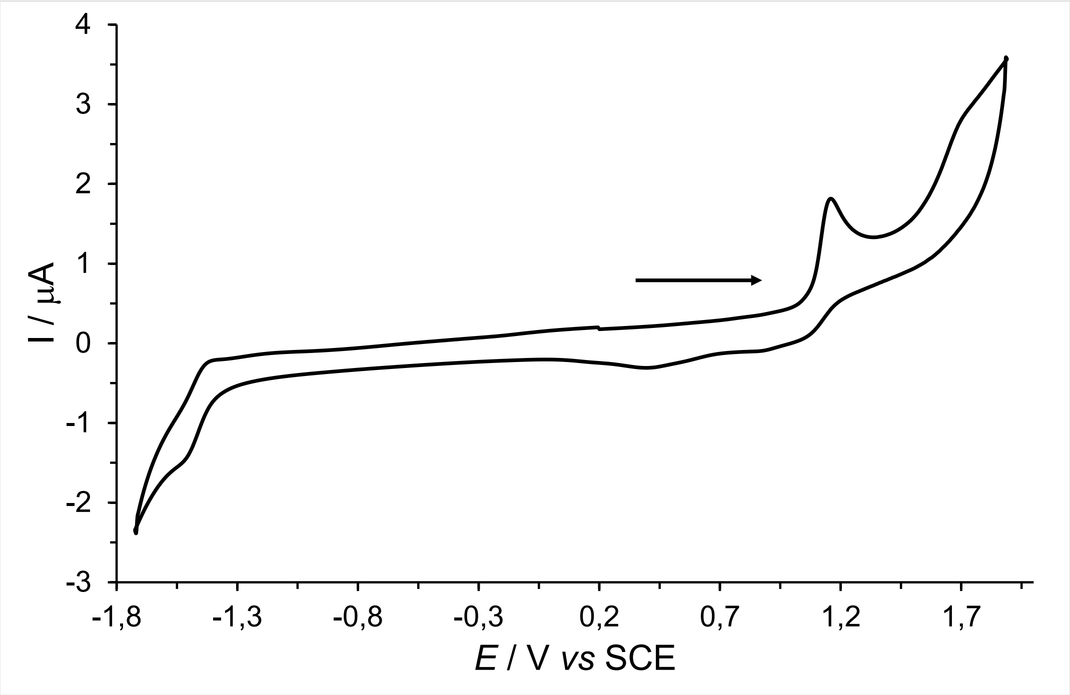

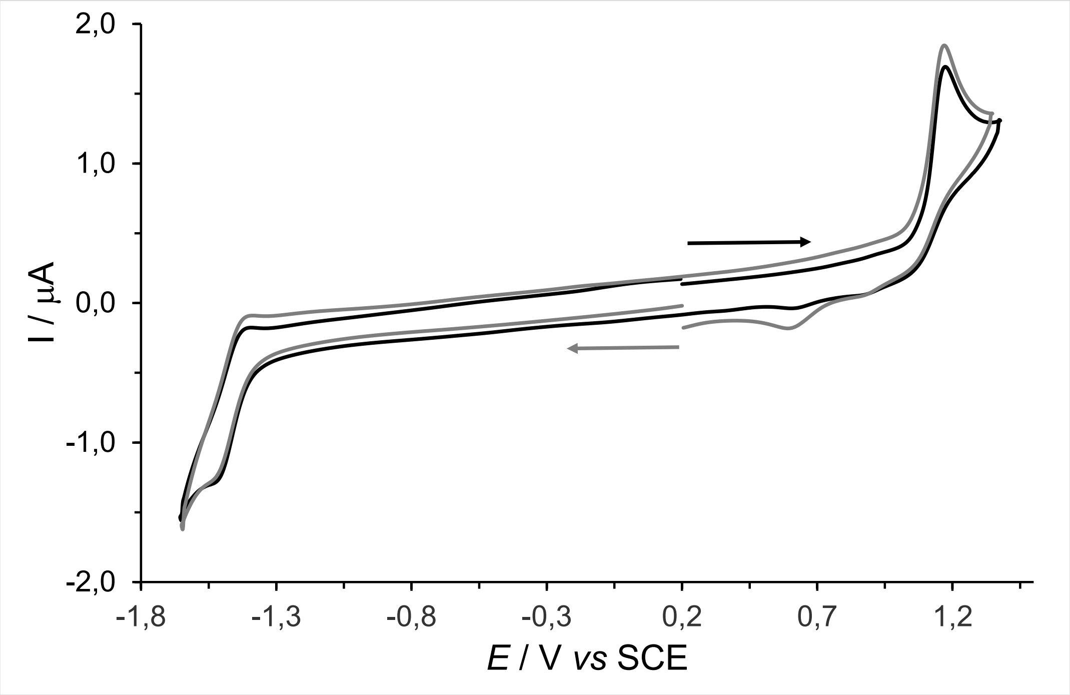

Figure S10b. Cyclic voltammogram of complex **5** at 0.2 V/s (top), and segmented cyclic voltammograms of its first oxidation and reduction processes at 0.2 V/s (bottom).

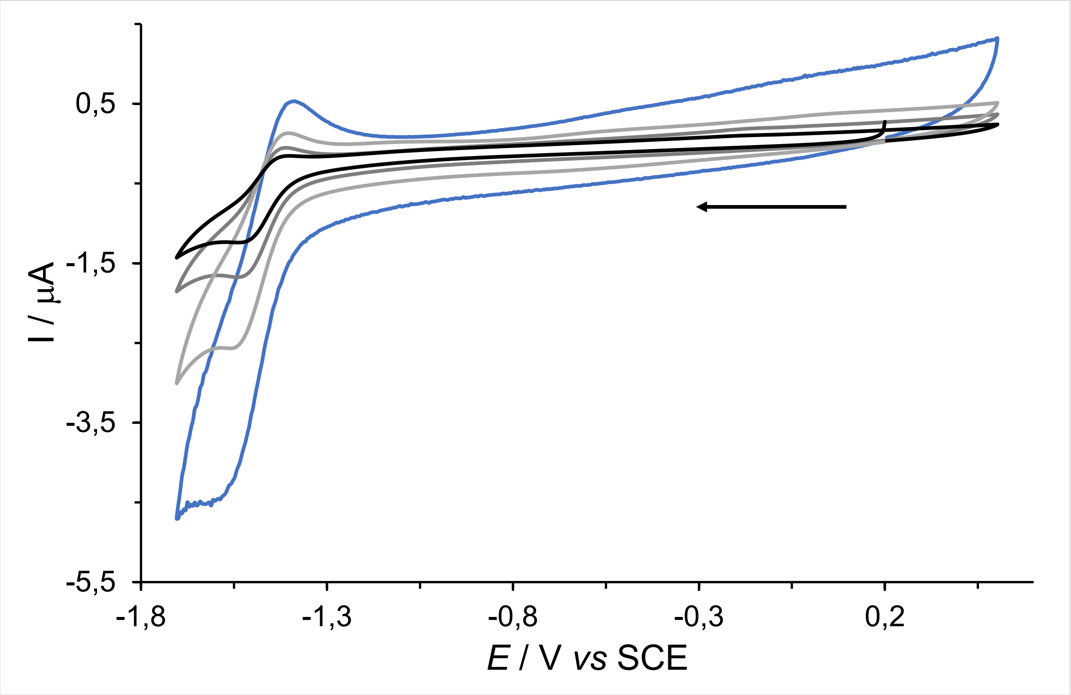

Figure S10c. Cyclic voltammograms of the first reduction process of complex **5** at respectively, 0.2, 0.5, 1, and 5 V/s respectively, from black line to blue line.

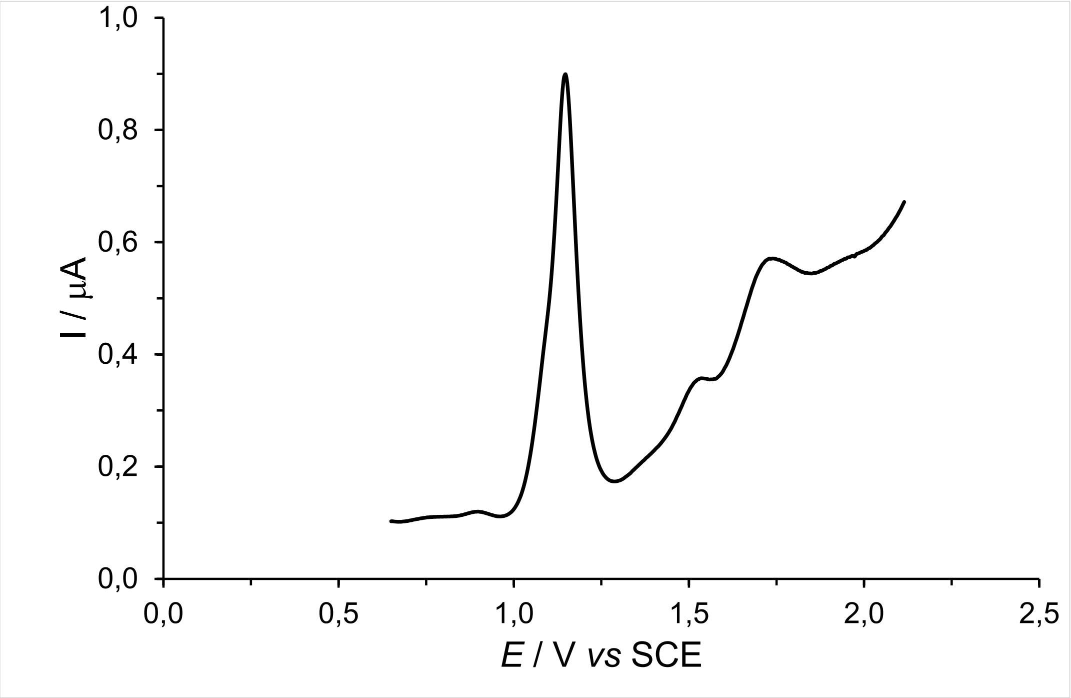

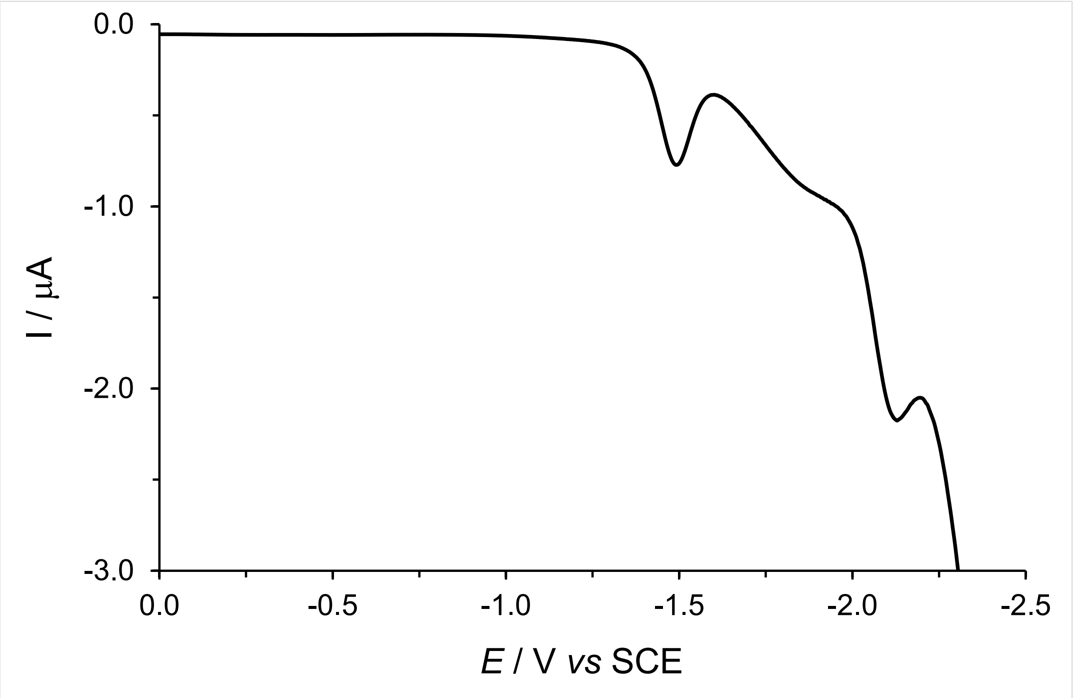

Figure S11a. OSWVs: anodic (top) and cathodic (bottom) scans of complex **6** on a Pt electrode in CH_2Cl_2 + 0.1 M $[nBu_4N]BF_4$ at room temperature (frequency 20 Hz, amplitude 20 mV, step potential 5 mV).

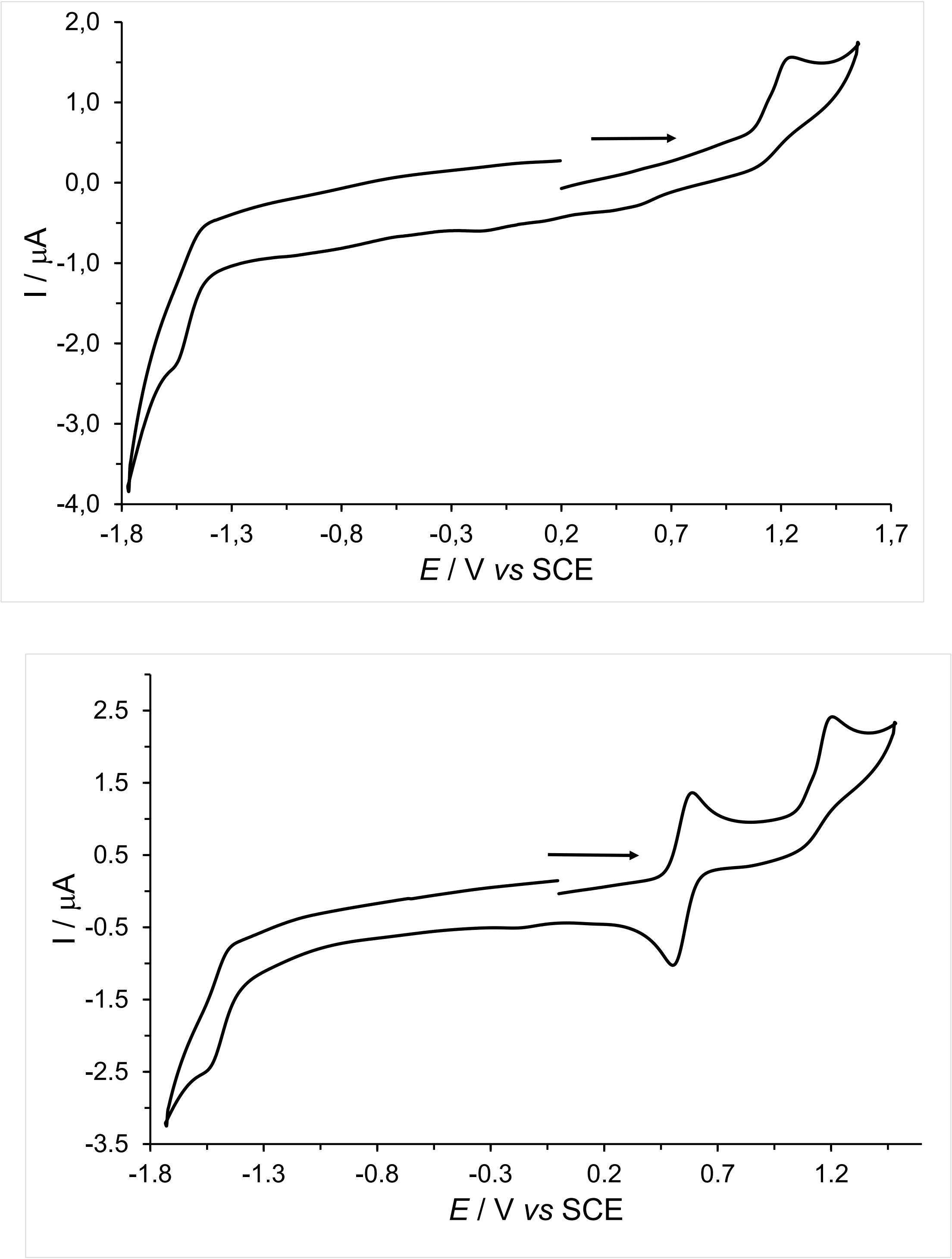

Figure S11b. Cyclic voltammograms of complex **6** alone (top), and in the presence of Fc at 0.2 V/s (bottom).

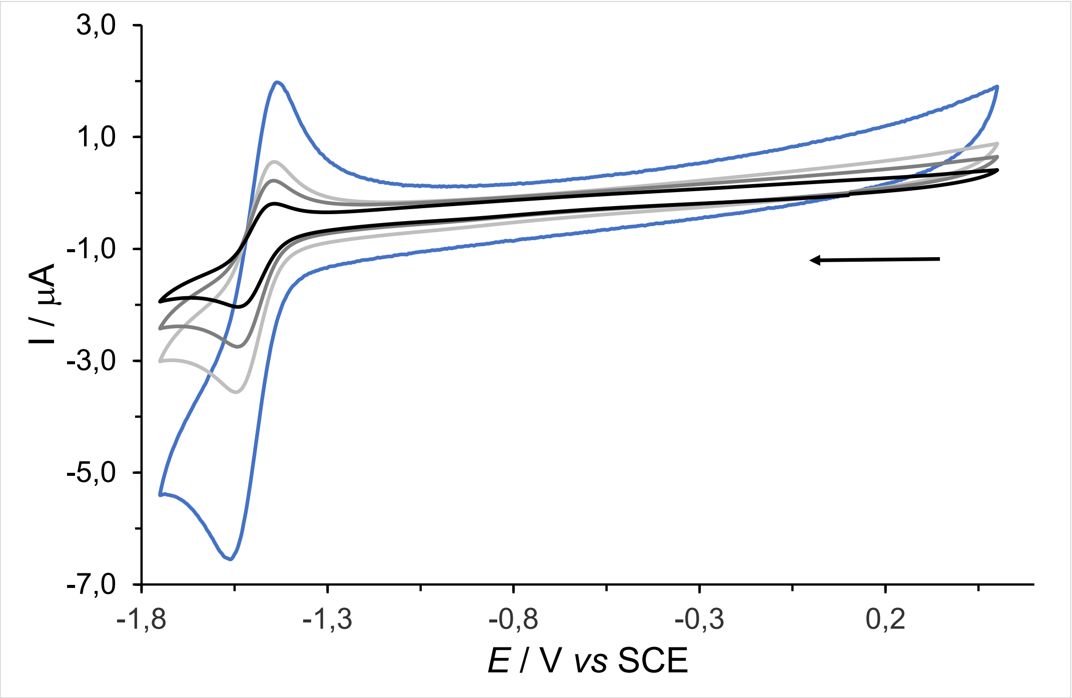

Figure S11c. Cyclic voltammograms of the first reduction process of complex **6** at respectively, 0.2, 1, 5 and 10 V/s respectively, from black line to blue line.

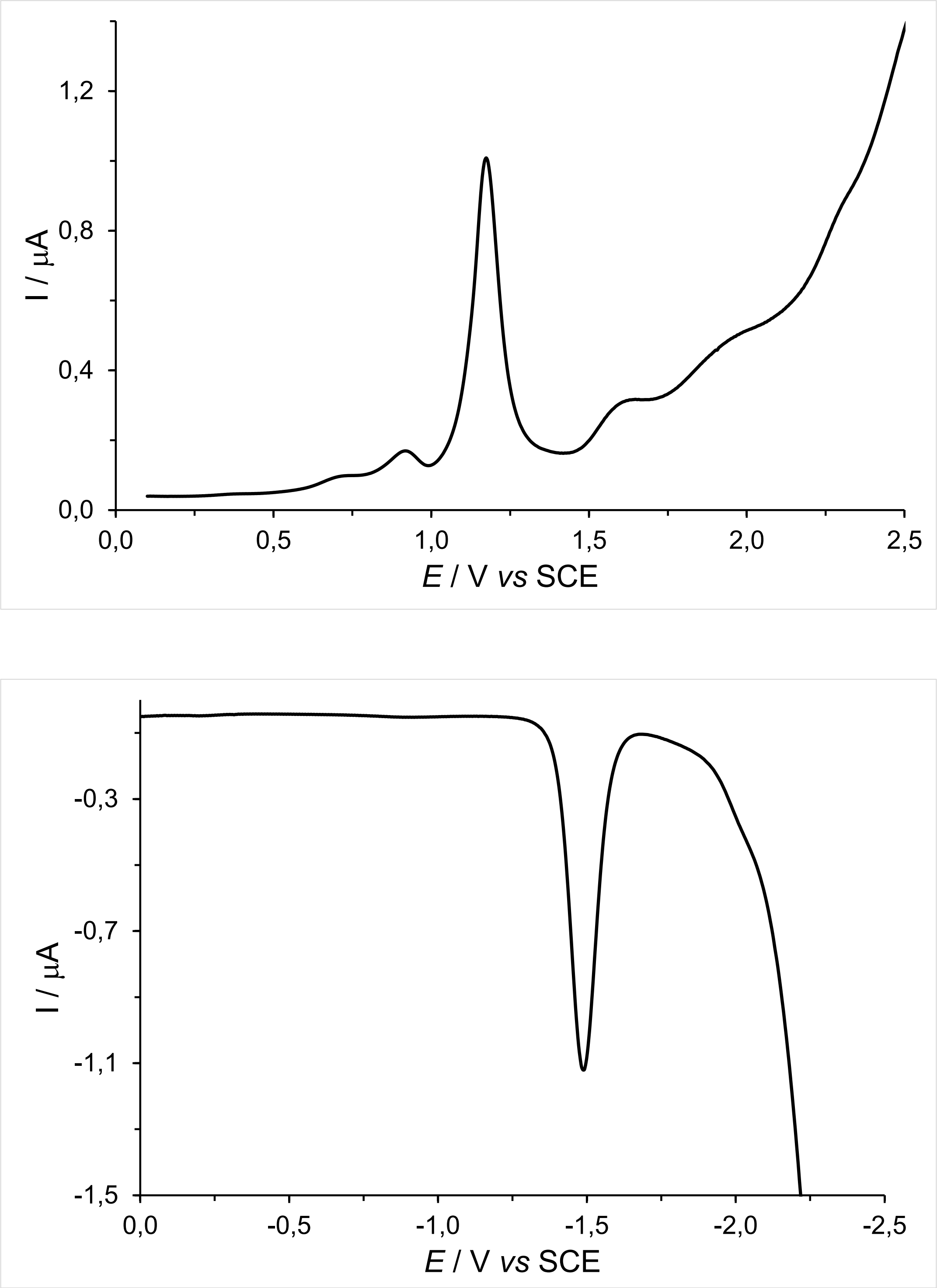

Figure S12a. OSWVs: anodic (top) and cathodic (bottom) scans of complexes **7a,b** on a Pt electrode in CH_2Cl_2 + 0.1 M $[nBu_4N]BF_4$ at room temperature (frequency 20 Hz, amplitude 20 mV, step potential 5 mV).

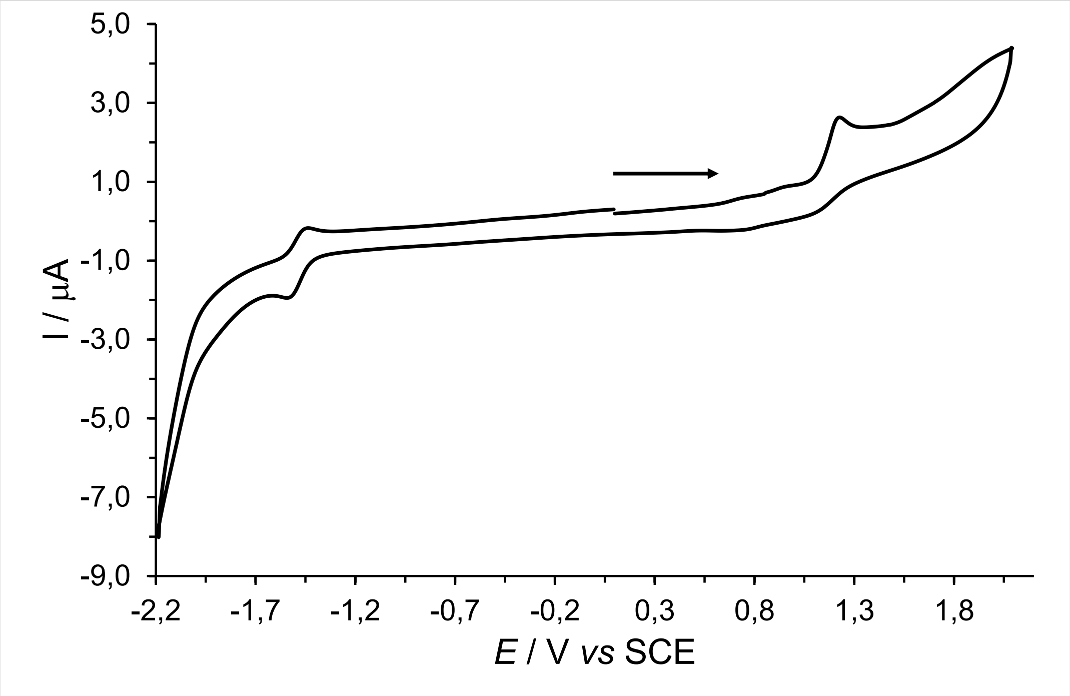

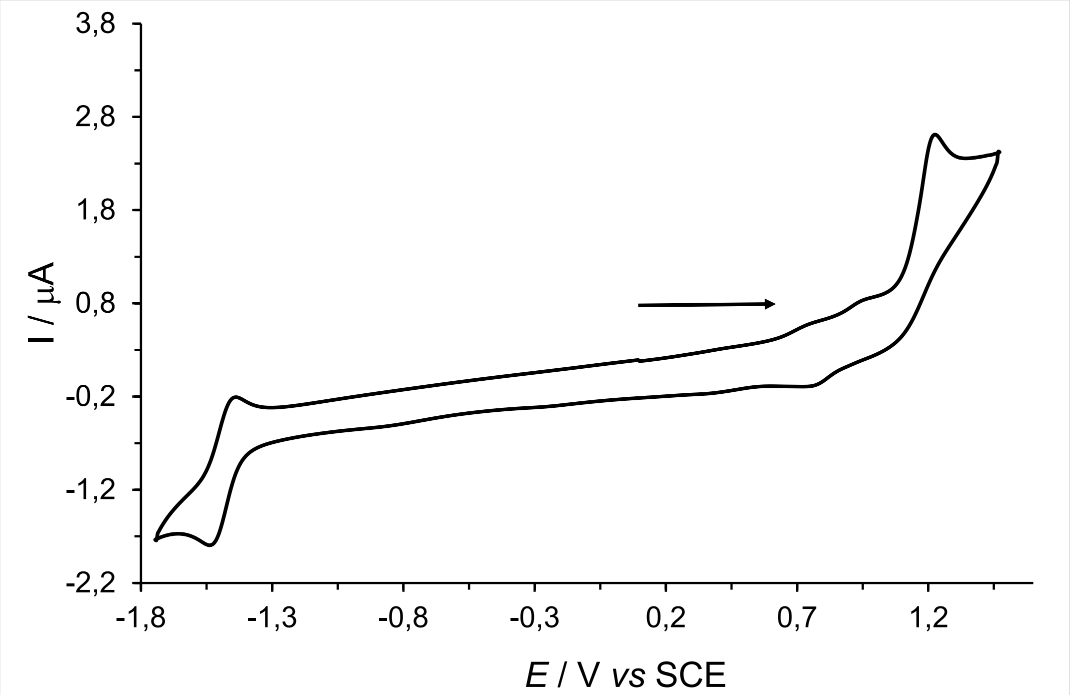

Figure S12b. Cyclic voltammogram of complexes **7a,b** at 0.2 V/s (top), and segmented cyclic voltammogram of its first oxidation and reduction processes at 0.2 V/s (bottom).

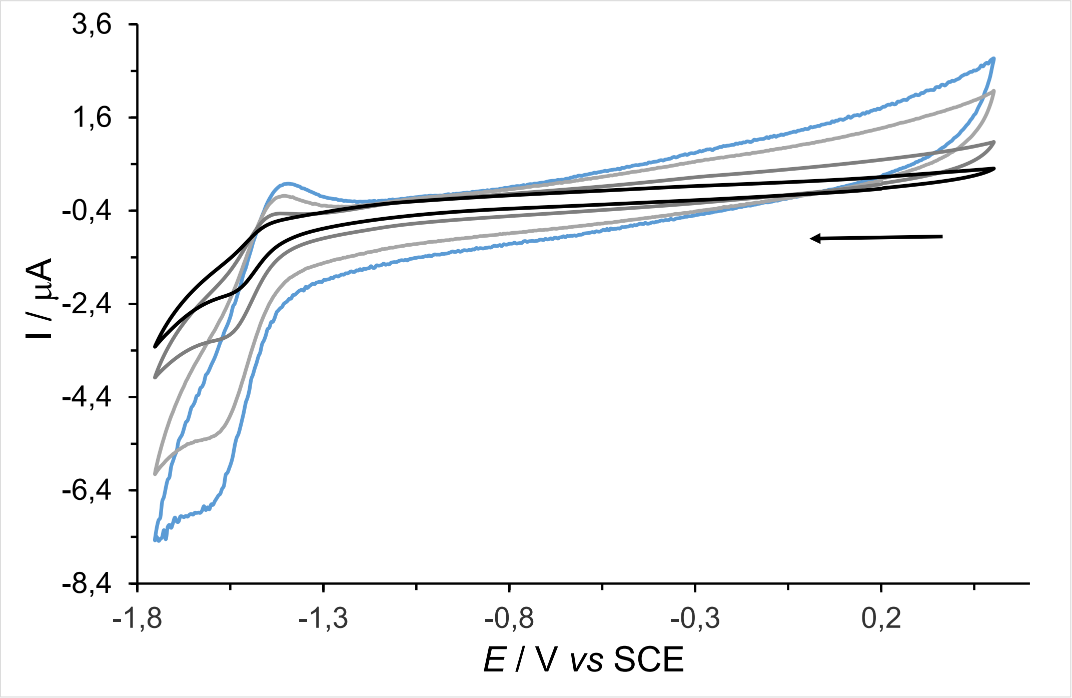

Figure S12c. Cyclic voltammograms of the first reduction process of complexes **7a,b** at respectively, 0.2, 0.5, 1, and 5 V/s respectively, from black line to blue line.

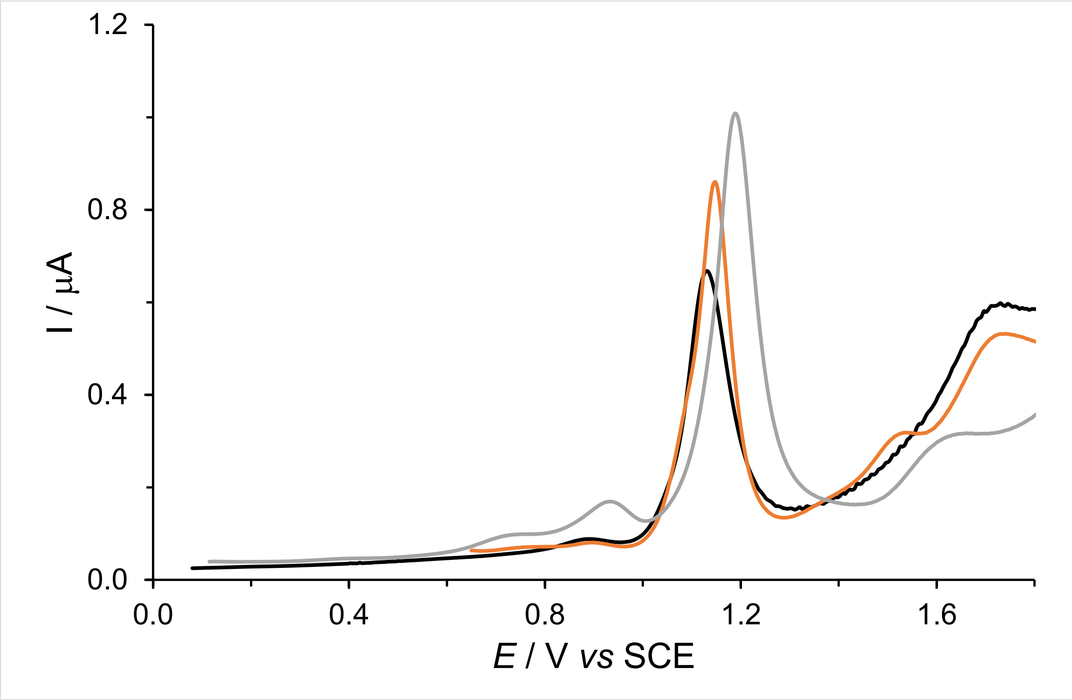

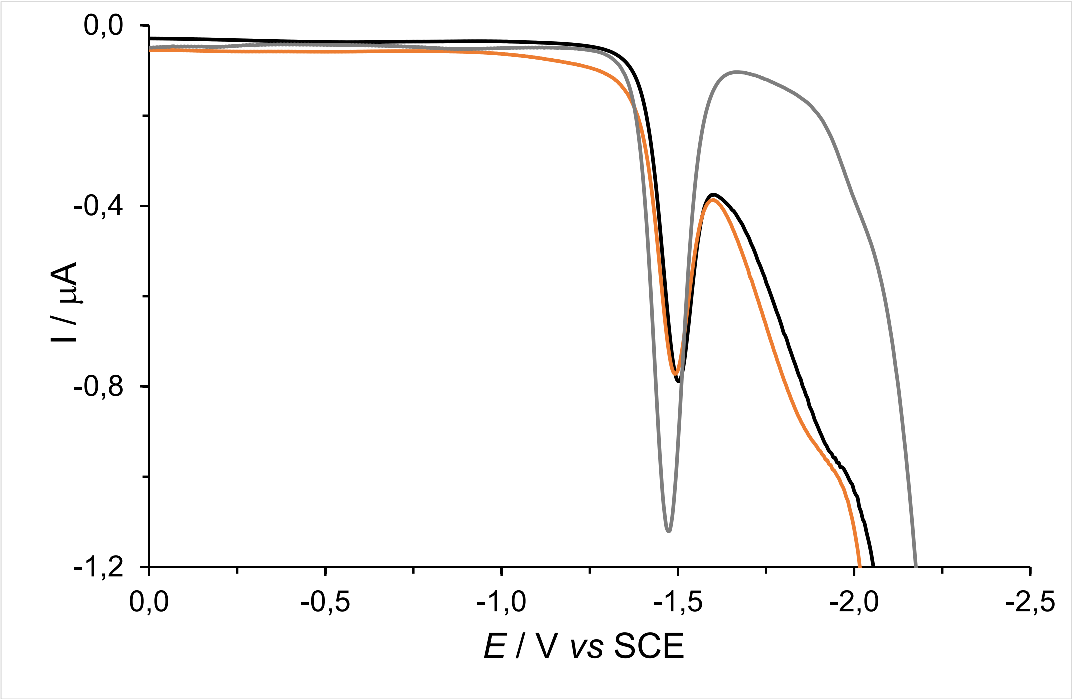

Figure S13. Superimposed OSWVs in oxidation (top) and in reduction (bottom) of complexes **5** (black), **6** (orange) and **7a,b** (gray).

Photophysical measurements

Spectrofluorimetric-grade dichloromethane was used as solvent for spectroscopic investigations. The absorption spectra were recorded with a PerkinElmer Lambda 950 spectrophotometer. For the photoluminescence experiments, the samples were placed in fluorimetric Suprasil quartz cuvettes (1 cm) and deaerated by bubbling argon for at least 20 min. The uncorrected emission spectra were obtained with an Edinburgh Instruments FLS920 spectrometer equipped with a Peltier-cooled Hamamatsu R928 photomultiplier tube (PMT) (185−850 nm). An Edinburgh Xe 900 with 450 W xenon arc lamp was used as the excitation light source. The corrected spectra were obtained via a calibration curve supplied with the instrument. The luminescence quantum yields (PLQY) in solution were obtained from the corrected spectra on a wavelength scale (nm) and measured according to the approach described by Demas and Crosby,[5] using an air-equilibrated water solution of $[Ru(bpy)_3]Cl_2$ (PLQY = 0.028)[6] as reference. The emission lifetimes (τ) in the sub-microsecond time range were measured through the time-correlated single-photon counting (TCSPC) technique using a HORIBA Jobin Yvon IBH FluoroHub controlling a spectrometer equipped with a pulsed NanoLED (λ_{exc} = 373 nm; 200 ps time resolution after reconvolution) as the excitation source and a red-sensitive Hamamatsu R-3237−01 PMT as the detector (spectral window: 185−850 nm). The analysis of the luminescence decay profiles, taken on the emission maximum, was accomplished with the DAS6 Decay Analysis Software provided by the manufacturer, and the quality of the fit was assessed with the χ^2 value close to unity and with the residuals randomly distributed along the time axis. To record the 77 K luminescence spectra, sample solutions were put in quartz tubes (2 mm inner diameter) and inserted into a special quartz Dewar flask filled with liquid nitrogen, and SpectraLED (λ_{exc} = 370 nm; fwhm = 15 nm) was used as excitation source.

Solid state measurements were carried out on PMMA films containing 1 wt % of the complex drop-cast from dichloromethane solutions and placed in between two quartz slides. The thickness of the films was not determined. Solid-state PLQY values were calculated by corrected emission spectra obtained from an Edinburgh FLS920 spectrometer equipped with a barium sulfate-coated integrating sphere (diameter of 3 in.) following the procedure described by Würth et al.[7]

Table S1. Absorption spectra data of complexes **5**, **6** and **7a,b** in in CH_2Cl_2 at 298 K.

Complex	Type of NN ligand	λ (nm)	ε ($M^{-1}cm^{-1}$)
**5**	phen	272, 294, 430	31600, 19300, 4700
**6**	bpy	298, 421	24400, 4100
**7a,b**	mmp	274, 294, 422	33900, 22900, 4700

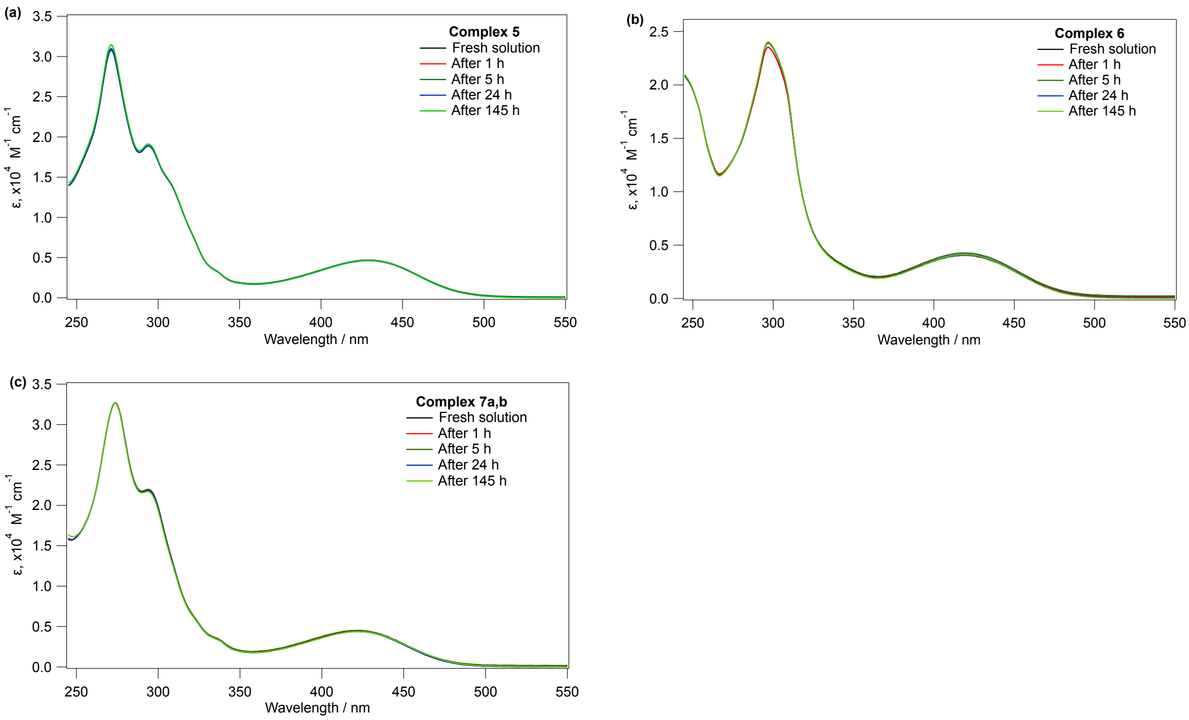

Figure S14. Absorption spectra of (a) [(Cu(phen)(**4c**)]BF_4 (**5**); (b) [(Cu(bpy)(**4c**)]BF_4 (**6**); (c) [(Cu(mmp)(**4c)**]BF_4 (**7a,b**), in CH_2Cl_2 solution at 298 K, taken at several time intervals after sample solubilization.

X-ray crystal structure

X-Ray crystal structure of compound 8.

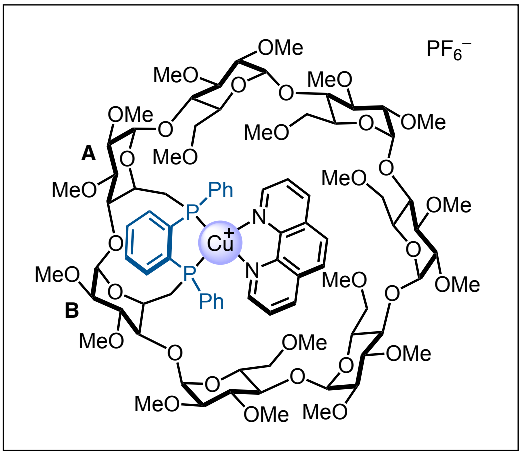

Crystals suitable for X-ray crystal-structure analysis were obtained by slow diffusion of diethyl ether into a CH_2Cl_2 solution of compound **8**. Data were collected at 173(2) K on a Bruker APEX-II Duo KappaCCD diffractometer (Cu-Kα radiation, λ = 1.54178 Å). The structure was solved by direct methods (SHELXS-97) and refined against F^2 using the SHELXL-2014 software.[8] The non-hydrogen atoms were refined anisotropically, using weighted full-matrix least-squares on F^2. The H-atoms were included in calculated positions and treated as riding atoms using SHELXL default parameters. Crystallographic data: formula: $(C_{91}H_{128}O_{33}P_3N_2F_6Cu).(CH_2Cl_2)_4$ (M = 2388.10 g.mol^{-1}); yellow crystals, 0.50 × 0.18 × 0.12 mm; crystal system: monoclinic, space group $P2_1$; a = 13.8620(16) Å; b = 21.453(2) Å; c = 20.274(2) Å; $\alpha = \gamma$ = 90°, β = 93.062(3)°; V = 6020.4(12) Å^3; Z = 2; F(000) = 2496; a total of 171650 reflections collected; 1.899° < θ < 28.026°, 28921 independent reflections with 14084 having I > 2σ(I); 1353 parameters; Final results : $R_1(F^2)$ = 0.0807; $wR_2(F^2)$ = 0.1814, Goof = 1.006.

The Cu complex co-crystallizes with four molecules of CH_2Cl_2, three of them being disordered over two positions. Residual electronic density is due to the presence of two heavily disordered CH_2Cl_2, the contribution of which was taken out by the SQUEEZE procedure in the final refinement.[9] The Flack parameter is 0.054(13), a value in keeping with the $P2_1$ chiral space group.

Full data collection parameters and structural data are available as CIF file (Cambridge Crystallographic Data Center deposition number 2163752).

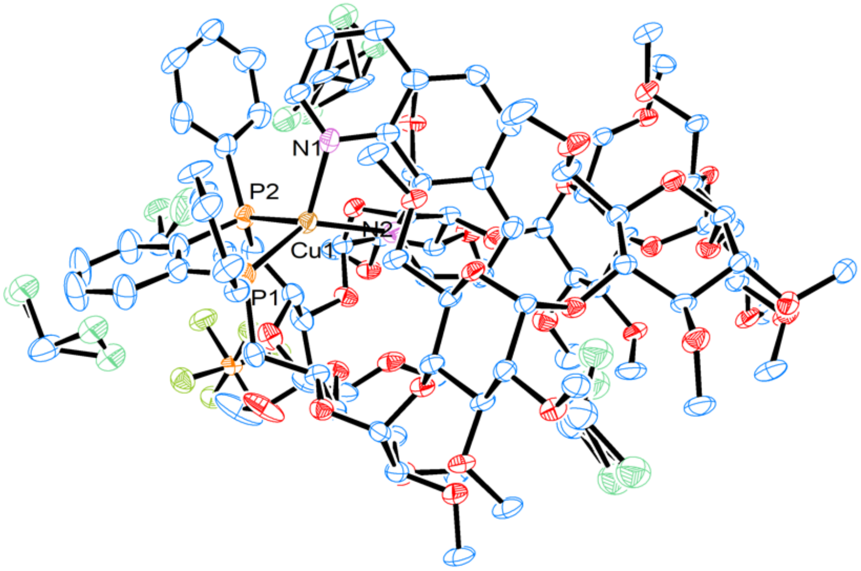

Figure S15. ORTEP representation of the molecular structure of **8**.

References

[1] D. Armspach, L. Poorters, D. Matt, B. Benmerad, F. Balegroune, L. Toupet, *Org. Biomol. Chem.* **2005**, *3*, 2588–2592.

[2] V. Desvergnes-Breuil, V. Hebbe, C. Dietrich-Buchecker, J.-P. Sauvage, J. Lacour, *Inorg. Chem.* **2003**, *42*, 255–257.

[3] a) S. Basra, J. G. de Vries, D. J. Hyett, G. Harrison, K. M. Heslop, A. G. Orpen, P. G. Pringle, K. von der Luehe, *Dalton Trans.* **2004**, 1901-1905; b) I. Bonnaventure, A. B. Charette, *J. Org. Chem.* **2008**, *73*, 6330-6340.

[4] S. Knapp, A. F. Trope, M. S. Theodore, N. Hirata, J. J. Barchi, *J. Org. Chem.* **1984**, *49*, 608–614.

[5] J. N. Demas, G. A. Crosby, *J. Phys. Chem.* **1971**, *75*, 991–1024.

[6] K. Nakamaru, *Bull. Chem. Soc. Jpn.* **1982**, *55*, 2697–2705.

[7] C. Würth, M. Grabolle, J. Pauli, M. Spieles, U. Resch-Genger, *Nat. Protoc.* **2013**, *8*, 1535–1550.

[8] G. M. Sheldrick, *Acta Crystallogr.* **2008**, *A64*, 112-122.

[9] A. L. Spek, *Acta Crystallogr.* **2009**, *D65*, 148–155.
